# Supplementary material for: Enhancing glycan occupancy of soluble HIV-1 envelope trimers to mimic the native viral spike
Source: Cell Rep. Author manuscript; Available in PMC 2022 Feb 1. (PMC8804554; doi:10.1016/j.celrep.2021.108933)
Supplement: 1 [file NIHMS1768927-supplement-1.pdf]

Cell Reports, Volume *35*

Supplemental information

Enhancing glycan occupancy of soluble HIV-1 envelope trimers to mimic the native viral spike

Ronald Derking, Joel D. Allen, Christopher A. Cottrell, Kwinten Sliepen, Gemma E. Seabright, Wen-Hsin Lee, Yoann Aldon, Kimmo Rantalainen, Aleksandar Antanasijevic, Jeffrey Copps, Anila Yasmeen, Albert Cupo, Victor M. Cruz Portillo, Meliawati Poniman, Niki Bol, Patricia van der Woude, Steven W. de Taeye, Tom L.G.M. van den Kerkhof, P.J. Klasse, Gabriel Ozorowski, Marit J. van Gils, John P. Moore, Andrew B. Ward, Max Crispin, and Rogier W. Sanders

Supplementary Figures

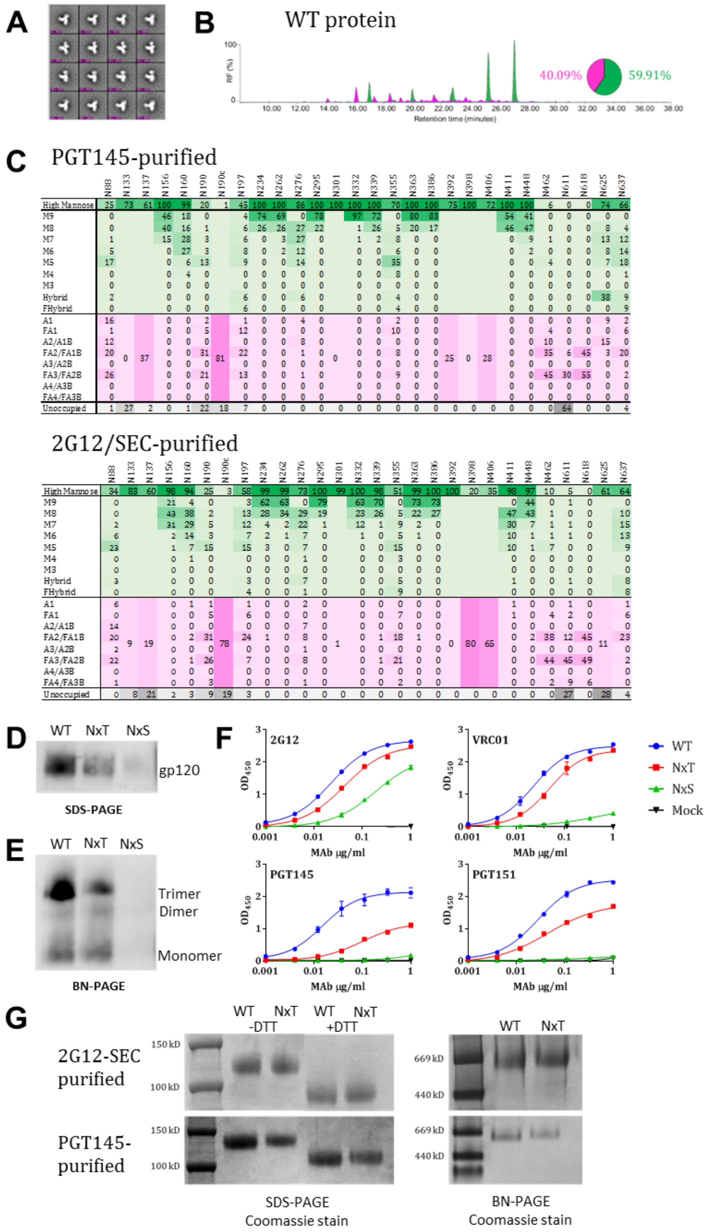

Figure S1. Several PNGS on BG505 SOSIP.v4.1 trimers are underoccupied. Related to Figures 1 and 2. (A) NS-EM analysis of 2G12/SEC-purified WT proteins, showing the 2D class-averages. **(B)** HILIC-UPLC analysis of the 2G12/SEC-purified WT protein. The color coding of the spectra and pie chart is the same as in Figure 1E **(C)** The data sets show the glycoforms found at each PNGS. Data for oligomannose/hybrid-type glycans are shaded in green, fully processed complex type glycans are shaded in magenta, while the absence of a glycan from some PNGS is shaded in grey. Oligomannose-type glycans are categorized according to the number of mannose residues present, hybrids by the presence/absence of fucose and complex-type glycans by the number of processed antenna and the presence/absence of fucose. For further information see Methods. Data that could only be obtained from low intensity peptides cannot be allocated into the above categories. They are merged to cover all oligomannose/hybrid compositions or complex-type glycans. The data presented in this panel are the mean of two independent biological replicates of WT protein. **(D)** Reducing SDS-PAGE analysis of unpurified WT, NxT and NxS proteins expressed in 293T cells, followed by western blotting with the CA13 (ARP3119) MAb. **(E)** BN-PAGE analysis of the same proteins, blotted with the 2G12 bNAb. The trimer, dimer and monomer bands are indicated. **(F)** D7324-capture ELISA quantifying the binding of the bNAbs 2G12, VRC01, PGT145 and PGT151 to the WT, NxT and NxS proteins. **(G)** Non-reducing and reducing (+ DTT) SDS-PAGE followed by Coomassie staining (left panel), and BN-PAGE analysis followed by Coomassie blue staining (right panel) of PGT145- and 2G12/SEC-purified WT and NxT proteins, as indicated.

A **B**

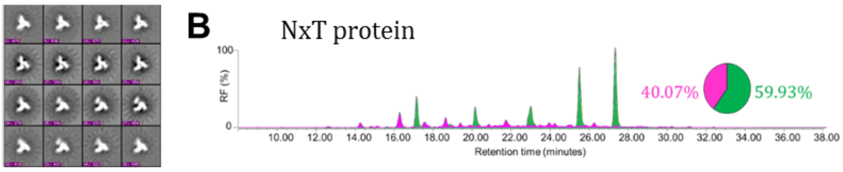

C

PGT145-purified

	N88	N133	N137	N156	N160	N190	N190c	N197	N234	N262	N276	N295	N301	N332	N339	N355	N363	N386	N392	N398	N406	N411	N448	N462	N611	N618	N625	N637
High Mannose	20	53	20	100	88	41	7	41	99	100	77	100	100	100	100	74	100	100	n.d.	n.d.	n.d.	100	100	7	12	0	n.d.	64
M9	0		0	0	0	0		0	70	60	0	76	0	87	70	0	77	100				0	40	0	0	0		0
M8	0		0	23	23	1		3	29	39	16	24	3	13	27	5	20	0				38	45	0	0	0		3
M7	1		0	57	18	4		6	0	0	24	0	20	0	3	12	3	0				0	11	1	0	0		7
M6	4		10	13	21	4		7	0	1	11	0	34	0	0	9	1	0				30	3	0	0	0		12
M5	13		11	6	19	31		7	0	0	13	0	40	0	0	32	0	0				32	1	5	12	0		24
M4	0		0	0	7	0		0	0	0	2	0	0	0	0	7	0	0				0	0	0	0	0		2
M3	0		0	0	0	0		0	0	0	0	0	0	0	0	0	0	0				0	0	0	0	0		0
Hybrid	2		0	0	0	1		11	0	0	11	0	3	0	0	4	0	0				0	0	0	0	0		9
FHybrid	0		0	0	0	0		6	0	0	0	0	0	0	0	4	0	0				0	0	0	0	0		7
A1	6		1	0	0	4		4	0	0	5	0	0	0	0	2	0	0				0	0	2	0	0		2
FA1	1		7	0	0	7		13	0	0	0	0	0	0	0	9	0	0				0	0	6	0	0		13
A2/A1B	14		0	0	0	0		1	0	0	14	0	0	0	0	0	0	0				0	0	1	0	0		0
FA2/FA1B	28	29	49	0	0	28	87	27	0	0	3	0	0	0	0	9	0	0	n.d.	n.d.	n.d.	0	0	42	16	34	n.d.	19
A3/A2B	0		0	0	0	0		0	0	0	0	0	0	0	0	0	0	0				0	0	0	0	0		0
FA3/FA2B	31		21	0	0	19		14	0	0	1	0	0	0	0	7	0	0				0	0	43	63	66		0
A4/A3B	0		0	0	0	0		0	0	0	0	0	0	0	0	0	0	0				0	0	0	0	0		0
FA4/FA3B	0		0	0	0	0		0	0	0	0	0	0	0	0	0	0	0				0	0	0	0	0		0
Unoccupied	1	17	0	0	12	1	5	0	1	0	0	0	0	0	0	0	0	0	n.d.	n.d.	n.d.	0	0	0	9	0	n.d.	1

2G12/SEC-purified

	N88	N133	N137	N156	N160	N190	N190c	N197	N234	N262	N276	N295	N301	N332	N339	N355	N363	N386	N392	N398	N406	N411	N448	N462	N611	N618	N625	N637
High Mannose	27	60	57	100	68	37	0	48	100	100	88	100	100	100	100	79	100	100	100	0	7	100	100	14	31	0	0	71
M9	0		0	0	0	0		1	72	74	0	77	0	80	65	0	76	82		0		0	44	0	0	0	0	0
M8	1		15	30	28	3		7	28	26	25	23	53	17	35	8	20	18		0		34	45	1	0	0	0	7
M7	2		10	70	12	9		6	0	0	26	0	27	1	0	16	3	0		0		30	7	2	0	0	0	11
M6	6		17	0	13	8		9	0	0	16	0	0	1	0	11	1	0		0		20	2	1	6	0	0	17
M5	16		14	0	10	14		8	0	0	15	0	20	1	0	30	0	0		0		16	1	8	25	0	0	21
M4	0		0	0	5	0		0	0	0	3	0	0	0	0	7	0	0		0		0	0	0	0	0	0	1
M3	0		0	0	0	0		0	0	0	0	0	0	0	0	0	0	0		0		0	0	0	0	0	0	0
Hybrid	2		0	0	0	1		10	0	0	4	0	0	0	0	4	0	0		0		0	0	1	0	0	0	9
FHybrid	0		0	0	0	1		6	0	0	0	0	0	0	0	4	0	0		0		0	0	1	0	0	0	6
A1	6		0	0	0	4		3	0	0	0	0	0	0	0	1	0	0		0		0	0	1	0	0	0	2
FA1	1		5	0	0	9		10	0	0	0	0	0	0	0	7	0	0		0		0	0	6	0	0	0	10
A2/A1B	11		0	0	0	0		1	0	0	7	0	0	0	0	0	0	0		0		0	0	1	0	0	0	0
FA2/FA1B	24	0	26	0	0	28	100	27	0	0	5	0	0	0	0	7	0	0	0	0	93	0	0	43	17	42	27	15
A3/A2B	0		0	0	0	0		0	0	0	0	0	0	0	0	0	0	0		0		0	0	0	0	0	0	0
FA3/FA2B	29		11	0	0	20		11	0	0	0	0	0	0	0	5	0	0		100		0	0	36	39	58	73	0
A4/A3B	0		0	0	0	0		0	0	0	0	0	0	0	0	0	0	0		0		0	0	0	0	0	0	0
FA4/FA3B	0		0	0	0	0		0	0	0	0	0	0	0	0	0	0	0		0		0	0	0	0	0	0	0
Unoccupied	1	40	0	0	32	2	0	1	0	0	0	0	0	0	0	0	0	0	0	0	0	0	0	0	14	0	0	3

D

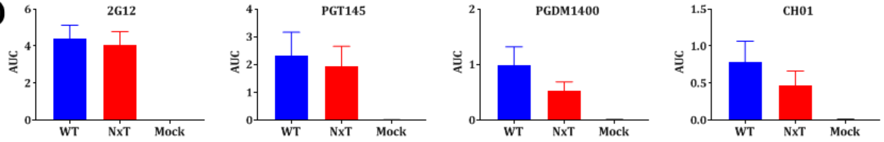

E

PGT145/ PGT130 Fab binding to WT protein/NxT protein, conformational-change model

Fab	Envelope protein	k_{on} (1/Ms)	k_{off} (1/s)	k_f (1/s)	k_b (1/s)	K_D (nM)	K_F	$K_{D\,(conf)}$ (nM)	S_m
PGT145	WT protein (n=2)	$1.1\cdot10^5$ ±0	$7.3\cdot10^{-3}$ $\pm1.3\cdot10^{-3}$	$2.6\cdot10^{-2}$ $\pm1.3\cdot10^{-2}$	$1.1\cdot10^{-3}$ $\pm2.4\cdot10^{-4}$	66 ±11	23 ±7.1	2.8 ±0.42	0.99 $\pm1.1\cdot10^{-2}$
	NxT protein (n=4)	$1.1\cdot10^5$ $\pm1.4\cdot10^4$	$2.2\cdot10^{-2}$ $\pm7.4\cdot10^{-3}$	$3.5\cdot10^{-2}$ $\pm6.2\cdot10^{-3}$	$4.0\cdot10^{-4}$ $\pm6.9\cdot10^{-5}$	$1.9\cdot10^2$ ±43	$1.0\cdot10^2$ ±35	2.0 ±0.18	0.89 $\pm1.4\cdot10^{-2}$
PGT130	WT protein (n=2)	$4.0\cdot10^4$ ±0	$6.9\cdot10^{-3}$ $\pm1.5\cdot10^{-4}$	$7.6\cdot10^{-4}$ $\pm8.5\cdot10^{-5}$	$1.2\cdot10^{-3}$ $\pm2.0\cdot10^{-4}$	$1.7\cdot10^2$ ±3.8	0.64 $\pm3.5\cdot10^{-2}$	$1.0\cdot10^2$ ±2.2	2.8 ±0
	NxT protein (n=2)	$4.1\cdot10^4$ $\pm4.0\cdot10^3$	$6.3\cdot10^{-3}$ $\pm3.0\cdot10^{-4}$	$1.1\cdot10^{-3}$ $\pm1.2\cdot10^{-4}$	$1.5\cdot10^{-3}$ $\pm1.5\cdot10^{-4}$	$1.5\cdot10^2$ ±23	0.74 $\pm5.8\cdot10^{-3}$	91 ±14	2.7 $\pm6.1\cdot10^{-2}$

F

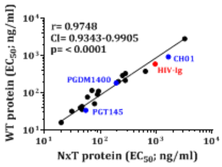

Figure S2. Glycan occupancy is enhanced by PNGS sequon engineering. Related to Figure 2. (A) NS-EM analysis of NxT proteins, showing the 2D class-averages. **(B)** HILIC-UPLC analysis of the NxT protein. The color coding of the spectra and pie chart is the same as in Figure S1B. **(C)** Quantification of site-specific occupancy and composition for 28 PNGS on NxT trimers, purified using the 2G12/SEC and PGT145 methods as indicated. The data are derived from LC-ESI MS experiments. The data set shows the glycoforms found at each PNGS. The relative under-occupancy and oligomannose and complex/hybrid content at each individual site are summarized, using the same color coding as in Figure S1C. **(D)** Binding of WT and NxT proteins to three V2-apex directed bNAbs, PGT145, PGDM1400 and CH01, and also 2G12 for comparison. AUC values derived from derived from ELISA titration curves are plotted. **(E)** Summary of the SPR binding kinetics of bNAbs PGT145 and PGT130 using data shown in Figure 2F. **(F)** The EC_{50} values derived using WT and NxT proteins were plotted and compared using Spearman's correlation coefficient. Binding data were generated for a panel of bNAbs spanning all major bNAb epitope clusters (Figure S3). All analyses were performed on NxT proteins produced in HEK293F followed by 2G12/SEC purification.

A

			Binding EC_{50} (ng/ml) PGT145 purified protein			Binding (AUC) PGT145 purified protein		
	Epitope	Antibody	WT protein	NxT protein	Fold difference	WT protein	NxT protein	Fold difference
Nabs	CD4bs	VRC01	66	100	1.5	3.2	2.9	0.9
		3BNC60	57	81	1.4	3.2	2.8	0.9
	V1/V2-glycan	PG9	388	359	0.9	3.0	2.7	0.9
		PG16	144	109	0.8	2.0	1.9	1.0
		PGT145	40	74	1.9	2.8	2.2	0.8
		PGDM1400	206	585	2.8	1.1	0.5	**0.5**
		CH01	372	906	2.4	2.2	1.4	**0.6**
	V3-glycan	PGT121	316	417	1.3	2.8	2.5	0.9
		PGT122	577	799	1.4	2.3	2.0	0.8
		PGT123	232	396	1.7	3.2	2.7	0.8
		PGT125	150	115	0.8	3.5	3.4	1.0
		PGT126	113	84	0.7	3.1	2.8	0.9
		PGT128	45	73	1.6	3.4	3.0	0.9
		PGT130	193	133	0.7	2.1	1.8	0.9
	OD-glycan	2G12	30	40	1.3	4.1	3.6	0.9
		PGT135	234	601	2.6	4.0	2.8	0.7
	gp120-gp41	PGT151	29	23	0.9	3.1	2.8	0.9
		35O22	814	2111	2.6	2.5	2.0	0.8
	gp41	3BC315	62	69	1.1	2.7	1.9	0.7
		HIV-IG	>10000	>10000	1.0	0.4	0.3	0.9

B

			Binding EC_{50} (ng/ml) 2G12/SEC purified protein			Binding (AUC) 2G12/SEC purified protein		
	Epitope	Antibody	WT protein	NxT protein	Fold difference	WT protein	NxT protein	Fold difference
Nabs	CD4bs	VRC01	36	47	1.3	3.6	3.3	0.9
		3BNC60	91	89	1.0	3.0	2.9	1.0
	V1/V2-glycan	PG9	201	211	1.1	2.9	3.1	1.1
		PG16	77	59	0.8	2.1	2.2	1.0
		PGT145	34	55	1.6	2.3	1.9	0.8
		PGDM1400	185	193	1.0	1.0	0.5	**0.5**
		CH01	923	1656	1.8	0.8	0.5	**0.6**
	V3-glycan	PGT121	291	250	0.9	2.3	2.4	1.0
		PGT122	333	282	0.8	2.0	1.7	0.9
		PGT123	216	280	1.3	2.2	1.8	0.9
		PGT125	51	79	1.5	3.5	3.3	0.9
		PGT126	32	31	1.0	4.1	4.1	1.0
		PGT128	43	47	1.1	4.1	3.8	0.9
		PGT130	111	93	0.8	2.0	1.8	0.9
	OD-glycan	2G12	16	20	1.3	4.4	4.0	0.9
		PGT135	379	644	1.7	3.7	2.7	0.8
	gp120-gp41	PGT151	40	44	1.1	1.8	1.5	0.8
		35O22	2829	3254	1.2	1.1	1.0	0.9
	gp41	3BC315	116	71	**0.6**	2.4	2.0	0.9
		HIV-IG	595	960	1.6	3.1	2.8	0.9

Figure. S3. Antigenic characterization of PGT145- and 2G12/SEC-purified WT and NxT proteins. Related to Figure 2. (A) Half-maximal binding concentrations (EC_{50}; in ng/ml) were derived from D7324-capture ELISAs using PGT145-purified WT and NxT proteins. The values represent the means of 4–10 independent single titration experiments for each bNAb. The fold-differences in EC_{50} values for NxT *vs.*WT proteins are listed. We also tabulated the average AUC values derived from the titration curves for each MAb. Bold numbers indicate values where the differences are <0.6- or >3-fold. **(B)** The same analysis and data presentation but derived using 2G12/SEC-purified WT and NxT proteins.

A

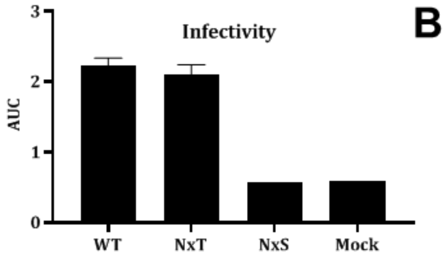

Virus	WT	NxT	NxS
CA-p24 (ng/ml)	127	1106	684

B

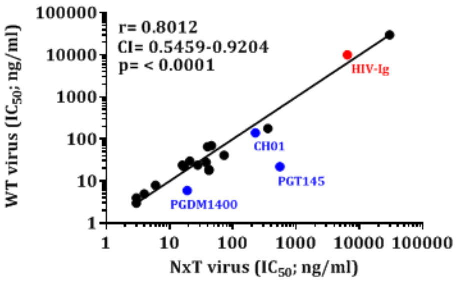

C

	Epitope	Antibody	Neutralization IC_{50} (ng/ml) WT virus	NxT virus	Fold difference	Neutralization (AUC) WT virus	NxT virus	Fold difference
Nabs	CD4bs	VRC01	66	40	0.6	238.2	200.7	0.8
		3BNC60	30	21	0.7	224.1	184.5	0.8
	V1/V2-glycan	PG9	19	42	2.2	155.0	181.2	1.2
		PG16	18	42	2.4	156.3	208.3	1.3
		PGT145	22	554	**25.7**	157.6	266.6	1.7
		PGDM1400	6	19	**3.1**	126.2	198.7	1.6
		CH01	140	227	1.6	203.8	279.1	1.4
	V3-glycan	PGT121	24	16	0.7	158.3	136.1	0.9
		PGT122	41	73	1.8	203.5	204.5	1.0
		PGT123	22	17	0.8	189.7	178.8	0.9
		PGT125	5	4	0.8	113.7	99.6	0.9
		PGT126	8	6	0.7	143.6	133.5	0.9
		PGT128	4	3	0.9	107.9	100.0	0.9
		PGT130	28	38	1.4	156.0	159.8	1.0
	OD-glycan	2G12	24	28	1.2	125.5	135.9	1.1
		PGT135	179	359	2.0	218.0	232.0	1.1
	gp120-gp41	PGT151	3	3	1.1	88.2	107.7	1.2
		35O22	70	46	0.6	144.7	117.8	0.8
	gp41	3BC315	>150000	>150000	1.0	359.3	372.1	1.0
		HIV-Ig	10086	6437	1.6	405.7	352.7	0.9

D

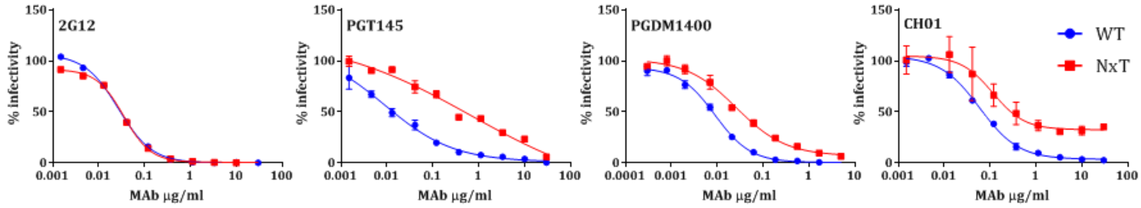

Figure S4. NxT sequon engineering is compatible with Env function and virus infectivity. Related to Figure 2. (A) Infection of TZM-bl reporter cells by WT, NxT and NxS viruses using a range of p24 concentrations (500, 250, 125, 62.5, 31.25, 16.62 and 7.81 pg). The AUC values derived from the titration curve were plotted. The box represents CA-p24 measured in each virus preparation. **(B)** Correlation between neutralization of WT and NxT viruses. The midpoint neutralization concentrations (IC_{50}) were plotted and the Spearman's correlation coefficient, r, was calculated. The outliers, PGT145, PGDM1400 and CH01, are indicated in blue. Polyclonal HIV-Ig is indicated in red. **(C)** Midpoint neutralization concentrations (IC_{50}; in ng/ml) were derived from single cycle infection experiments using TZM-bl cells and he indicated bNAbs or HIV-Ig. The values are averages based on 2-4 independent antibody-titration experiments. The average AUC values derived from the neutralization curves are also shown, in the three right-most columns. **(D)** Representative neutralization curves for the WT and NxT virus and the 2G12, PGT145, PGDM1400 and CH01 bNAbs.

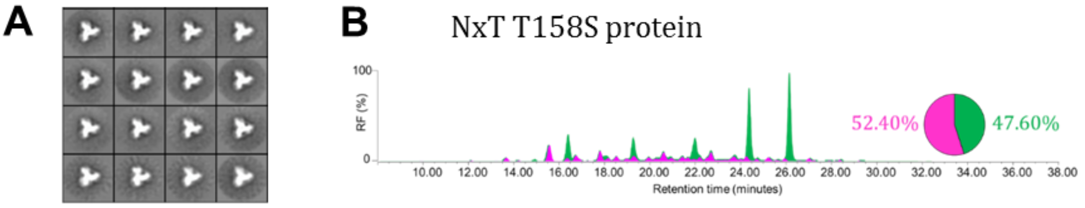

C

PGT145-purified

	N88	N133	N137	N156	N160	N190	N190c	N197	N234	N262	N276	N295	N301	N332	N339	N355	N363	N386	N392	N398	N406	N411	N448	N462	N611	N618	N625	N637
High Mannose	11	65	17	100	100	25	4	38	100	100	63	100	98	100	100	64	100	100	100	n.d	99	100	100	7	10	0	3	51
M9	0	1	0	2	0	0		0	69	69	0	77	0	81	67	0	78	69				0	33	0	0	0	0	0
M8	0	22	1	25	10	1		3	25	29	5	23	28	15	28	3	19	26				22	35	0	0	0	0	1
M7	1	15	1	39	19	4		5	3	2	6	0	39	4	5	9	2	5				33	20	1	2	0	0	4
M6	2	12	1	14	25	4		3	2	0	5	0	12	1	1	11	1	0				24	8	1	0	0	0	7
M5	6	13	1	16	31	13		10	0	0	5	0	14	0	0	24	0	0				19	3	4	8	0	3	11
M4	0	1	0	1	12	0		0	0	0	2	0	0	0	0	9	0	0				0	1	0	0	0	0	1
M3	0	0	0	0	3	0		0	0	0	0	0	0	0	0	0	0	0				0	0	0	0	0	0	0
Hybrid	2	2	9	1	0	2		8	0	0	38	0	3	0	0	6	0	0				2	0	1	0	0	0	11
FHybrid	1	0	5	0	0	1		7	0	0	2	0	1	0	0	2	0	0				0	0	1	0	0	0	15
A1	3	1	0	0	0	1		2	0	0	3	0	0	0	0	0	0	0				0	0	1	0	0	0	1
FA1	1	0	2	0	0	4		9	0	0	0	0	0	0	0	26	0	0				0	0	3	0	0	0	8
A2/A1B	7	0	4	0	0	0		1	0	0	26	0	0	0	0	0	0	0				0	0	5	0	0	1	0
FA2/FA1B	22	4	36	0	0	33	96	34	0	0	4	0	1	0	0	5	0	0	0	n.d.	1	0	0	38	10	25	29	31
A3/A2B	2	0	17	0	0	0		0	0	0	3	0	0	0	0	0	0	0				0	0	1	0	0	0	0
FA3/FA2B	39	4	20	0	0	32		15	0	0	0	0	1	0	0	4	0	0				0	0	40	59	45	46	6
A4/A3B	0	0	0	0	0	0		0	0	0	0	0	0	0	0	0	0	0				0	0	0	0	0	0	0
FA4/FA3B	14	0	4	0	0	4		0	0	0	0	0	0	0	0	0	0	0				0	0	5	13	29	21	0
Unoccupied	1	26	0	0	0	1	0	0	0	0	0	0	0	0	0	0	0	0	0	n.d	n.d	0	0	0	8	0	0	2

2G12/SEC-purified

	N88	N133	N137	N156	N160	N190	N190c	N197	N234	N262	N276	N295	N301	N332	N339	N355	N363	N386	N392	N398	N406	N411	N448	N462	N611	N618	N625	N637
High Mannose	17	75	n.d.	100	75	30	n.d.	40	95	100	31	100	n.d.	99	88	73	97	45	0	n.d.	n.d.	100	94	11	24	1	69	66
M9	0	0		20	2	0		1	61	65	0	63		78	56	0	73	32	0			0	37	0	0	0	0	0
M8	1	24		37	45	3		5	28	28	0	29		18	24	8	17	12	0			35	38	1	1	0	14	6
M7	2	19		30	15	6		5	4	3	1	3		2	5	14	3	0	0			32	10	2	4	0	13	9
M6	4	11		7	4	4		4	2	2	2	1		0	1	9	1	0	0			19	3	1	3	0	9	13
M5	9	17		5	3	13		10	1	1	4	2		0	0	26	1	0	0			13	3	6	13	0	7	16
M4	0	1		1	0	0		0	0	0	2	1		0	0	1	0	0	0			0	1	0	0	0	0	1
M3	0	0		0	0	0		0	0	0	0	0		0	0	0	0	0	0			0	0	0	0	0	0	0
Hybrid	2	2		0	5	2		7	0	0	20	0		0	0	6	0	0	0			2	1	1	2	0	25	10
FHybrid	1	1		0	1	2		7	0	0	3	0		0	1	8	0	1	0			0	1	1	1	0	2	11
A1	2	1		0	2	1		2	0	0	1	0		0	0	1	0	0	0			0	0	1	1	0	2	1
FA1	1	1		0	0	5		10	0	0	1	0		0	0	7	0	0	0			0	1	3	2	0	3	8
A2/A1B	8	0		0	0	0		1	0	0	13	0		0	0	0	0	2	0			0	0	1	0	0	10	0
FA2/FA1B	22	5	n.d.	0	15	30	n.d.	33	4	0	21	0	n.d.	0	5	11	2	23	2	n.d.	n.d.	0	4	43	13	22	7	19
A3/A2B	3	0		0	0	0		0	0	0	2	0		0	0	0	0	0	0			0	0	1	0	0	2	0
FA3/FA2B	34	4		0	7	29		13	1	0	25	0		0	6	7	1	27	65			0	1	36	44	46	6	5
A4/A3B	0	0		0	0	0		0	0	0	0	0		0	0	0	0	0	0			0	0	0	0	0	0	0
FA4/FA3B	11	0		0	0	4		1	0	0	4	0		0	1	1	0	4	33			0	0	5	12	31	0	1
Unoccupied	1	13	n.d	0	0	1	n.d.	0	0	0	0	0	n.d.	0	0	0	0	0	0	n.d	n.d	0	0	0	3	0	0	0

Figure. S5. N160 occupancy can be increased by reducing the affinity of a neighboring site for OST. Related to Figure 3. (A) NS-EM analysis of NxT 158S trimers, showing the 2D class-averages. **(B)** HILIC-UPLC analysis of the NxT T158S protein. The color coding of the spectra and pie chart is the same as in Figure S1B. **(C)** Quantification of site-specific occupancy and composition for 28 PNGS on NxT T158S proteins, purified using the 2G12/SEC and PGT145 methods as indicated. The data are derived from LC-ESI MS experiments. The data set shows the glycoforms found at each PNGS. The relative under-occupancy and oligomannose and complex/hybrid content at each individual site are summarized, using the same color coding as in Figure S1C.

A

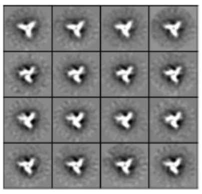

B PGT145-purified

	N88	N133	N137	N156	N160	N190	N190c	N197	N234	N262	N276	N295	N301	N332	N339	N355	N363	N386	N392	N398	N406	N411	N448	N462	N611	N618	N625	N637
High Mannose	10	90	65	100	100	0	0	30	99	100	76	100	98	100	93	61	100	100	n.d.	n.d.	36	100	100	3	6	0	n.d.	52
M9	0			37	0			0	70	63	0	88		91	61	0	77	81				0	48	0	0	0		0
M8	0			46	33			1	24	29	10	12		8	27	3	21	19				45	39	0	0	0		0
M7	1			12	17			3	3	4	16	0		1	3	8	2	0				15	8	0	1	0		6
M6	2			3	20			3	2	4	15	0		0	2	7	1	0				26	4	0	1	0		5
M5	6			2	31			10	0	0	19	0		0	0	24	0	0				14	1	2	4	0		7
M4	0			0	0			0	0	0	2	0		0	0	1	0	0				0	0	0	0	0		1
M3	0			0	0			0	0	0	0	0		0	0	0	0	0				0	0	0	0	0		0
Hybrid	1			1	0			6	0	0	14	0		0	0	6	0	0				0	0	0	0	0		6
FHybrid	1			0	0			7	0	0	1	0		0	0	11	0	0				0	0	1	0	0		27
A1	3			0	0			2	0	0	5	0		0	0	1	0	0				0	0	0	0	0		1
FA1	1			0	0			11	0	0	0	0		0	0	15	0	0				0	0	4	1	0		10
A2/A1B	10			0	0			1	0	0	14	0		0	0	0	0	0				0	0	1	0	0		0
FA2/FA1B	29	5	35	0	0	100	100	38	0	0	2	0	2	0	0	12	0	0	n.d.	n.d.	64	0	0	43	14	28	n.d.	27
A3/A2B	5			0	0			0	0	0	2	0		0	0	0	0	0				0	0	0	0	0		0
FA3/FA2B	35			0	0			16	0	0	0	0		0	0	9	0	0				0	0	43	55	60		9
A4/A3B	0			0	0			0	0	0	0	0		0	0	0	0	0				0	0	0	0	0		0
FA4/FA3B	8			0	0			1	0	0	0	0		0	0	1	0	0				0	0	5	20	10		0
Unoccupied	0	6	0	0	0	0	0	1	1	0	0	0	0	0	7	0	0	0	n.d.	n.d.	0	0	0	0	3	2	n.d.	2

2G12/SEC-purified

	N88	N133	N137	N156	N160	N190	N190c	N197	N234	N262	N276	N295	N301	N332	N339	N355	N363	N386	N392	N398	N406	N411	N448	N462	N611	N618	N625	N637
High Mannose	7	62	39	99	99	25	0	35	80	99	36	100	100	100	87	68	98	85	n.d.	n.d.	0	100	92	7	10	1	65	60
M9	0			24	9	0		1	52	66	0			82	64	0	77	68				0	39	0	0	0		0
M8	0			33	27	2		3	18	25	3			14	19	6	16	17				38	35	0	1	0		5
M7	0			27	21	3		6	4	4	6			2	3	13	3	0				29	11	1	2	0		7
M6	1			7	15	3		3	3	3	4			1	0	9	1	0				16	4	1	1	0		1
M5	5			6	19	17		11	2	2	4			2	0	26	1	0				17	2	4	6	0		29
M4	0			0	7	0		0	0	0	1			0	0	1	0	0				0	0	0	0	0		0
M3	0			0	1	0		0	0	0	0			0	0	0	0	0				0	0	0	0	0		0
Hybrid	0			1	0	1		6	0	0	15			0	0	6	0	0				0	1	0	0	0		7
FHybrid	1			0	0	0		5	1	0	2			0	1	6	0	0				0	1	1	1	1		10
A1	0			1	0	1		2	0	0	2			0	0	1	0	0				0	0	1	1	0		3
FA1	2			0	0	5		10	1	0	1			0	0	10	0	0				0	1	5	8	0		14
A2/A1B	8			0	0	0		1	0	0	11			0	0	0	0	0				0	0	2	0	0		0
FA2/FA1B	31	0	60	1	1	18	100	35	12	1	19	0	0	0	7	10	1	7	n.d.	n.d.	91	0	5	41	15	41	21	21
A3/A2B	3			0	0	0		0	0	0	4			0	0	0	0	0				0	0	0	0	0		0
FA3/FA2B	41			0	0	33		17	7	0	23			0	6	7	0	9				0	2	40	49	46		1
A4/A3B	0			0	0	0		0	0	0	0			0	0	0	0	0				0	0	0	0	0		0
FA4/FA3B	7			0	0	3		1	1	0	5			0	1	3	0	0				0	0	4	15	12		0
Unoccupied	0	37	1	0	0	14	0	0	0	0	0	0	0	0	0	0	0	0	n.d.	n.d.	0	0	0	0	0	0	13	1

C

CHO BG505 WT	N88	N133	N137	N156	N160	N190	N190c	N197	N234	N262	N276	N295	N301	N332	N339	N355	N363	N386	N392	N398	N406	N411	N448	N462	N611	N618	N625	N637
High Mannose	2	83	69	92	100	0	0	19	100	100	99	100	100	100	92	68	100	100	n.d.	n.d.	0	99	100	0	0	n.d.	0	38
Complex	98	0	15	0	0	8	62	48	0	0	1	0	0	0	0	32	0	0	n.d.	n.d.	100	1	0	100	37	n.d.	0	45
Unoccupied	0	17	16	8	0	92	38	34	0	0	0	0	0	0	8	0	0	0	n.d.	n.d.	0	0	0	0	63	n.d.	100	16
**CHO BG505 NxT**																												
High Mannose	0	86	66	100	100	0	0	0	100	100	92	100	100	100	100	88	100	100	n.d.	n.d.	n.d.	n.d.	100	0	0	0	0	92
Complex	100	6	18	0	0	93	100	100	0	0	8	0	0	0	0	12	0	0	n.d.	n.d.	n.d.	n.d.	0	100	100	100	0	0
Unoccupied	0	8	16	0	0	7	0	0	0	0	0	0	0	0	0	0	0	0	n.d.	n.d.	n.d.	n.d.	0	0	0	0	100	8
Change in occupancy	0	8	1	8	0	85	38	34	0	0	0	0	0	0	8	0	0	0	n.d.	n.d.	n.d.	n.d.	0	0	63	n.d.	0	8

D

WT change in CHO	N88	N133	N137	N156	N160	N190	N190c	N197	N234	N262	N276	N295	N301	N332	N339	N355	N363	N386	N392	N398	N406	N411	N448	N462	N611	N618	N625	N637
High Mannose	-23	10	8	-8	1	-19	-1	-26	0	0	13	0	0	0	-8	-2	0	0	n.d.	n.d.	-72	-1	0	-6	0	n.d.	-74	-27
Complex	24	0	-23	0	0	-50	-19	0	0	0	-13	0	0	0	0	2	0	0	n.d.	n.d.	72	1	0	6	1	n.d.	-26	15
Unoccupied	-1	-10	14	8	-1	70	20	26	0	0	0	0	0	0	8	0	0	0	n.d.	n.d.	0	0	0	0	-1	n.d.	100	12

NxT change in CHO	N88	N133	N137	N156	N160	N190	N190c	N197	N234	N262	N276	N295	N301	N332	N339	N355	N363	N386	N392	N398	N406	N411	N448	N462	N611	N618	N625	N637
High Mannose	-10	-4	2	0	0	0	0	-30	1	0	16	0	2	0	7	27	0	0	n.d.	n.d.	n.d.	n.d.	0	-3	-6	0	n.d.	41
Complex	11	1	-17	0	0	-7	0	32	0	0	-16	0	-2	0	0	-27	0	0	n.d.	n.d.	n.d.	n.d.	0	3	9	2	n.d.	-46
Unoccupied	0	3	15	0	0	7	0	-1	-1	0	0	0	0	0	-7	0	0	0	n.d.	n.d.	n.d.	n.d.	0	0	-3	-2	n.d.	6

Figure. S6. N133 occupancy is increased by reducing its affinity for OST. Related to Figure 3. (A) NS-EM analysis of NxT T135S T158S proteins, showing the 2D class-averages. **(B)** Quantification of site-specific occupancy and composition for 28 PNGS on NxT T135S T158S proteins, purified using the 2G12/SEC and PGT145 methods as indicated. The data are derived from LC-ESI MS experiments. The data set shows the glycoforms found at each PNGS. The relative under-occupancy and oligomannose and complex/hybrid content at each individual site are summarized, using the same color coding as in Figure S1C. **(C)** Site-specific analysis of the WT and NxT T135S T158S proteins produced in ExpiCHO-S cells. **(D)** Site-specific changes in glycosylation when BG505 SOSIP WT is produced in ExpiCHO-S cells compared to HEK293F cells. A percentage point (p.p.) increase represents a higher abundance of a particular glycoform on ExpiCHO-S cells.

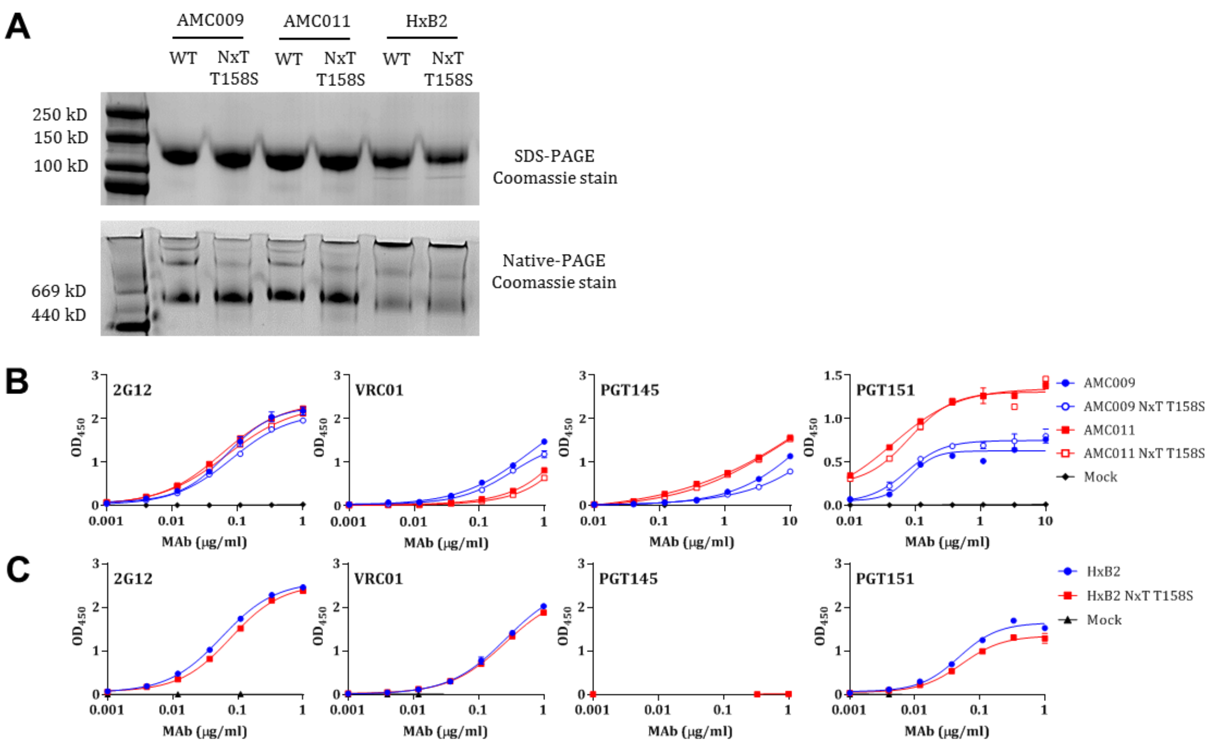

D

AMC009 WT	N88	N130	N136	N139	N141	N156	N160	N189A	N197	N234	N241	N262	N276	N289	N295	N301	N332	N339	N356	N362	N386	N392	N397	N448	N463	N611	N616	N625	N637
High mannose	37	95	54	14	5	90	87	37	42	98	98	100	99	90	100	99	99	100	15	100	100	100	9	100	4	25	26	19	23
Complex	62	5	40	4	91	0	12	62	36	0	0	0	1	0	0	0	1	0	85	0	0	0	91	0	96	16	18	80	12
Unoccupied	1	0	6	82	3	10	1	1	23	2	2	1	0	10	0	1	0	0	0	0	0	0	0	0	0	58	55	1	65
**NxT**																													
High mannose	37	100	70	6	3	88	92	20	22	98	97	100	93	100	100	100	99	100	0	100	100	100	0	100	0	1	2	18	12
Complex	55	0	26	85	94	0	3	75	73	0	2	0	7	0	0	0	0	0	100	0	0	0	100	0	100	84	94	82	18
Unoccupied	8	0	4	9	2	12	5	4	5	1	1	0	0	0	0	0	1	0	0	0	0	0	0	0	0	14	4	0	70
Change in occupancy	-7	0	2	73	1	-2	-4	-4	18	1	1	1	0	10	0	1	-1	0	0	0	0	0	0	0	0	44	51	1	-5

AMC011 WT	N88	N130	N136	N141	N141c	N156	N160	N188	N197	N234	N241	N262	N276	N289	N295	N301	N332	N339	N355	N362	N386	N392	N396	N406	N448	N461	N611	N616	N625	N637
High mannose	34	100	100	14	21	99	99	73	57	98	86	99	97	n.d.	99	99	100	100	22	97	100	n.d.	n.d.	100	98	36	3	3	19	44
Complex	65	0	0	8	79	0	1	26	41	0	0	0	3	n.d.	0	0	0	0	77	0	0	n.d.	n.d.	0	0	63	5	0	75	41
Unoccupied	1	0	0	78	0	1	0	1	2	2	14	1	0	n.d.	1	1	0	0	0	3	0	n.d.	n.d.	0	2	1	92	97	6	15
**NxT**																														
High mannose	50	96	100	18	0	88	99	89	81	99	99	100	29	99	91	95	100	87	23	100	100	100	100	99	100	25	9	14	57	35
Complex	41	1	0	82	79	0	0	8	19	0	1	0	71	0	0	5	0	0	77	0	0	0	0	1	0	75	88	6	39	27
Unoccupied	8	3	0	0	21	12	0	2	0	0	0	0	0	1	9	0	0	13	0	0	0	0	0	0	0	0	3	80	4	38
Change in occupancy	-7	-3	0	78	-21	-12	0	-2	2	2	14	1	0	n.d.	-8	1	0	-13	0	3	0	n.d.	n.d.	0	2	1	89	17	2	-23

HXB2 WT	N88	N136	N141	N156	N160	N189	N197	N230	N234	N241	N262	N276	N289	N295	N301	N322	N339	N356	N386	N392	N397	N406	N411	N448	N463	N611	N616	N624	N637
High Mannose	94	12	0	52	20	6	34	100	96	97	100	75	98	100	100	98	99	25	100	99	n.d.	7	n.d.	98	25	3	2	56	20
Complex	6	88	100	3	3	33	60	0	1	1	0	14	0	0	0	0	0	75	0	1	n.d.	93	n.d.	1	73	43	15	40	39
Unoccupied	0	0	0	45	77	61	5	0	2	2	0	11	1	0	0	1	0	0	0	0	n.d.	0	n.d.	0	2	54	84	3	41
**NxT**																													
High Mannose	86	14	3	54	66	5	30	100	97	96	100	77	100	99	28	95	97	11	99	99	100	0	100	94	11	2	14	58	20
Complex	14	86	97	13	22	42	69	0	3	3	0	20	0	1	70	5	3	89	1	1	0	100	0	6	89	89	68	42	64
Unoccupied	0	0	0	33	11	53	1	0	0	0	0	3	0	0	2	0	0	0	0	0	0	0	0	0	0	9	18	0	16
Change in occupancy	0	0	0	12	66	8	5	0	2	2	0	8	1	0	-2	1	0	0	0	0	n.d.	0	n.d.	0	2	46	65	3	25

Figure. S7. PNGS sequon engineering on diverse Env isolates. Related to Figure 5. (A) Reducing (+ DTT) SDS-PAGE followed by Coomassie staining (top panel), and BN-PAGE analysis followed by Coomassie blue staining (bottom panel) of PGT145-purified AMC009, AMC011 and HxB2 WT and NxT T158S proteins, as indicated. **(B)** D7324-capture ELISA quantifying the binding of the bNAbs 2G12, VRC01, PGT145 and PGT151 to the AMC009, AMC009 NxT T158S, AMC011 and AMC011 NxT T158S proteins. **(C)** Ni-NTA-capture ELISA quantifying the binding of the bNAbs 2G12, VRC01, PGT145 and PGT151 to the HxB2 and HxB2 NxT T158S proteins. **(D)** Glycoforms are grouped for all samples into high mannose (oligomannose- and hybrid-type), complex and unoccupied. The percentage change in occupancy between the WT protein and the NxT T158S protein is also shown.
