# Supplementary material for: Enhancing glycan occupancy of soluble HIV-1 envelope trimers to mimic the native viral spike
Source: Cell Rep. Author manuscript; Available in PMC 2022 Feb 1. (PMC8804554; doi:10.1016/j.celrep.2021.108933)
Supplement: 2 [file NIHMS1768927-supplement-2.pdf]

Cell Reports

Article

Enhancing glycan occupancy of soluble HIV-1 envelope trimers to mimic the native viral spike

Graphical abstract

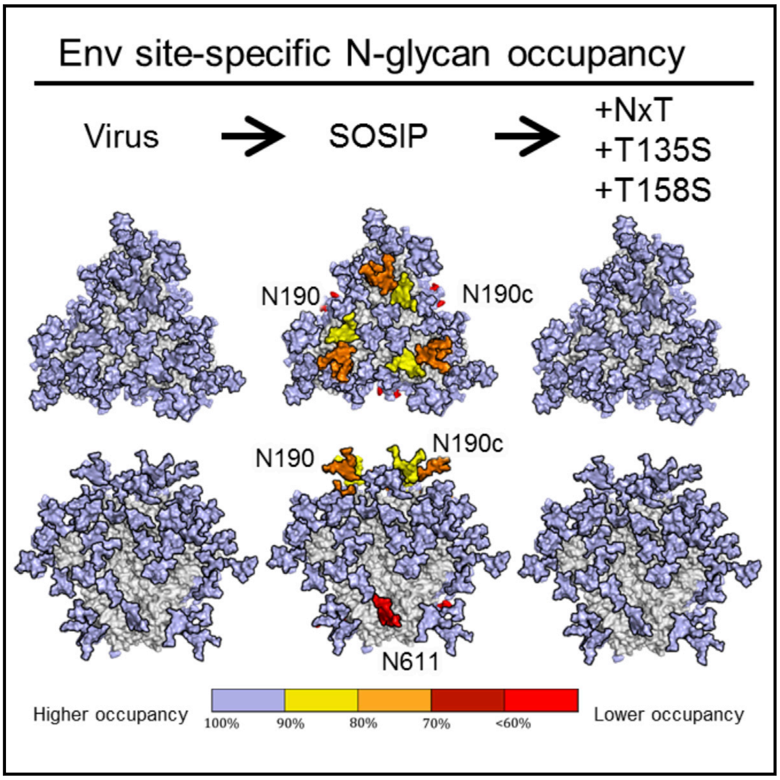

Highlights

- Recombinant HIV-1 envelope trimers can exhibit glycan under-occupancy
- Under-occupancy induces non-neutralizing antibodies to artificial glycan holes
- Glycan occupancy can be enhanced by sequon engineering
- Enhanced glycan occupancy should reduce the induction of these antibodies

Authors

Ronald Derking, Joel D. Allen, Christopher A. Cottrell, ..., Andrew B. Ward, Max Crispin, Rogier W. Sanders

Correspondence

max.crispin@soton.ac.uk (M.C.), r.w.sanders@amsterdamumc.nl (R.W.S.)

In brief

Derking et al. present a strategy to enhance glycan occupancy on recombinant HIV-1 envelope trimers to mimic that of the virus. This strategy eliminates artificial glycan holes on these trimers that induce non-neutralizing antibodies. The induction of these antibodies could be distractive for immuno-focusing strategies.

Derking et al., 2021, Cell Reports *35*, 108933
April 6, 2021 © 2021 The Author(s).
https://doi.org/10.1016/j.celrep.2021.108933

CellPress

Cell Reports

CellPress OPEN ACCESS

Article

Enhancing glycan occupancy of soluble HIV-1 envelope trimers to mimic the native viral spike

Ronald Derking,[1,7] Joel D. Allen,[2,7] Christopher A. Cottrell,[3] Kwinten Sliepen,[1] Gemma E. Seabright,[2,4] Wen-Hsin Lee,[3] Yoann Aldon,[1] Kimmo Rantalainen,[3] Aleksandar Antanasijevic,[3] Jeffrey Copps,[3] Anila Yasmeen,[5] Albert Cupo,[5] Victor M. Cruz Portillo,[5] Meliawati Poniman,[1] Niki Bol,[1] Patricia van der Woude,[1] Steven W. de Taeye,[1] Tom L.G.M. van den Kerkhof,[1] P.J. Klasse,[5] Gabriel Ozorowski,[3,6] Marit J. van Gils,[1] John P. Moore,[5] Andrew B. Ward,[3,6] Max Crispin,[2,*] and Rogier W. Sanders[1,5,8,*]

[1]Department of Medical Microbiology, Amsterdam Infection and Immunity Institute, Amsterdam UMC, University of Amsterdam, Amsterdam 1105 AZ, the Netherlands

[2]School of Biological Sciences, University of Southampton, Southampton SO17 1BJ, UK

[3]Department of Integrative Structural and Computational Biology, The Scripps Research Institute, La Jolla, CA 92037, USA

[4]Oxford Glycobiology Institute, Department of Biochemistry, University of Oxford, Oxford OX1 3QU, UK

[5]Department of Microbiology and Immunology, Weill Cornell Medical College, Cornell University, New York, NY, USA

[6]Center for HIV/AIDS Vaccine Development, IAVI Neutralizing Antibody Center and the Collaboration for AIDS Vaccine Discovery (CAVD), The Scripps Research Institute, La Jolla, CA 92037, USA

[7]These authors contributed equally

[8]Lead contact

*Correspondence: max.crispin@soton.ac.uk (M.C.), r.w.sanders@amsterdamumc.nl (R.W.S.)

https://doi.org/10.1016/j.celrep.2021.108933

SUMMARY

Artificial glycan holes on recombinant Env-based vaccines occur when a potential N-linked glycosylation site (PNGS) is under-occupied, but not on their viral counterparts. Native-like SOSIP trimers, including clinical candidates, contain such holes in the glycan shield that induce strain-specific neutralizing antibodies (NAbs) or non-NAbs. To eliminate glycan holes and mimic the glycosylation of native BG505 Env, we replace all 12 NxS sequons on BG505 SOSIP with NxT. All PNGS, except N133 and N160, are nearly fully occupied. Occupancy of the N133 site is increased by changing N133 to NxS, whereas occupancy of the N160 site is restored by reverting the nearby N156 sequon to NxS. Hence, PNGS in close proximity, such as in the N133-N137 and N156-N160 pairs, affect each other's occupancy. We further apply this approach to improve the occupancy of several Env strains. Increasing glycan occupancy should reduce off-target immune responses to vaccine antigens.

INTRODUCTION

The HIV-1 envelope glycoprotein spike (Env) initiates viral entry in host cells and is the only target for neutralizing antibodies (NAbs) (Gelderblom et al., 1987; Wei et al., 2003). Env is a trimer of heterodimers consisting of gp120 and gp41 subunits. During the early stages of protein synthesis, Env is decorated by *N*-linked glycans which, upon leaving the Golgi apparatus, comprise ~50% of the total mass (Doms et al., 1993; Earl et al., 1991; Leonard et al., 1990). The *N*-linked glycans play a critical role in the viral life cycle, including Env folding, binding to lectin receptors, and immune evasion by shielding underlying protein epitopes (Crispin et al., 2018). The gp120 subunit contains up to 35 potential *N*-linked glycosylation sites (PNGS) and gp41 contains typically 4 PNGS. Hence, there can be as many as 120 PNGS per trimer. Most of these PNGS are attachment points for *N*-linked glycans by the host cell glycosylation machinery, but some may remain unoccupied (Allan et al., 1985; Struwe et al., 2018).

N-linked glycans are attached to asparagine residues (N) located in NxT/S sequons where x can be any amino acid except proline. The amino acid that follows the NxT/S sequon can also affect glycan attachment: a proline at this position abrogates glycosylation (Mellquist et al., 1998). The enzyme responsible for glycan attachment is oligosaccharyltransferase (OST), which consists of seven to eight non-identical subunits. The catalytic subunit has two isoforms STT3A and STT3B (Ruiz-Canada et al., 2009; Shrimal and Gilmore, 2013). The STT3A subunit is associated with the translocon and attaches glycans during translation, but it sometimes skips sequons that are in close proximity, i.e., NxT/S-NxT/S or NxT/S-x-NxT/S motifs (termed gap-0 and gap-1 sites, respectively). The STT3B subunit facilitates co-translational and post-translational glycosylation of sequons that are skipped by STT3A. Even so, not all gap-0 and gap-1 sites are fully occupied by glycans (Shrimal and Gilmore, 2013). HIV-1 Env contains a number of gap-0 and gap-1 sites, i.e., the N156/N160 gap-1 site that contains glycans that are essential for several broadly neutralizing antibodies (bNAb) (Los Alamos database; https://www.hiv.lanl.gov) (Julien et al., 2013a; Lee et al., 2017; Shrimal and Gilmore, 2013; Sok et al., 2014).

This is an open access article under the CC BY license (http://creativecommons.org/licenses/by/4.0/).

The protein surface of Env is extensively shielded by glycans, contributing to its poor immunogenicity (Klasse et al., 2020). Env and Env-based vaccines, including native-like SOSIP trimers, have holes in their glycan shield from two different sources.

First, a conserved PNGS can be absent from Env of a particular isolate; for example, the conserved glycosylation sequons at N241 and N289 are not present in the BG505 virus and SOSIP trimers. The absence of these two glycans creates an immunodominant hole in the glycan shield that is responsible the majority of strain-specific NAb responses induced by BG505 SOSIP trimers in animals (McCoy et al., 2016; Sanders et al., 2015). Of note is that HIV-1 isolates with an intact glycan shield are more capable of inducing broader neutralizing responses than ones with multiple holes, which may act as immunological decoys and impede the development of neutralization breadth (Wagh et al., 2018). Filling glycan holes may be a strategy to focus the immune response on more desirable targets; it can be achieved by restoring the missing PNGS (Klasse et al., 2018; Ringe et al., 2019). Conversely, removing PNGS is a proven strategy to attract antibody responses to the region of choice (Duan et al., 2018; Escolano et al., 2019; Ringe et al., 2019; Zhou et al., 2017).

Second, a PNGS that is in a protein sequence can be under-occupied, probably because the site is often skipped by STT3A and STT3B (Cherepanova et al., 2016; Ruiz-Canada et al., 2009; Shrimal and Gilmore, 2013). Several PNGS in the V1/V2 domain of BG505 SOSIP.664 trimers are under-occupied, creating artificial glycan holes (Struwe et al., 2018). The PNGS at position 611 of these trimers is also under-occupied, due to the existence of elicited antibodies that can only neutralize viruses lacking N611 (Cottrell et al., 2020; McCoy et al., 2016; Sanders et al., 2015). Direct comparisons revealed that glycan occupancy is lower on BG505 SOSIP trimers than on the sequence-matched viral Env (Cao et al., 2018; Struwe et al., 2018). However, increasing the occupancy of a PNGS is not straightforward because the factors that determine whether a PNGS becomes a substrate for OST are poorly understood. Studies on model glycoproteins have, however, shown that NxT sequons have a 2- to 3-fold higher probability of becoming glycosylated compared to NxS (Kasturi et al., 1995), likely because OST has a higher affinity for NxT (Bause, 1984; Gerber et al., 2013; Struwe et al., 2018). In this study, we sought to increase PNGS occupancy on BG505 SOSIP trimers to mimic viral BG505 Env, and thereby eliminate artificial glycan holes. This should facilitate immuno-focusing to more relevant epitopes.

RESULTS

Cryoelectron microscopy reveals N611 under-occupancy

The glycan compositions of native-like HIV-1 Env trimers have been characterized previously (Behrens et al., 2016; Bonomelli et al., 2011; Doores et al., 2010; Go et al., 2015; Pritchard et al., 2015a, 2015b, 2015c). PNGS occupancy analyses of soluble trimers and their viral counterparts are less common, but new techniques have enabled such studies on BG505 SOSIP.664 and other trimers (Cao et al., 2017, 2018; Guttman et al., 2014; Struwe et al., 2018). Here, we performed cryoelectron microscopy (cryo-EM), PNGS occupancy, and glycan composition analyses on BG505 SOSIP.v4.1 trimers (from here on referred to as wild-type [WT] proteins) (de Taeye et al., 2015). Monoclonal antibodies (mAbs) have been isolated from rabbits and rhesus macaques that were immunized with BG505 SOSIP.664 trimers by single B cell sorting and B cell receptor (BCR) cloning (Cottrell et al., 2020; McCoy et al., 2016; Sanders et al., 2015). Although many of the MAbs targeted the N241/N289 glycan hole, a subset of MAbs was directed against the N611 glycan hole. The implications of these findings are that the region underneath the N611 glycan on BG505 SOSIP trimers is immunogenic when the N611 PNGS is under-occupied, and the same site is more extensively occupied on the virus.

To probe the N611 under-occupancy of BG505 SOSIP, we used mAb RM20E1, directed to the N611 site that was isolated from a BG505 SOSIP.664-immunized macaque, and performed cryo-EM with the Fab in complex with the BG505 SOSIP.v5.2 trimer (Cottrell et al., 2020; Sanders et al., 2015; Torrents de la Peña et al., 2017). Multiple rounds of 3D sorting were performed to segregate trimers with zero, one, two, or three RM20E1 Fabs attached (Figure 1A). Density corresponding to the N611 glycan was observed on all protomers with no Fab attached, however, in contrast, there was no N611 density on any protomers bound to RM20E1 (Figure 1B). These observations are consistent with the previously described binding and neutralization results (Cottrell et al., 2020; McCoy et al., 2016). A further analysis of the numbers of trimers in 3D-sorted classes with zero, one, two, or three RM20E1 Fabs showed that N611 was occupied on ~40% of the protomers.

Several PNGS on BG505 SOSIP.v4.1 trimers are under-occupied

Next, we used several techniques to gain a more detailed understanding of the glycan shield on the WT protein. The D7324-tagged WT protein (Figure 1C) was expressed in HEK293F cells and purified by affinity chromatography using the PGT145 bNAb, which is selective for native-like trimers (Pugach et al., 2015). We also affinity purified the D7324-tagged WT protein using the 2G12 bNAb followed by size exclusion chromatography (SEC) (Sanders et al., 2013; de Taeye et al., 2015). Both methods are routinely used for purifying native-like trimers, including in cGMP programs (Dey et al., 2018; Pugach et al., 2015). WT proteins purified via the PGT145 and 2G12/SEC methods had a homogeneous native-like conformation as judged by negative stain electron microscopy (NS-EM) (Figures 1D and S1A). Overall, analyses of the PGT145- and 2G12/SEC-purified proteins yielded very similar data, with a few notable exceptions (see below). We present data on the PGT145-purified proteins in Figures 1, 2, 3, and 4, with the Figures S1, S2, S5, and S6 for their 2G12/SEC-purified counterparts in the Supplemental information.

To determine the total oligomannose content of the purified WT protein, we performed hydrophilic interaction liquid chromatography-ultra performance liquid chromatography (HILIC-UPLC) (Struwe et al., 2018). The PGT145-purified WT protein contained 56% oligomannose-type glycans and 60% oligomannose-type glycans when purified using 2G12/SEC. In both cases, $Man_8GlcNAc_2$ and $Man_9GlcNAc_2$ were the predominant glycan species (Figures 1E and S1B), consistent with previous

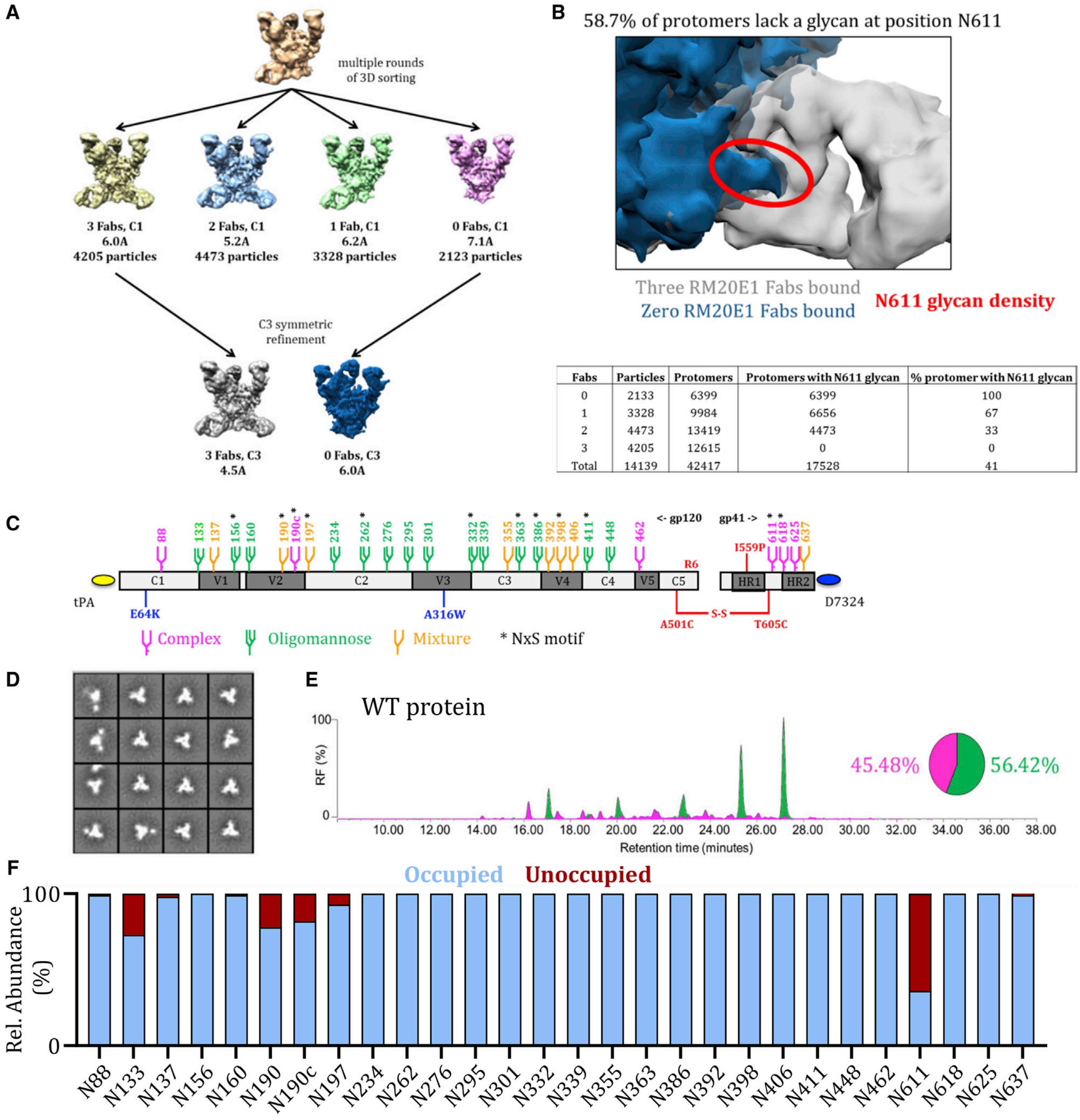

Fabs	Particles	Protomers	Protomers with N611 glycan	% protomer with N611 glycan
0	2133	6399	6399	100
1	3328	9984	6656	67
2	4473	13419	4473	33
3	4205	12615	0	0
Total	14139	42417	17528	41

Figure 1. Several PNGS on BG505 SOSIP.v4.1 trimers are under-occupied

(A) Cryo-EM analysis of WT proteins in complex with the RM20E1 Fab. Complexes were sorted based on the number of Fabs bound; the numbers of particles in each reconstruction are listed, as are the resolutions of the final reconstructions.

(B) Overlay of the Cryo-EM reconstructions of the BG505 SOSIP.664 trimer alone (in blue) and in complex with the RM20E1 Fab (in gray). The density of the N611 glycan (also in blue) on the trimer without RM20E1 is highlighted in red to illustrate clashes with the RM20E1 Fab (in gray). The total number of complexes with different numbers of Fab bound is indicated in the table, as are the protomers with N611 glycan density. From these numbers, the % of protomers that lack the N611 glycan could be calculated (~59%).

(C) Linear representation of the D7324-tagged BG505 SOSIP.v4.1 trimer. The SOSIP changes and the stabilizing E64K and A316W mutations are all highlighted (Sanders et al., 2013; de Taeye et al., 2015). The glycans are also indicated, with the amino acid numbering based on the HXB2 strain. Sites that are predominantly occupied by oligomannose species are colored green and by complex species in magenta, whereas orange indicates that either an oligomannose or a complex glycoform can be present, based on data presented in (D). An asterisk indicates that the PNGS is encoded by an NxS sequon.

(D) NS-EM analysis of WT proteins, showing the 2D class-averages.

(legend continued on next page)

Table 1. Occurrence of NxT and NxS sequons in the BG505 sequence compared to 6,516 unique Env sequences found in the Los Alamos database

			Frequency in natural isolates[a]			Glycan occupancy (%)
Glycan	BG505	Motif	% NxT	% NxS	% no PNGS	
N88	NxT	NVT	98.2	0.4	1.4	>95
N133	NxT	NVT	15.0	3.0	82.0	75
N137	NxT	NIT	20.1	3.5	76.4	>95
N156*	NxS*	NCS*	8.6*	87.6*	3.8*	>95*
N160	NxT	NMT	88.9	2.1	9.0	>95
N190*	NxS*	NRS*	ND*,[b]	ND*,[b]	ND*,[b]	80*
N190c*	NxS*	NNS*	ND*,[b]	ND*,[b]	ND*,[b]	75*
N197*	NxS*	NTS*	1.1*	96.8*	2.1*	90*
N234	NxT	NGT	74.8	5.0	20.2	>95
N262*	NxS*	NGS*	0.2*	98.9*	0.9*	>95*
N276	NxT	NIT	76.5	18.5	5.1	>95
N295	NxT	NCT	58.2	1.5	40.4	>95
N301	NxT	NNT	93.7	0.2	6.2	>95
N332*	NxS*	NVS*	3.0*	68.5*	28.5*	>95*
N339	NxT	NET	65.1	0.1	34.8	>95
N355	NxT	NNT	72.8	1.6	25.5	>95
N363*	NxS*	NSS*	0.4*	7.7	91.9*	>95*
N386*	NxS*	NTS*	40.0*	46.8*	13.2*	>95*
N392	NxT	NST	70.0	9.2	20.8	>95
N398*	NxS*	NTS*	11.0*	2.9*	86.1*	>95*
N406	NxT	NST	2.7	0.5	96.8	>95
N411*	NxS*	NDS*	44.4*	1.3*	54.3*	>95*
N448	NxT	NIT	87.4	0.1	12.5	>95
N463	NxT	NST	ND[b]	ND[b]	ND[b]	>95
N611*	NxS*	NSS*	11.6*	86.8*	1.6*	40*
N618*	NxS*	NLS*	16.6*	75.3*	8.1*	>95*
N625	NxT	NMT	96.2	0.0	3.8	>95
N637	NxT	NYT	92.5	3.8	3.7	>95

The occupancy of each PNGS on the BG505 SOSIP.v4.1 trimer is also recorded. *Indicates the PNGS that have an NxS motif.
[a]Data obtained from 6,516 unique HIV envelope sequences found in the Los Alamos database (https://www.hiv.lanl.gov).
[b]PNGS are located in highly variable regions and the specific sites differ between the natural isolates, making reliable calculations not possible.

reports on native-like SOSIP trimers (Behrens et al., 2016; Struwe et al., 2018; de Taeye et al., 2015).

To investigate PNGS occupancy and glycan composition, we performed site-specific analysis of the WT protein using liquid chromatography-electrospray ionization (LC-ESI) mass spectrometry (MS) with an Orbitrap Fusion mass spectrometer (Struwe et al., 2018; Figure 1F). PNGS occupancy is expressed as the percentage of the total peptide that is modified by a glycan; we considered a modification level of ≥95% as indicative of full occupancy. The majority of PNGS on gp120 from the PGT145-purified WT protein were fully occupied, including the five located within a 25-amino acid stretch of V4 (N386, N392, N398, N406, and N411). However, under-occupancy was seen at sites near the apex: N133, ~75% occupancy; N190, ~80%; N190c, ~80%; and N197, ~93% (Figure 1F). The sites under-occupied on the 2G12/SEC-purified WT protein were: N133, ~90% occupancy; N137, ~80%; N190, ~90%; and N190c, ~80% (Figure S1C). These regions of under-occupancy are similar to those described previously for BG505 SOSIP.664 trimers (Struwe et al., 2018).

In our previous study, we did not perform a site-specific PNGS occupancy analysis for gp41 (Struwe et al., 2018). We now report that the N611 glycan on the PGT145-purified protein is markedly under-occupied (~35% occupancy) (Figures 1F and S1C). This finding is highly concordant with the cryo-EM analysis (Figure 1A). The other three gp41 sites (N618, N625, and N637) were fully occupied on the PGT145-purified WT protein. However, the N625 site was less occupied (~70%) and the N611 site more occupied (70%) on the 2G12/SEC-purified WT protein (Figure S1C). For most PNGS on the WT protein, the glycoforms were very similar to those observed in our previous studies (Behrens et al., 2016; Struwe et al., 2018; Figure S1C).

Glycan occupancy is enhanced by PNGS sequon engineering

Although the determinants of glycan occupancy are not clear, studies on model glycoproteins have shown that the NxT sequon has a higher probability of becoming glycosylated compared to an NxS sequon (Kasturi et al., 1995). The use of NxS (~40%) versus NxT (~60%) in the BG505 sequence is consistent with most isolates in the Los Alamos database (Table 1). Thus, when an NxS sequon is present at a particular site, it is also the case for BG505. To increase glycan occupancy, we first designed two stabilized BG505 SOSIP.v4.1 trimers (de Taeye et al., 2015) in which we changed all the PNGS to either NxT or NxS. The two variants are designated NxT protein and NxS protein, respectively.

We used SDS-PAGE and Blue Native (BN-) PAGE to assess the expression and trimer formation of unpurified WT, NxT, and NxS proteins produced in transfected HEK293T cells (Figures S1D and S1E). The NxT protein was expressed efficiently and formed trimers, although slightly less well than the WT protein, but the NxS protein was not expressed efficiently. A D7324-capture ELISA using bNAbs 2G12, VRC01, PGT145, and PGT151 to probe the folding of the proteins in the unpurified supernatants

(E) HILIC-UPLC analysis of WT proteins. Peaks colored green represent glycans that can be cleaved by endoglycosidase H (endoH) and correspond to oligomannose or hybrid-type glycans. The HILIC-UPLC spectra and pie chart show the overall oligomannose glycan (green) and complex/hybrid glycan (magenta) content.
(F) Quantification of site-specific occupancy for all 28 PNGS on WT proteins, derived from LC-ESI MS analyses. Results are the mean of two independent biological replicates of the WT protein. The data displayed represents the relative proportion of occupied (blue) versus unoccupied (red). All the data in (A)–(F) were derived using WT proteins produced in HEK293F cells followed by PGT145-affinity purification. The corresponding data on WT proteins purified by the 2G12/SEC method are in Figure S1.

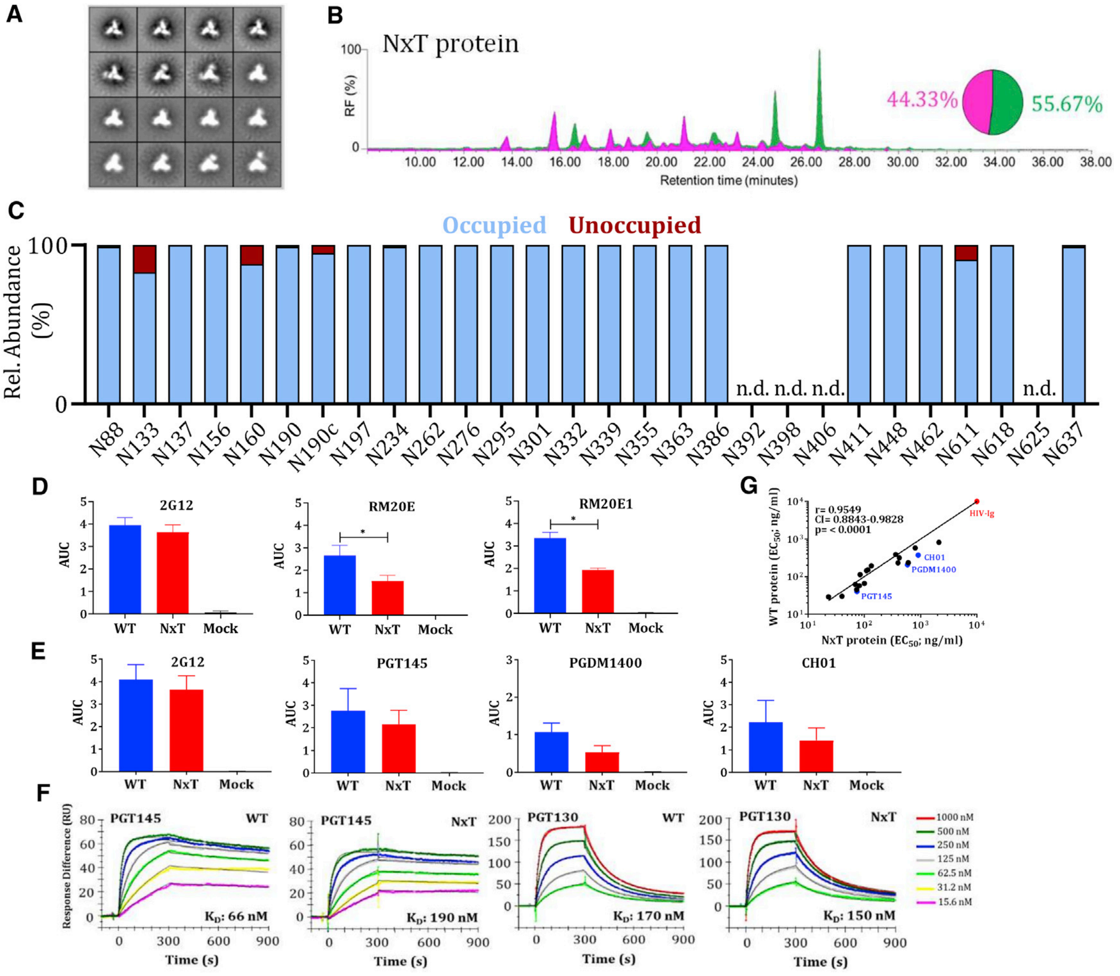

Figure 2. Glycan occupancy is increased by PNGS sequon engineering

(A) NS-EM analysis of NxT proteins showing the 2D class-averages.

(B) HILIC-UPLC analysis of the NxT protein. The color coding of the spectra and pie chart is the same as in Figure 1E.

(C) Quantification of site-specific occupancy for all 28 PNGS on NxT proteins, derived from LC-ESI MS analyses.

(D) Binding of WT and NxT proteins to two N611-directed non-NAbs, RM20E and RM20E1, isolated from BG505 SOSIP.664 immunized rhesus macaques. The area under the curve (AUC) values derived from ELISA titration curves are plotted. *Indicates a significant difference ($p < 0.05$) between the WT and NxT proteins, calculated using a Mann-Whitney 2-tailed test.

(E) Binding of WT and NxT proteins to three V2-apex directed bNAbs, PGT145, PGDM1400, and CH01, and also 2G12 for comparison. AUC values derived from derived from ELISA titration curves are plotted.

(F) Binding of bNAbs PGT145 and PGT130 to WT and NxT proteins, assessed by SPR. A summary of the binding kinetics for both bNAbs is in Figure S2E.

(G) The EC_{50} values derived using WT and NxT proteins were plotted and compared using Spearman's correlation coefficient. All analyses were performed on NxT proteins produced in HEK293F followed by PGT145 purification. The corresponding data on NxT proteins purified by the 2G12/SEC method are in Figure S2.

confirmed these findings (Figure S1F). Thus, all four bNAbs bound to the NxT and WT proteins, but the NxS protein could only be detected using 2G12. The defect in NxS protein expression might be caused by the absence of glycans important for Env to fold properly in the ER (Li et al., 1993).

Purified, HEK293F cell-expressed NxT proteins were fully cleaved with no evidence of aggregation or dissociation (Figure S1G). Compared to WT, the NxT protein migrated more slowly through the SDS- and BN-PAGE gels, indicating more extensive glycosylation. The NS-EM 2D-class averages showed that the NxT protein was in a native-like conformation (Figures 2A and S2A).

The overall glycan composition of the NxT protein was similar to WT, with oligomannose contents of 56% and 60% after PGT145 and 2G12/SEC-purification, respectively (Figures 2B and S2B). However, the LC-ESI MS experiments revealed some striking differences in PNGS occupancy. Whereas the N190, N190c, and N197 PNGS were under-occupied on the WT protein (Figure 1F), the same sites were >95% occupied on both preparations of the NxT proteins (Figures 2C and S2C). Furthermore, occupancy of N611 on gp41 increased from ~40% and ~70% on the PGT145- and 2G12/SEC-purified WT proteins, respectively, to ~90% and ~85% for the corresponding NxT proteins (Figures 2C and S2C). Occupancy of some sites

including N392, N398, N406, and N625 could not be determined. These sites tend to be in regions that are densely glycosylated and ionize poorly, resulting in glycopeptides of insufficient quality. We and others encountered similar problems previously (Cao et al., 2018; Struwe et al., 2018).

Occupancy at N133 was not restored on the PGT145-purified NxT protein as it increased from 73% to 83%, which means that ~1 in 5 protomers still lack a glycan at this position. Conversely, N133 occupancy decreased to ~60% on the 2G12/SEC-purified NxT protein, compared to ~95% for WT (Figure S2C). Furthermore, although the N160 PNGS was fully occupied on the PGT145-purified WT protein, occupancy decreased to ~85% for the NxT protein (Figure 2C). An even greater decrease of 30 percentage points was observed for the 2G12/SEC-purified NxT protein (Figure S2C). The PGT145 epitope requires at least two occupied N160 sites, which probably explains the higher N160 occupancy on the PGT145- versus 2G12/SEC-purified NxT protein (Lee et al., 2017).

There were no substantial differences when the glycoforms at each PNGS of the NxT protein were compared to the WT protein. The sites that were more occupied on the NxT protein than on WT (N190, N190c, N197, and N611) were all predominantly occupied by complex glycans (Figure S2C).

The increased occupancy of N611 on NxT proteins could reduce the immunogenicity of non-NAb epitopes associated with this artificial glycan hole. To assess this *in vitro*, we used two N611-directed non-NAbs RM20E and RM20E1. Using a D7324-capture ELISA, the PGT145-purified NxT protein was significantly less reactive than the WT protein with non-NAbs RM20E ($p = 0.03$) and RM20E1 ($p = 0.03$), calculated using a two-tailed Mann-Whitney test (Figure 2D).

The antigenicity of the PGT145-purified NxT proteins was assessed by ELISA (Figure S3). Most of the bNAbs tested recognize epitopes involving glycans; they include ones directed against quaternary-structure dependent V1/V2-apex sites (PG9, PG16, PGT145, PGDM1400, and CH01), the N332-glycan dependent epitope cluster (PGT121-123, PGT125-128, PGT130, 2G12, and PGT135), the quaternary-structure dependent epitopes at the gp120-g41 interface (PGT151 and 35O22), and gp41 (3BC315). We also used bNAbs against the CD4bs (VRC01 and 3BNC60) and polyclonal HIV immunoglobulin (HIV-Ig). In all cases, the bNAbs bound similarly to the WT and NxT proteins, with <3-fold difference in 50% binding concentrations (EC_{50}) (Figure S3). An area under the curve (AUC) analysis, however, revealed that bNAbs against the trimer apex were less reactive with the NxT protein (Figure 2E). This finding is concordant the reduced occupancy of N160 on this trimer, as the epitopes involve this glycan. Similar results were obtained when the 2G12/SEC-purified WT and NxT proteins was tested by ELISA (Figure S2D), and by surface plasmon resonance (SPR) (WT, K_D 66 nM versus NxT, K_D 190 nM for PGT145; the K_D values are for the initial interaction as fitted with a conformational-change model) (Yasmeen et al., 2014) (Figures 2F and S2E). The EC_{50} values for bNAb binding to the WT and NxT proteins were highly correlated (Spearman $r = 0.95$, $p < 0.0001$ [Figure 2G] and Spearman $r = 0.97$, $p < 0.0001$ [Figure S2F]).

To verify whether NxT sequon engineering was compatible with Env function, we generated a BG505.T332N infectious molecular clone (IMC) in which we changed all Env PNGS to NxT. The NxT and WT viruses were equally infectious (Figure S4A). In contrast, an IMC with the PNGS all changed to NxS was not infectious, which is consistent with the poor expression of the corresponding NxS protein (Figures S1 and S4A). In a neutralization assay, the NxT virus had reduced sensitivity to PGT145 (26-fold increase in IC_{50}) (Figures S4C and S4D). A second V1/V2-apex bNAb, CH01, neutralized the NxT and WT viruses comparably, as judged by IC_{50} values, but the maximum extent of neutralization was only ~60% for the NxT virus whereas its WT counterpart was completely neutralized at high CH01 concentrations (Figure S4). The impact of the NxT modification on the V1/V2-apex is therefore seen with viruses and recombinant trimers and is consistent with under-occupancy of the PNGS at N160 in both contexts.

Occupancy of gap-1 sites can be enhanced by reducing OST affinity of the first site

The reduced N160 occupancy on the NxT protein was surprising because the glycan sequon was unchanged compared to WT. However, the preceding N156 sequon was changed from NxS to NxT. This site is fully occupied on WT proteins purified by both methods (Figures 1F and S1C). We hypothesized that increasing the affinity of the PNGS at N156 for OST by changing it to NxT might interfere with glycan attachment at N160. We therefore reverted T158 to S on the NxT protein. The purified NxT T158S protein adopted a native-like conformation (Figures 3A and S5A) and had an overall oligomannose content of 50% and 48% for PGT145- and 2G12/SEC-purified proteins, respectively (Figures 3B and S5B). The T158S mutation enhanced N160 occupancy to >95% for both preparations of purified proteins (Figures 3C and S5C), similar to N160 on the WT protein (Figures 1D and S1C).

The only site that remained considerably under-occupied on the NxT T158S protein was N133 (~75% occupancy) (Figure 3C). Based on our success with the N160 site and noting that the N133 and N137 sequons are spaced similarly to N156 and N160, we adopted the same strategy to increase N133 occupancy. Accordingly, we changed residue 135 from T to S in the background of the NxT T158S protein. Occupancy of the N133 site increased from ~75% for the NxT T158S protein (Figure 3C) to ~95% on NxT T135S T158S (Figures 3D, 3E, S6A, and S6B). Every PNGS was now >95% occupied on the NxT T135S T158S protein (Figure 3D). However, for 2G12/SEC-purified NxT T135S T158S, N133 and N190 occupancy remained lower (~65% and 86%, respectively) (Figure S6B). Hence, the affinity purification method can impact PNGS occupancy, but the glycan composition remains similar using different purification methods (Pritchard et al., 2015b).

The glycoforms at each site on the PGT145-purified NxT T135S T158S and WT proteins were very similar, but with a few exceptions. The N88, N190, and N406 glycans were more processed and closer resemble what is found on viral Env (Figure S6B; Struwe et al., 2018). Conversely, the N133 glycan was less processed on this protein than WT. The glycans at N137, N276, and N406 were also more processed on the 2G12/SEC-purified NxT T135S T158S protein (Figure S6B). The increased processing at N276 is similar to what was seen

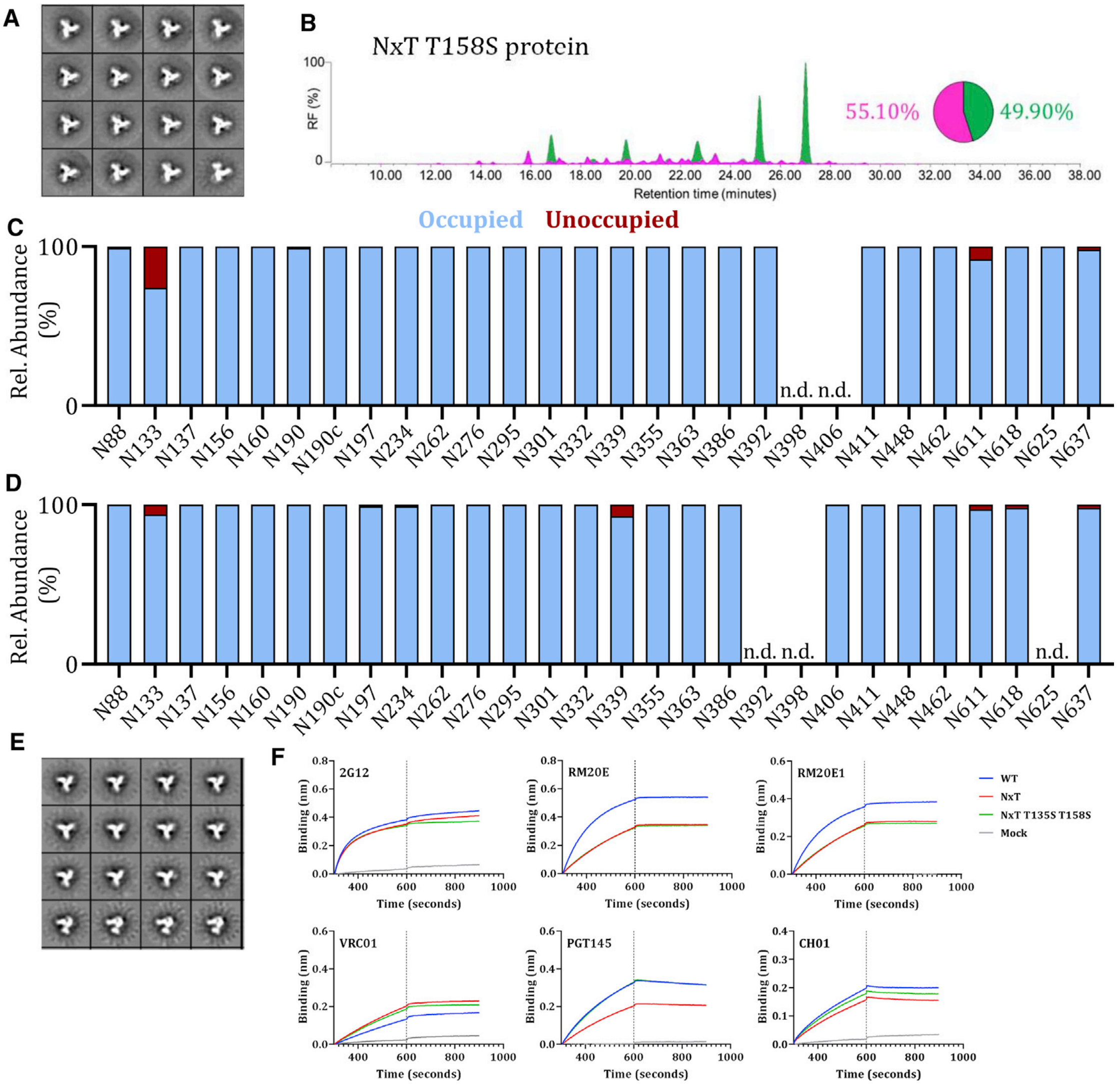

Figure 3. Occupancy of gap-1 sites can be increased by reducing the affinity of the first site for OST

(A) NS-EM analysis of NxT T158S proteins, showing the 2D class-averages.

(B) HILIC-UPLC analysis of the NxT T158S protein. The color coding of the spectra and pie chart is the same as in Figure 1E.

(C) Quantification of glycan occupancy using LC-ESI MS of the 28 PNGS on NxT T158S proteins.

(D) Quantification of site-specific occupancy for all 28 PNGS on NxT T135S T158S proteins, derived from LC-ESI MS analyses. Results shown are the mean of two independent biological replicates of the NxT T135S T158S protein. The corresponding data on NxT T135S T158S proteins purified by the 2G12/SEC method are in Figure S6.

(E) NS-EM analysis of NxT T135S T158S proteins, showing the 2D class-averages.

(F) Binding of non-NAbs RM20E and RM20E1 to WT, NxT, and NxT T135S T158S proteins, assessed by BLI. The bNAb 2G12 was also tested, for comparison. We also tested the binding of bNAbs VRC01, PGT145, and CH01. The average binding curves from 3 independent duplicates are shown. All analyses were performed on NxT T135S T158S trimers produced in HEK293F cells and affinity purified using PGT145.

with the similarly purified NxT T158S protein but differs from their PGT145-purified counterparts.

In a biolayer interferometry (BLI) assay, MAbs RM20E and RM20E1 were less reactive with the PGT145-purified NxT T135S T158S protein than WT, which is further evidence that N611 is more occupied on the modified trimer (Figure 3F). BLI also showed that the apex-targeting bNAbs PGT145 and CH01 were equally reactive with the WT and NxT T135S T158S proteins (Figure 3F). Thus, increasing N160 occupancy via the T158S mutation improved the presentation of bNAb epitopes at the trimer apex.

In summary, the PGT145-purified BG505 NxT T135S T158S trimer closely mimics viral BG505 Env in respect of overall PNGS occupancy (Figure 4). In particular, the artificial glycan hole recognized by non-NAbs and caused by under-occupancy of the N611 site is now closed (Cao et al., 2018; Struwe et al., 2018), which should facilitate immune-focusing to more relevant neutralizing epitopes.

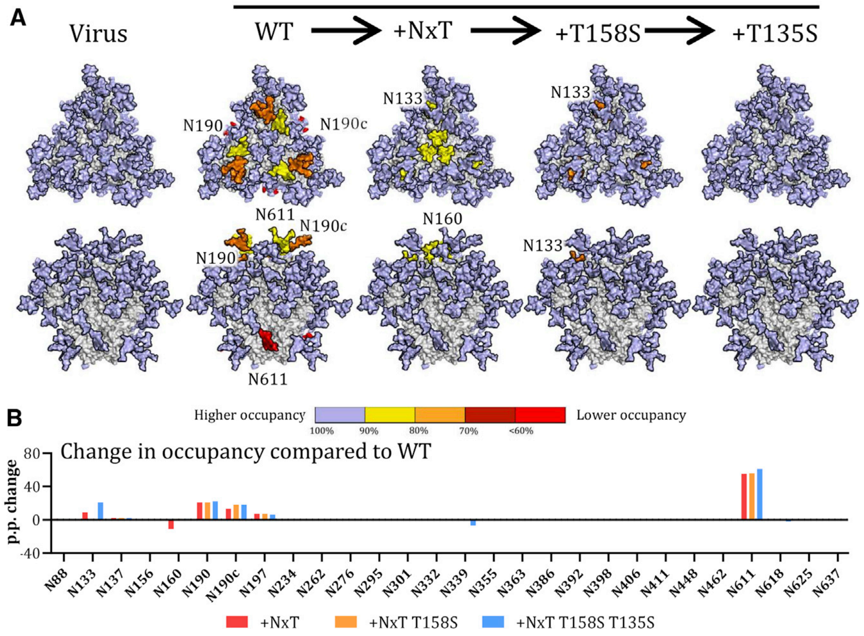

Figure 4. Sequential improvement of PNGS occupancy on SOSIP trimers

(A) Occupancy of each PNGS on the sequentially improved SOSIP.v4.1 trimers (WT, NxT, NxT T158S, and NxT T135S T158S) is compared with the same site on the BG505 virus. Glycans are modeled onto each PNGS on a 3D model of the WT protein. Color coding reflects the occupancy of each PNGS: light blue, full occupancy (>95%); yellow, 80%–95% occupancy; orange, 70%–80% occupancy, dark red, 60%–70% occupancy; bright red, <60% occupancy.

(B) The sequential changes in PNGS occupancy at each PNGS are shown by the bars. The data shown are the percentage changes in occupancy caused by the various PNGS sequon changes, compared to the same site on the WT BG505 SOSIP.664 trimer.

PNGS sequon engineering translates to cells from different species

Although the work described above was performed using human HEK293F cells, these cells are not commonly used for clinical grade proteins, although they have been used to produce recombinant human clotting factor VIII for clinical trial usage (Cupo et al., 2019; Dey et al., 2018; Dumont et al., 2016). To investigate the effect of PNGS sequon engineering in cells more relevant for production of clinical grade material, we transiently transfected ExpiCHO-S cells with the WT protein and affinity-purified it using PGT145. LC-ESI MS analyses revealed that the same sites that were underoccupied in HEK293F WT protein were also under-occupied on the ExpiCHO-S WT protein: N133, ~85% occupancy; N190c, ~62%; N197, ~66%; and N611 site on gp41 (~37% occupancy) (Figures 5A, S6C, and S6D). In addition, a number of sites were less occupied on the ExpiCHO-S material compared to the HEK293F PGT145-purified WT protein: N137, ~85%; and N156, ~92%; and most notably N190, ~8%; and N625, 0% occupancy (Figures S6C and S6D). We do note that N137 and N625 were also under-occupied on HEK293F WT produced proteins purified using 2G12/SEC-purification but not to the extent observed with ExpiCHO-S. Glycoforms at each site were consistent between the cell lines with a few exceptions. Increased processing was observed at N197 and N406 whereas N276 and N637 possessed a higher abundance of oligomannose-type glycans on the ExpiCHO-S material.

PNGS occupancy of ExpiCHO-S produced protein was higher on the NxT T135S T158S protein compared to the corresponding WT protein. Occupancy of PNGS N156, N190c, and N197 was restored fully (>95% occupancy), whereas occupancy of N133 and N190 increased to ~90% (Figures 5A, S6C, and S6D). The N137 site remained unchanged and under-occupied (~85% occupancy). On gp41, the occupancy of N611 was restored (>95% occupancy), but N625 remained completely under-occupied (0% occupancy). Overall, the benefit of PNGS sequon engineering for HEK293F-expressed protein was reproduced in ExpiCHO-S cells. However, an important observation is that PNGS under-occupancy was more pronounced on ExpiCHO-S produced protein. Thus, in particular the two sites at N190 and N625 were notably less occupied on ExpiCHO-S material. The under-occupancy of N625, which is present as an NxT sequon in WT and mutant proteins, was not restored in the NxT T135S T158S protein. N625 occupancy can vary between protein preparations and other approaches should be taken to address this.

PNGS sequon engineering enhances PNGS occupancy on SOSIP trimers from diverse isolates

To explore whether PNGS sequon engineering could be applied to Env trimers from other virus isolates, we generated three additional SOSIP trimers containing NxT T158S mutations. Two were based on clade B sequences AMC009 and AMC011 (Euler et al., 2019; van Gils et al., 2016; Schorcht et al., 2020) and the third was based on the HxB2 reference sequence. We transiently expressed WT and NxT T158S proteins in HEK293F cells, followed by PGT145-affinity purification for AMC009 and AMC011 and 2G12/SEC-purification for HxB2 (Figure S7A). We assessed the antigenicity using bNAbs 2G12, VRC01, PGT145, and PGT151 (Figures S7B and S7C). Overall, the bNAbs bound similarly to the WT and NxT T158S proteins. PGT145 did not bind the HxB2 and HxB2 NxT T158S proteins.

LC-ESI MS analyses revealed that on the AMC009 WT protein the following sites were under-occupied: N139, ~15% occupied; N156, ~90%; N197, ~75%; N289, ~10%; N611, ~40%; N616, ~45%; and N637, ~35% (Figures 5B and S7D). PNGS occupancy on the AMC011 WT protein overall was higher at each site, compared to the other proteins, but a few sites were highly under-occupied: N141, ~20% occupied; N241, ~85%; N611, ~10%; and N616, ~5% (Figures 5C and S7D). For the HxB2 WT protein, the following sites were under-occupied: N156, ~55% occupied; N160, ~25%; N189, ~45%; N262, ~90%;

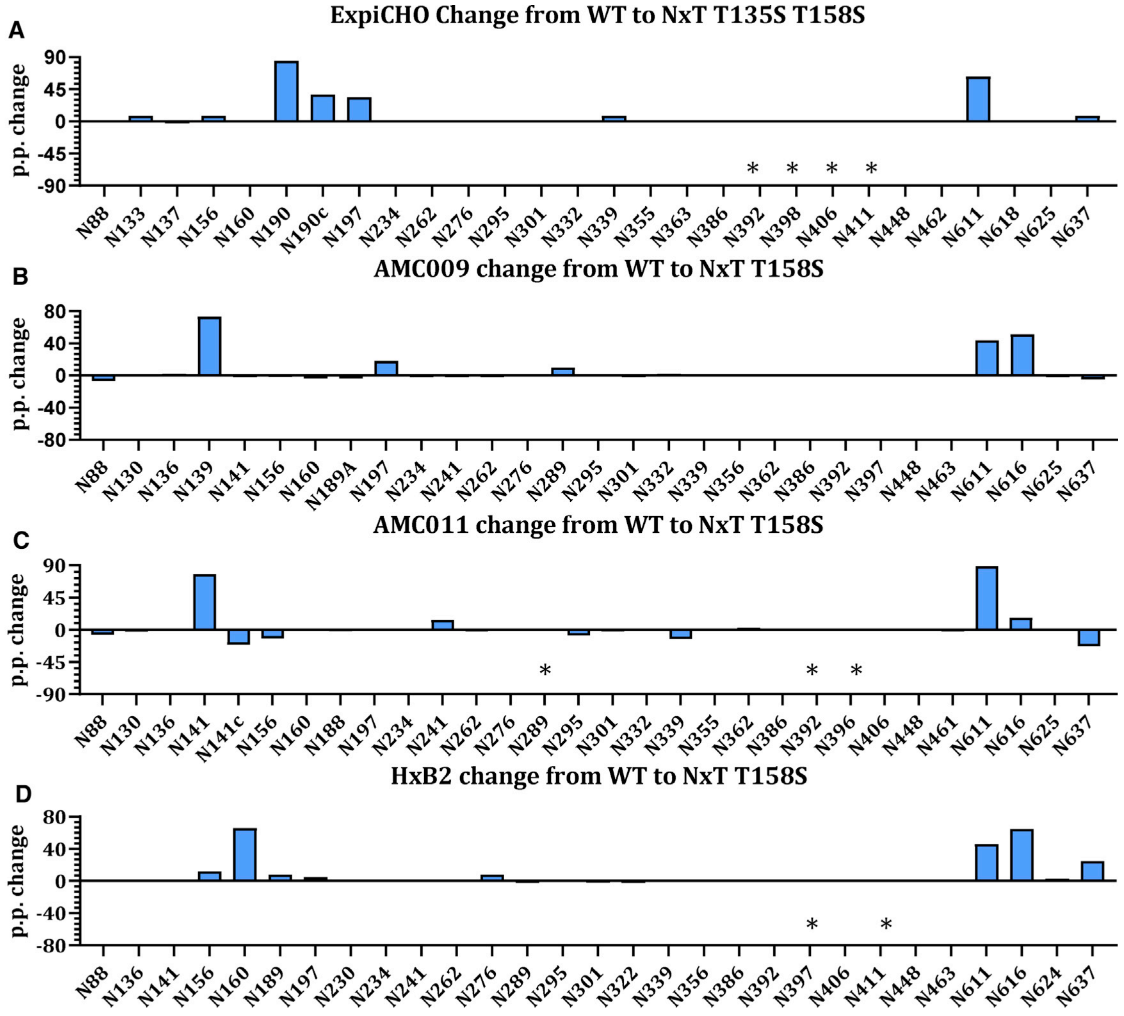

Figure 5. PNGS sequon engineering is applicable to Env trimers produced in cells from different species and derived from diverse HIV-1 strains

(A) Change in PNGS occupancy between WT proteins produced in ExpiCHO-S cells upon introduction of NxT T135 T158S. The presented data represents the arithmetic difference between the glycan occupancy of the NxT protein minus the WT glycan occupancy, representing a percentage point change (p.p.). A positive p.p. change represents a higher occupancy of the NxT variant compared to the WT. Sites labeled with an asterisk represent those for which data could not be obtained for either the WT or the NxT variant.

(B–D) For additional Env sequences the data is displayed in an identical manner for (B) AMC009, (C) AMC011, and (D) HxB2. N411 was introduced into HxB2 NxT but is not present in WT and is marked as an asterisk.

N611, ~45%; N616, ~15%; and N637, ~60% (Figures 5D and S7D). In general, we observed that the PNGS that are most frequently underoccupied are those surrounding the trimer apex and those on gp41. This is very similar as observed for the BG505 WT protein but with isolate-specific differences (Figure 1F).

PNGS occupancy was enhanced substantially on the AMC009, AMC011, and HxB2 NxT T158S proteins (Figures 5 and S7D). On the AMC009 NxT T158S protein, occupancy of all PNGS approached full occupancy, except N637 in gp41 for which occupancy did not change (Figures 5B and S7D). On the AMC011 NxT T158S protein, the most notable changes were seen for N141, N241, and N611 that reached full occupancy (>95% occupancy) (Figures 5C and S7D). Occupancy of N616 on the AMC011 NxT T158S protein increased, from ~10% to ~20% occupied, and PNGS occupancy at N637 decreased, from ~85% to ~60% occupied. On the HxB2 WT protein, the N156 and N160 sequons are both NxS and on the HxB2 NxT T158S protein the occupancy of N156 and N160 increased (to ~70% and ~89% occupancy, respectively) (Figures 5D and S7D). This confirms that the optimal sequon combination at the apex is NxS-x-NxT. Occupancy of N189 was not restored, but occupancy of the N611 and N616 sites on gp41 approached full occupancy. The N637 sequon was also unchanged on the NxT T158S protein, but occupancy also increased for this site (~85% occupancy). We conclude that the NxT modifications

overall present a universal approach to increase PNGS occupancy on Env trimers, however, achieving full PNGS occupancy requires isolate-specific fine-tuning.

DISCUSSION

In this study, we sought to overcome the under-occupancy of PNGS on soluble HIV-1 Env SOSIP trimers from diverse virus isolates and establish guidelines for similar efforts on other vaccine antigens. The absence of a PNGS can be resolved by reintroducing the site (Ringe et al., 2019). Under-occupancy of a PNGS is less easily addressed, but we were able to increase occupancy on these trimers by manipulating the affinity of PNGS sequons for OST. In our final construct, 11 of the 12 NxS sequons were changed to NxT whereas, conversely, 1 of the 16 NxT sequons was switched to NxS. Nearly all of the PNGS were fully occupied on the resulting BG505 SOSIP NxT T135S T158S trimer, which now resembled the viral BG505 trimer in this regard. *In vivo* studies will confirm whether elimination of artificial glycan holes alters immunogenicity. The N611 site appeared to be highly immunogenic for non-NAbs when under-occupied, judged by polyclonal-EM studies performed on sera from animals immunized with BG505 SOSIP.664 (Nogal et al., 2020). The increased N611 occupancy, reduced the binding of the N611-directed non-NAbs. Furthermore, suboptimal glycan occupancy in the V1V2 may result in non-neutralizing antibodies targeting this region (Brouwer et al., 2021). The modified SOSIP trimers should be a suitable design platform for further immuno-focusing efforts intended to facilitate the induction of bNAbs, although additional efforts should be made to close the large glycan hole present at the trimer base (Hu et al., 2015).

PNGS under-occupancy most likely occurs when the catalytic subunits of OST (STT3A and STT3B) skip sites, which occurs relatively frequently when the PNGS are close together (gap-0 or gap-1 sites) (Shrimal and Gilmore, 2013). The WT protein was under-occupied at N133 (gap-1), N190 (gap-0), N190c (gap-0), N197, and N611. In contrast, these same sites are fully occupied on viral Env (Cao et al., 2018; Struwe et al., 2018). Several factors associated with recombinant protein expression may be relevant to the differences. First, viral Env is tethered to the ER membrane via its transmembrane domain that might promote association with OST, particularly for PNGS near the C terminus of the nascent polypeptide. Membrane tethering could also play a role in glycan processing, in particular those close to the C terminus. However, other sites, such as N197, remain less processed on SOSIP trimers (Torrents de la Peña et al., 2019) and need other approaches to resolve these differences. The dwell time in the ER may also be affected resulting in more or less time for OST to act (Cao et al., 2018). Second, the tissue plasminogen activator (TPA) signal peptide used to express the WT protein, which is cleaved off co-translationally, might impact interactions with OST differently than for the WT HIV-1 signal peptide, which is cleaved off post-translationally (Land and Braakman, 2001; Snapp et al., 2017). Third, codon optimization of the recombinant SOSIP trimer may play a role via increased expression levels (Sellhorn et al., 2009). The lower expression of viral Env compared to its recombinant counterpart could cause sequon skipping by OST due to low substrate abundance. Fourth, the use of different producer cell lines (HEK239F or CHO cells for SOSIP trimers, $CD4^+$ T cells for BG505 virus) (Cao et al., 2017, 2018; Go et al., 2017; Struwe et al., 2018) may be relevant because of variation in their levels of OST and other components of the glycoprotein synthesis machinery.

Whatever the reasons for the under-occupancy of sites on the WT protein, we were able to address them. NxT sequons are more efficiently glycosylated than NxS, probably because their affinity for OST is higher. The preference of OST for T over S at the +2 position of PNGS may arise from stronger van der Waals interactions (Bause, 1984; Gerber et al., 2013). Changing all 12 NxS sequons to NxT was compatible with efficient Env expression and trimer formation. When applied to the BG505 virus, infectivity was unaffected, a sensitive indicator that the 12 amino acid substitutions had no major adverse effects. PNGS occupancy on the NxT protein was increased at three sites surrounding the trimer apex, N190, N190c, and N197, to the extent that they were now fully occupied (Figure 4).

In contrast to the increases seen for N190, N190c, N197, and N611, occupancy of N160 on the NxT protein was unexpectedly decreased, even though the N160 sequon itself was unchanged. The reduced occupancy of N160 diminished the binding of several V1/V2 bNAbs to the NxT protein, because their epitopes involve this glycan. Neutralization of the NxT virus by the same bNAbs was also substantially reduced compared to the WT virus.

There appear to be subtle and unpredictable influences on occupancy when PNGS are in close proximity. There are reports that skipping by OST is likely to occur when one or two of the sequons for adjacent PNGS (gap-0 and gap-1) is NxS (Cherepanova et al., 2016; Shrimal and Gilmore, 2013). In contrast, attachment of glycans by OST is efficient when both sequons are NxT. As a result, double NxT sites are highly enriched in libraries of dually glycosylated gap-0 and gap-1 sites (Shrimal and Gilmore, 2013). That finding is consistent with our observation that glycan attachment at the single gap-0 site in BG505 SOSIP trimers (N190 and N190c) was more efficient when these sequons were changed from NxS-NxS in the WT protein to NxT-NxT in its NxT counterpart.

In contrast, the gap-1 sites N156-N160 and N133-N137 on the BG505 SOSIP trimer did not conform to the above pattern. In both cases, occupancy was suboptimal when the two sequons were NxT, but increased in occupancy when the first site was presented as NxS. One explanation is that the x-position of the N156 site is a cysteine involved in a disulfide bond, which could interfere with glycan attachment when OST affinity is increased by the NxT change. For both gap-1 sites a lower OST affinity of one of the sequons, whether driven by a lower on-rate and/or a higher off-rate, may allow a different OST complex to more efficiently associate with the neighboring sequon. This competition for OST between sequons might be more pronounced in glycoproteins with an unusually high glycan density such as HIV-1 Env, compared to more typical human glycoproteins with only a few glycans. Hence, we conclude that increasing glycan occupancy cannot be achieved by simply changing every sequon to NxT; the affinities of sequons for OST and how closely they are spaced are additional factors that need to be considered.

The Env trimer is the basis for HIV-1 vaccine research aimed at inducing broad and protective neutralizing antibodies. A now widely used immunogen design platform that is being explored in preclinical and clinical phase tests is the SOSIP trimer, which can now be based on most viral sequences from various clades (Julien et al., 2015; Klasse et al., 2016, 2018; Kumar et al., 2017; Pugach et al., 2015; Sanders et al., 2013, 2015; Sliepen et al., 2019; de Taeye et al., 2015; Torrents de la Peña et al., 2018). However, SOSIP trimers evaluated to date do not fully mimic viral Env in respect of glycan occupancy; the resulting artificial glycan holes are immunogenic, but they induce antibodies of generally narrow-specificity that are not on the pathway toward neutralization breadth. Here, we show that the NxT T135S T158S protein closely resembles viral Env with respect to both structure and PNGS occupancy. This next generation Env construct, or others based on the principles derived here, will no doubt aid immune-focusing efforts aimed at inducing glycan-dependent bNAbs. Finally, the PNGS sequon-engineering strategies we describe here could be applied to other highly glycosylated vaccine antigens and other biologics where PNGS occupancy could usefully be improved.

STAR★METHODS

Detailed methods are provided in the online version of this paper and include the following:

- KEY RESOURCE TABLE
- RESOURCE AVAILABILITY
  - Lead contact
  - Materials availability
  - Data and code availability
- EXPERIMENTAL MODEL AND SUBJECT DETAILS
  - HEK293T cell culture
  - HEK293F cell culture and transfection
  - ExpiCHO-S cell culture and transfection
- METHOD DETAIL
  - BG505 infectious molecular clones
  - Env SOSIP trimers
  - Antibodies
  - Neutralization assays
  - SDS-PAGE and Blue Native-PAGE
  - Negative stain electron microscopy
  - Cryo electron microscopy
  - Ni-NTA-capture ELISA
  - D7324-capture ELISA
  - Biolayer interferometry (BLI)
  - Surface Plasmon Resonance (SPR)
  - N-glycan analysis using HILIC-UPLC
  - Site-specific glycan analysis using mass spectrometry
  - Site-specific analysis of low abundance N-glycan sites using mass spectrometry
- QUANTIFICATION AND STATISTICAL ANALYSIS

SUPPLEMENTAL INFORMATION

Supplemental information can be found online at https://doi.org/10.1016/j.celrep.2021.108933.

ACKNOWLEDGMENTS

We thank J.E. Robinson, J.R. Mascola, P.D. Kwong, D.R. Burton, B.F. Haynes, M.C. Nussenzweig, and M. Connors for donating antibodies and reagents directly or through the AIDS Reagents Reference Program. This work was supported by the NIH (P0 AI110657 to J.P.M., A.B.W., and R.W.S.) and by the Bill and Melinda Gates Foundation through the Collaboration for AIDS Vaccine Discovery (CAVD) (OPP1132237 and INV-002022 to J.P.M. and R.W.S. and OPP1115782 to A.B.W.). This work was supported by the IAVI Neutralizing Antibody Center through the CAVD grant OPP1084519 and OPP1196345/INV-008813 funded by the Bill and Melinda Gates Foundation (to M.C. and A.B.W.), the Scripps Consortium for HIV Vaccine Development (CHAVD) (AI144462 to M.C.), the European Union's Horizon 2020 research and innovation programme (681137 to R.W.S. and M.C.), the Aids Fonds (2016019 to R.W.S.), and by the Foundation Dormeur, Vaduz (to R.W.S. and M.J.v.G.). R.W.S. is a recipient of a Vici grant from the Netherlands Organisation for Scientific Research (NWO). M.J.v.G. is a recipient of an AMC Fellowship and a Mathilde Krim Fellowship from the American Foundation for AIDS Research (amfAR) (109514-61-RKVA). C.A.C. is supported by the NIH F31 Ruth L. Kirschstein Predoctoral Award AI131873 and by the Achievement Rewards for College Scientists Foundation. R.D. received a work visit grant from the Amsterdam Infection and Immunity Institute (AI&II). This is manuscript number 30004 from The Scripps Research Institute.

AUTHOR CONTRIBUTIONS

R.D., J.D.A., M.C., and R.W.S. designed the study. C.A.C. performed the cryo-EM analysis. J.D.A., G.E.S. and R.D. performed the overall and site-specific glycan analysis. W.-H.L., K.R., A.A., J.C., and S.W.d.T. performed the NS-EM analysis. Y.A., A.C., V.M.C.P., and M.P. produced, purified and quality controlled the Expi-CHO-S and HxB2 produced material. R.D. and K.S. performed ELISA experiments. R.D., P.v.d.W., and T.L.G.M.v.d.K performed the virus neutralization assays. A.Y. and P.J.K. performed the surface plasmon resonance analysis. R.D. performed the Biolayer interferometry analysis. M.J.v.G. isolated the RM20E and RM20E1 MAbs. R.D., J.D.A., K.S., P.J.K., G.O., J.P.M., A.B.W., M.C., and R.W.S. wrote the manuscript.

DECLARATION OF INTERESTS

The International AIDS Vaccine Initiative (IAVI) has previously filed a patent relating to the BG505 SOSIP.664 trimer: U.S. provisional application 61/772,739, entitled "HIV-1 Envelope Glycoprotein," with J.P.M., A.B.W., and R.W.S. among the co-inventors, but no patents have been filed on any work described here.

Received: July 22, 2020
Revised: December 10, 2020
Accepted: March 11, 2021
Published: April 6, 2021

REFERENCES

Allan, J.S., Coligan, J.E., Barin, F., McLane, M.F., Sodroski, J.G., Rosen, C.A., Haseltine, W.A., Lee, T.H., and Essex, M. (1985). Major glycoprotein antigens that induce antibodies in AIDS patients are encoded by HTLV-III. Science *228*, 1091–1094.

Bause, E. (1984). Model studies on N-glycosylation of proteins. Biochem. Soc. Trans. *12*, 514–517.

Behrens, A.-J., Vasiljevic, S., Pritchard, L.K., Harvey, D.J., Andev, R.S., Krumm, S.A., Struwe, W.B., Cupo, A., Kumar, A., Zitzmann, N., et al. (2016). Composition and Antigenic Effects of Individual Glycan Sites of a Trimeric HIV-1 Envelope Glycoprotein. Cell Rep. *14*, 2695–2706.

Blattner, C., Lee, J.H., Sliepen, K., Derking, R., Falkowska, E., de la Peña, A.T., Cupo, A., Julien, J.-P., van Gils, M., Lee, P.S., et al. (2014). Structural delineation of a quaternary, cleavage-dependent epitope at the gp41-gp120 interface on intact HIV-1 Env trimers. Immunity *40*, 669–680.

Bonomelli, C., Doores, K.J., Dunlop, D.C., Thaney, V., Dwek, R.A., Burton, D.R., Crispin, M., and Scanlan, C.N. (2011). The glycan shield of HIV is predominantly oligomannose independently of production system or viral clade. PLoS ONE *6*, e23521.

Bonsignori, M., Hwang, K.-K., Chen, X., Tsao, C.-Y., Morris, L., Gray, E., Marshall, D.J., Crump, J.A., Kapiga, S.H., Sam, N.E., et al. (2011). Analysis of a clonal lineage of HIV-1 envelope V2/V3 conformational epitope-specific broadly neutralizing antibodies and their inferred unmutated common ancestors. J. Virol. *85*, 9998–10009.

Bontjer, I., Land, A., Eggink, D., Verkade, E., Tuin, K., Baldwin, C., Pollakis, G., Paxton, W.A., Braakman, I., Berkhout, B., and Sanders, R.W. (2009). Optimization of human immunodeficiency virus type 1 envelope glycoproteins with V1/V2 deleted, using virus evolution. J. Virol. *83*, 368–383.

Brouwer, P.J.M., Antanasijevic, A., de Gast, M., Allen, J.D., Bijl, T.P.L., Yasmeen, A., Ravichandran, R., Burger, J.A., Ozorowski, G., Torres, J.L., et al. (2021). Immunofocusing and enhancing autologous Tier-2 HIV-1 neutralization by displaying Env trimers on two-component protein nanoparticles. NPJ Vaccines *6*, 24.

Calarese, D.A., Scanlan, C.N., Zwick, M.B., Deechongkit, S., Mimura, Y., Kunert, R., Zhu, P., Wormald, M.R., Stanfield, R.L., Roux, K.H., et al. (2003). Antibody domain exchange is an immunological solution to carbohydrate cluster recognition. Science *300*, 2065–2071.

Cao, L., Diedrich, J.K., Kulp, D.W., Pauthner, M., He, L., Park, S.R., Sok, D., Su, C.Y., Delahunty, C.M., Menis, S., et al. (2017). Global site-specific N-glycosylation analysis of HIV envelope glycoprotein. Nat. Commun. *8*, 14954.

Cao, L., Pauthner, M., Andrabi, R., Rantalainen, K., Berndsen, Z., Diedrich, J.K., Menis, S., Sok, D., Bastidas, R., Park, S.R., et al. (2018). Differential processing of HIV envelope glycans on the virus and soluble recombinant trimer. Nat. Commun. *9*, 3693.

Cherepanova, N., Shrimal, S., and Gilmore, R. (2016). N-linked glycosylation and homeostasis of the endoplasmic reticulum. Curr. Opin. Cell Biol. *41*, 57–65.

Cottrell, C.A., van Schooten, J., Bowman, C.A., Yuan, M., Oyen, D., Shin, M., Morpurgo, R., van der Woude, P., van Breemen, M., Torres, J.L., et al. (2020). Mapping the immunogenic landscape of near-native HIV-1 envelope trimers in non-human primates. PLoS Pathog. *16*, e1008753.

Crispin, M., Ward, A.B., and Wilson, I.A. (2018). Structure and Immune Recognition of the HIV Glycan Shield. Annu. Rev. Biophys. *47*, 499–523.

Cupo, A., Cruz Portillo, V.M., Gelfand, P., Yasmeen, A., Klasse, P.J., and Moore, J.P. (2019). Optimizing the production and affinity purification of HIV-1 envelope glycoprotein SOSIP trimers from transiently transfected CHO cells. PLoS ONE *14*, e0215106.

de Taeye, S.W., Ozorowski, G., Torrents de la Peña, A., Guttman, M., Julien, J.-P., van den Kerkhof, T.L.G.M., Burger, J.A., Pritchard, L.K., Pugach, P., Yasmeen, A., et al. (2015). Immunogenicity of Stabilized HIV-1 Envelope Trimers with Reduced Exposure of Non-neutralizing Epitopes. Cell *163*, 1702–1715.

de Taeye, S.W., Go, E.P., Sliepen, K., de la Peña, A.T., Badal, K., Medina-Ramírez, M., Lee, W.-H., Desaire, H., Wilson, I.A., Moore, J.P., et al. (2019). Stabilization of the V2 loop improves the presentation of V2 loop-associated broadly neutralizing antibody epitopes on HIV-1 envelope trimers. J. Biol. Chem. *294*, 5616–5631.

Dey, A.K., Cupo, A., Ozorowski, G., Sharma, V.K., Behrens, A.-J., Go, E.P., Ketas, T.J., Yasmeen, A., Klasse, P.J., Sayeed, E., et al. (2018). cGMP production and analysis of BG505 SOSIP.664, an extensively glycosylated, trimeric HIV-1 envelope glycoprotein vaccine candidate. Biotechnol. Bioeng. *115*, 885–899.

Doms, R.W., Lamb, R.A., Rose, J.K., and Helenius, A. (1993). Folding and assembly of viral membrane proteins. Virology *193*, 545–562.

Doores, K.J., Bonomelli, C., Harvey, D.J., Vasiljevic, S., Dwek, R.A., Burton, D.R., Crispin, M., and Scanlan, C.N. (2010). Envelope glycans of immunodeficiency virions are almost entirely oligomannose antigens. Proc. Natl. Acad. Sci. USA *107*, 13800–13805.

Duan, H., Chen, X., Boyington, J.C., Cheng, C., Zhang, Y., Jafari, A.J., Stephens, T., Tsybovsky, Y., Kalyuzhniy, O., Zhao, P., et al. (2018). Glycan Masking Focuses Immune Responses to the HIV-1 CD4-Binding Site and Enhances Elicitation of VRC01-Class Precursor Antibodies. Immunity *49*, 301–311.e5.

Dumont, J., Euwart, D., Mei, B., Estes, S., and Kshirsagar, R. (2016). Human cell lines for biopharmaceutical manufacturing: history, status, and future perspectives. Crit. Rev. Biotechnol. *36*, 1110–1122.

Earl, P.L., Moss, B., and Doms, R.W. (1991). Folding, interaction with GRP78-BiP, assembly, and transport of the human immunodeficiency virus type 1 envelope protein. J. Virol. *65*, 2047–2055.

Eggink, D., Langedijk, J.P.M., Bonvin, A.M.J.J., Deng, Y., Lu, M., Berkhout, B., and Sanders, R.W. (2009). Detailed mechanistic insights into HIV-1 sensitivity to three generations of fusion inhibitors. J. Biol. Chem. *284*, 26941–26950.

Escolano, A., Gristick, H.B., Abernathy, M.E., Merkenschlager, J., Gautam, R., Oliveira, T.Y., Pai, J., West, A.P., Jr., Barnes, C.O., Cohen, A.A., et al. (2019). Immunization expands B cells specific to HIV-1 V3 glycan in mice and macaques. Nature *570*, 468–473.

Euler, Z., VAN DEN Kerkhof, T.L., Kouyos, R.D., Tully, D.C., Allen, T.M., Trkola, A., Sanders, R.W., Schuitemaker, H., and VAN Gils, M.J. (2019). Lower Broadly Neutralizing Antibody Responses in Female Versus Male HIV-1 Infected Injecting Drug Users. Viruses *11*, 384.

Gelderblom, H.R., Hausmann, E.H., Ozel, M., Pauli, G., and Koch, M.A. (1987). Fine structure of human immunodeficiency virus (HIV) and immunolocalization of structural proteins. Virology *156*, 171–176.

Gerber, S., Lizak, C., Michaud, G., Bucher, M., Darbre, T., Aebi, M., Reymond, J.-L., and Locher, K.P. (2013). Mechanism of bacterial oligosaccharyltransferase: in vitro quantification of sequon binding and catalysis. J. Biol. Chem. *288*, 8849–8861.

Go, E.P., Herschhorn, A., Gu, C., Castillo-Menendez, L., Zhang, S., Mao, Y., Chen, H., Ding, H., Wakefield, J.K., Hua, D., et al. (2015). Comparative Analysis of the Glycosylation Profiles of Membrane-Anchored HIV-1 Envelope Glycoprotein Trimers and Soluble gp140. J. Virol. *89*, 8245–8257.

Go, E.P., Ding, H., Zhang, S., Ringe, R.P., Nicely, N., Hua, D., Steinbock, R.T., Golabek, M., Alin, J., Alam, S.M., et al. (2017). Glycosylation Benchmark Profile for HIV-1 Envelope Glycoprotein Production Based on Eleven Env Trimers. J. Virol. *91*, e02428-16.

Guttman, M., Garcia, N.K., Cupo, A., Matsui, T., Julien, J.-P., Sanders, R.W., Wilson, I.A., Moore, J.P., and Lee, K.K. (2014). CD4-induced activation in a soluble HIV-1 Env trimer. Structure *22*, 974–984.

Hu, J.K., Crampton, J.C., Cupo, A., Ketas, T., van Gils, M.J., Sliepen, K., de Taeye, S.W., Sok, D., Ozorowski, G., Deresa, I., et al. (2015). Murine Antibody Responses to Cleaved Soluble HIV-1 Envelope Trimers Are Highly Restricted in Specificity. J. Virol. *89*, 10383–10398.

Huang, J., Kang, B.H., Pancera, M., Lee, J.H., Tong, T., Feng, Y., Imamichi, H., Georgiev, I.S., Chuang, G.-Y., Druz, A., et al. (2014). Broad and potent HIV-1 neutralization by a human antibody that binds the gp41-gp120 interface. Nature *515*, 138–142.

Julien, J.-P., Lee, J.H., Cupo, A., Murin, C.D., Derking, R., Hoffenberg, S., Caulfield, M.J., King, C.R., Marozsan, A.J., Klasse, P.J., et al. (2013a). Asymmetric recognition of the HIV-1 trimer by broadly neutralizing antibody PG9. Proc. Natl. Acad. Sci. USA *110*, 4351–4356.

Julien, J.-P., Cupo, A., Sok, D., Stanfield, R.L., Lyumkis, D., Deller, M.C., Klasse, P.-J., Burton, D.R., Sanders, R.W., Moore, J.P., et al. (2013b). Crystal structure of a soluble cleaved HIV-1 envelope trimer. Science *342*, 1477–1483.

Julien, J.-P., Lee, J.H., Ozorowski, G., Hua, Y., Torrents de la Peña, A., de Taeye, S.W., Nieusma, T., Cupo, A., Yasmeen, A., Golabek, M., et al. (2015). Design and structure of two HIV-1 clade C SOSIP.664 trimers that increase the arsenal of native-like Env immunogens. Proc. Natl. Acad. Sci. USA *112*, 11947–11952.

Kasturi, L., Eshleman, J.R., Wunner, W.H., and Shakin-Eshleman, S.H. (1995). The hydroxy amino acid in an Asn-X-Ser/Thr sequon can influence N-linked core glycosylation efficiency and the level of expression of a cell surface glycoprotein. J. Biol. Chem. *270*, 14756–14761.

Klasse, P.J., LaBranche, C.C., Ketas, T.J., Ozorowski, G., Cupo, A., Pugach, P., Ringe, R.P., Golabek, M., van Gils, M.J., Guttman, M., et al. (2016). Sequential and Simultaneous Immunization of Rabbits with HIV-1 Envelope Glycoprotein SOSIP.664 Trimers from Clades A, B and C. PLoS Pathog. *12*, e1005864.

Klasse, P.J., Ketas, T.J., Cottrell, C.A., Ozorowski, G., Debnath, G., Camara, D., Francomano, E., Pugach, P., Ringe, R.P., LaBranche, C.C., et al. (2018). Epitopes for neutralizing antibodies induced by HIV-1 envelope glycoprotein BG505 SOSIP trimers in rabbits and macaques. PLoS Pathog. *14*, e1006913.

Klasse, P.J., Ozorowski, G., Sanders, R.W., and Moore, J.P. (2020). Env Exceptionalism: Why Are HIV-1 Env Glycoproteins Atypical Immunogens? Cell Host Microbe *27*, 507–518.

Klein, F., Gaebler, C., Mouquet, H., Sather, D.N., Lehmann, C., Scheid, J.F., Kraft, Z., Liu, Y., Pietzsch, J., Hurley, A., et al. (2012). Broad neutralization by a combination of antibodies recognizing the CD4 binding site and a new conformational epitope on the HIV-1 envelope protein. J. Exp. Med. *209*, 1469–1479.

Kumar, R., Ozorowski, G., Kumar, V., Holden, L.G., Shrivastava, T., Patil, S., Deshpande, S., Ward, A.B., and Bhattacharya, J. (2017). Characterization of a stable HIV-1 B/C recombinant, soluble, and trimeric envelope glycoprotein (Env) highly resistant to CD4-induced conformational changes. J. Biol. Chem. *292*, 15849–15858.

Land, A., and Braakman, I. (2001). Folding of the human immunodeficiency virus type 1 envelope glycoprotein in the endoplasmic reticulum. Biochimie *83*, 783–790.

Lee, J.H., Andrabi, R., Su, C.-Y., Yasmeen, A., Julien, J.-P., Kong, L., Wu, N.C., McBride, R., Sok, D., Pauthner, M., et al. (2017). A Broadly Neutralizing Antibody Targets the Dynamic HIV Envelope Trimer Apex via a Long, Rigidified, and Anionic β-Hairpin Structure. Immunity *46*, 690–702.

Leonard, C.K., Spellman, M.W., Riddle, L., Harris, R.J., Thomas, J.N., and Gregory, T.J. (1990). Assignment of intrachain disulfide bonds and characterization of potential glycosylation sites of the type 1 recombinant human immunodeficiency virus envelope glycoprotein (gp120) expressed in Chinese hamster ovary cells. J. Biol. Chem. *265*, 10373–10382.

Li, Y., Luo, L., Rasool, N., and Kang, C.Y. (1993). Glycosylation is necessary for the correct folding of human immunodeficiency virus gp120 in CD4 binding. J. Virol. *67*, 584–588.

Lyumkis, D., Julien, J.-P., de Val, N., Cupo, A., Potter, C.S., Klasse, P.-J., Burton, D.R., Sanders, R.W., Moore, J.P., Carragher, B., et al. (2013). Cryo-EM structure of a fully glycosylated soluble cleaved HIV-1 envelope trimer. Science *342*, 1484–1490.

McCoy, L.E., van Gils, M.J., Ozorowski, G., Messmer, T., Briney, B., Voss, J.E., Kulp, D.W., Macauley, M.S., Sok, D., Pauthner, M., et al. (2016). Holes in the Glycan Shield of the Native HIV Envelope Are a Target of Trimer-Elicited Neutralizing Antibodies. Cell Rep. *16*, 2327–2338.

Mellquist, J.L., Kasturi, L., Spitalnik, S.L., and Shakin-Eshleman, S.H. (1998). The amino acid following an asn-X-Ser/Thr sequon is an important determinant of N-linked core glycosylation efficiency. Biochemistry *37*, 6833–6837.

Nogal, B., Bianchi, M., Cottrell, C.A., Kirchdoerfer, R.N., Sewall, L.M., Turner, H.L., Zhao, F., Sok, D., Burton, D.R., Hangartner, L., and Ward, A.B. (2020). Mapping Polyclonal Antibody Responses in Non-human Primates Vaccinated with HIV Env Trimer Subunit Vaccines. Cell Rep. *30*, 3755–3765.e7.

Peden, K., Emerman, M., and Montagnier, L. (1991). Changes in growth properties on passage in tissue culture of viruses derived from infectious molecular clones of HIV-1LAI, HIV-1MAL, and HIV-1ELI. Virology *185*, 661–672.

Pritchard, L.K., Spencer, D.I.R., Royle, L., Bonomelli, C., Seabright, G.E., Behrens, A.-J., Kulp, D.W., Menis, S., Krumm, S.A., Dunlop, D.C., et al. (2015a). Glycan clustering stabilizes the mannose patch of HIV-1 and preserves vulnerability to broadly neutralizing antibodies. Nat. Commun. *6*, 7479.

Pritchard, L.K., Vasiljevic, S., Ozorowski, G., Seabright, G.E., Cupo, A., Ringe, R., Kim, H.J., Sanders, R.W., Doores, K.J., Burton, D.R., et al. (2015b). Structural Constraints Determine the Glycosylation of HIV-1 Envelope Trimers. Cell Rep. *11*, 1604–1613.

Pritchard, L.K., Harvey, D.J., Bonomelli, C., Crispin, M., and Doores, K.J. (2015c). Cell- and Protein-Directed Glycosylation of Native Cleaved HIV-1 Envelope. J. Virol. *89*, 8932–8944.

Pugach, P., Ozorowski, G., Cupo, A., Ringe, R., Yasmeen, A., de Val, N., Derking, R., Kim, H.J., Korzun, J., Golabek, M., et al. (2015). A native-like SOSIP.664 trimer based on an HIV-1 subtype B env gene. J. Virol. *89*, 3380–3395.

Punjani, A., Rubinstein, J.L., Fleet, D.J., and Brubaker, M.A. (2017). cryoSPARC: algorithms for rapid unsupervised cryo-EM structure determination. Nat. Methods *14*, 290–296.

Ringe, R.P., Pugach, P., Cottrell, C.A., LaBranche, C.C., Seabright, G.E., Ketas, T.J., Ozorowski, G., Kumar, S., Schorcht, A., van Gils, M.J., et al. (2019). Closing and Opening Holes in the Glycan Shield of HIV-1 Envelope Glycoprotein SOSIP Trimers Can Redirect the Neutralizing Antibody Response to the Newly Unmasked Epitopes. J. Virol. *93*, e01656-18.

Ruiz-Canada, C., Kelleher, D.J., and Gilmore, R. (2009). Cotranslational and posttranslational N-glycosylation of polypeptides by distinct mammalian OST isoforms. Cell *136*, 272–283.

Sanders, R.W., Vesanen, M., Schuelke, N., Master, A., Schiffner, L., Kalyanaraman, R., Paluch, M., Berkhout, B., Maddon, P.J., Olson, W.C., et al. (2002). Stabilization of the soluble, cleaved, trimeric form of the envelope glycoprotein complex of human immunodeficiency virus type 1. J. Virol. *76*, 8875–8889.

Sanders, R.W., Derking, R., Cupo, A., Julien, J.-P., Yasmeen, A., de Val, N., Kim, H.J., Blattner, C., de la Peña, A.T., Korzun, J., et al. (2013). A next-generation cleaved, soluble HIV-1 Env trimer, BG505 SOSIP.664 gp140, expresses multiple epitopes for broadly neutralizing but not non-neutralizing antibodies. PLoS Pathog. *9*, e1003618.

Sanders, R.W., van Gils, M.J., Derking, R., Sok, D., Ketas, T.J., Burger, J.A., Ozorowski, G., Cupo, A., Simonich, C., Goo, L., et al. (2015). HIV-1 VACCINES. HIV-1 neutralizing antibodies induced by native-like envelope trimers. Science *349*, aac4223.

Scheid, J.F., Mouquet, H., Ueberheide, B., Diskin, R., Klein, F., Oliveira, T.Y.K., Pietzsch, J., Fenyo, D., Abadir, A., Velinzon, K., et al. (2011). Sequence and structural convergence of broad and potent HIV antibodies that mimic CD4 binding. Science *333*, 1633–1637.

Schorcht, A., van den Kerkhof, T.L.G.M., Cottrell, C.A., Allen, J.D., Torres, J.L., Behrens, A.-J., Schermer, E.E., Burger, J.A., de Taeye, S.W., Torrents de la Peña, A., et al. (2020). Neutralizing antibody responses induced by HIV-1 envelope glycoprotein SOSIP trimers derived from elite neutralizers. J. Virol. *94*, e01214-20.

Schülke, N., Vesanen, M.S., Sanders, R.W., Zhu, P., Lu, M., Anselma, D.J., Villa, A.R., Parren, P.W.H.I., Binley, J.M., Roux, K.H., et al. (2002). Oligomeric and conformational properties of a proteolytically mature, disulfide-stabilized human immunodeficiency virus type 1 gp140 envelope glycoprotein. J. Virol. *76*, 7760–7776.

Seabright, G.E., Doores, K.J., Burton, D.R., and Crispin, M. (2019). Protein and Glycan Mimicry in HIV Vaccine Design. J. Mol. Biol. *431*, 2223–2247.

Sellhorn, G., Caldwell, Z., Mineart, C., and Stamatatos, L. (2009). Improving the expression of recombinant soluble HIV Envelope glycoproteins using pseudo-stable transient transfection. Vaccine *28*, 430–436.

Shrimal, S., and Gilmore, R. (2013). Glycosylation of closely spaced acceptor sites in human glycoproteins. J. Cell Sci. *126*, 5513–5523.

Sliepen, K., Han, B.W., Bontjer, I., Mooij, P., Garces, F., Behrens, A.-J., Rantalainen, K., Kumar, S., Sarkar, A., Brouwer, P.J.M., et al. (2019). Structure and immunogenicity of a stabilized HIV-1 envelope trimer based on a group-M consensus sequence. Nat. Commun. *10*, 2355.

Snapp, E.L., McCaul, N., Quandte, M., Cabartova, Z., Bontjer, I., Källgren, C., Nilsson, I., Land, A., von Heijne, G., Sanders, R.W., and Braakman, I. (2017). Structure and topology around the cleavage site regulate post-translational cleavage of the HIV-1 gp160 signal peptide. eLife *6*, e26067.

Sok, D., van Gils, M.J., Pauthner, M., Julien, J.-P., Saye-Francisco, K.L., Hsueh, J., Briney, B., Lee, J.H., Le, K.M., Lee, P.S., et al. (2014). Recombinant

HIV envelope trimer selects for quaternary-dependent antibodies targeting the trimer apex. Proc. Natl. Acad. Sci. USA *111*, 17624–17629.

Steichen, J.M., Kulp, D.W., Tokatlian, T., Escolano, A., Dosenovic, P., Stanfield, R.L., McCoy, L.E., Ozorowski, G., Hu, X., Kalyuzhniy, O., et al. (2016). HIV Vaccine Design to Target Germline Precursors of Glycan-Dependent Broadly Neutralizing Antibodies. Immunity *45*, 483–496.

Struwe, W.B., Chertova, E., Allen, J.D., Seabright, G.E., Watanabe, Y., Harvey, D.J., Medina-Ramirez, M., Roser, J.D., Smith, R., Westcott, D., et al. (2018). Site-Specific Glycosylation of Virion-Derived HIV-1 Env Is Mimicked by a Soluble Trimeric Immunogen. Cell Rep. *24*, 1958–1966.e5.

Torrents de la Peña, A., Julien, J.-P., de Taeye, S.W., Garces, F., Guttman, M., Ozorowski, G., Pritchard, L.K., Behrens, A.-J., Go, E.P., Burger, J.A., et al. (2017). Improving the Immunogenicity of Native-like HIV-1 Envelope Trimers by Hyperstabilization. Cell Rep. *20*, 1805–1817.

Torrents de la Peña, A., de Taeye, S.W., Sliepen, K., LaBranche, C.C., Burger, J.A., Schermer, E.E., Montefiori, D.C., Moore, J.P., Klasse, P.J., and Sanders, R.W. (2018). Immunogenicity in Rabbits of HIV-1 SOSIP Trimers from Clades A, B, and C, Given Individually, Sequentially, or in Combination. J. Virol. *92*, e01957-17.

Torrents de la Peña, A., Rantalainen, K., Cottrell, C.A., Allen, J.D., van Gils, M.J., Torres, J.L., Crispin, M., Sanders, R.W., and Ward, A.B. (2019). Similarities and differences between native HIV-1 envelope glycoprotein trimers and stabilized soluble trimer mimetics. PLoS Pathog. *15*, e1007920.

van Gils, M.J., van den Kerkhof, T.L.G.M., Ozorowski, G., Cottrell, C.A., Sok, D., Pauthner, M., Pallesen, J., de Val, N., Yasmeen, A., de Taeye, S.W., et al. (2016). An HIV-1 antibody from an elite neutralizer implicates the fusion peptide as a site of vulnerability. Nat. Microbiol. *2*, 16199.

Wagh, K., Kreider, E.F., Li, Y., Barbian, H.J., Learn, G.H., Giorgi, E., Hraber, P.T., Decker, T.G., Smith, A.G., Gondim, M.V., et al. (2018). Completeness of HIV-1 Envelope Glycan Shield at Transmission Determines Neutralization Breadth. Cell Rep. *25*, 893–908.e7.

Walker, L.M., Huber, M., Doores, K.J., Falkowska, E., Pejchal, R., Julien, J.-P., Wang, S.-K., Ramos, A., Chan-Hui, P.-Y., Moyle, M., et al.; Protocol G Principal Investigators (2011). Broad neutralization coverage of HIV by multiple highly potent antibodies. Nature *477*, 466–470.

Wei, X., Decker, J.M., Wang, S., Hui, H., Kappes, J.C., Wu, X., Salazar-Gonzalez, J.F., Salazar, M.G., Kilby, J.M., Saag, M.S., et al. (2003). Antibody neutralization and escape by HIV-1. Nature *422*, 307–312.

Wu, X., Parast, A.B., Richardson, B.A., Nduati, R., John-Stewart, G., Mbori-Ngacha, D., Rainwater, S.M.J., and Overbaugh, J. (2006). Neutralization escape variants of human immunodeficiency virus type 1 are transmitted from mother to infant. J. Virol. *80*, 835–844.

Wu, X., Yang, Z.-Y., Li, Y., Hogerkorp, C.-M., Schief, W.R., Seaman, M.S., Zhou, T., Schmidt, S.D., Wu, L., Xu, L., et al. (2010). Rational design of envelope identifies broadly neutralizing human monoclonal antibodies to HIV-1. Science *329*, 856–861.

Yasmeen, A., Ringe, R., Derking, R., Cupo, A., Julien, J.-P., Burton, D.R., Ward, A.B., Wilson, I.A., Sanders, R.W., Moore, J.P., and Klasse, P.J. (2014). Differential binding of neutralizing and non-neutralizing antibodies to native-like soluble HIV-1 Env trimers, uncleaved Env proteins, and monomeric subunits. Retrovirology *11*, 41.

Zhou, T., Doria-Rose, N.A., Cheng, C., Stewart-Jones, G.B.E., Chuang, G.-Y., Chambers, M., Druz, A., Geng, H., McKee, K., Kwon, Y.D., et al. (2017). Quantification of the Impact of the HIV-1-Glycan Shield on Antibody Elicitation. Cell Rep. *19*, 719–732.

STAR★METHODS

KEY RESOURCE TABLE

REAGENT or RESOURCE	SOURCE	IDENTIFIER
Antibodies		
VRC01	This paper	RRID: AB_2491019
3BNC60	This paper	RRID: AB_2491038
PG9	This paper	RRID: AB_2491030
PG16	This paper	RRID: AB_2491031
PGT145	This paper	RRID: AB_2491054
PGDM1400	This paper	N/A
CH01	This paper	RRID: AB_2491055
PGT121	This paper	RRID: AB_2491041
PGT122	This paper	RRID: AB_2491042
PGT123	This paper	RRID: AB_2491043
PGT125	This paper	RRID: AB_2491044
PGT126	This paper	RRID: AB_2491045
PGT128	This paper	RRID: AB_2491047
PGT130	This paper	RRID: AB_2491048
2G12	This paper	RRID: AB_2819235
PGT135	This paper	RRID: AB_2491050
PGT151	This paper	N/A
35O22	This paper	N/A
3BC315	This paper	N/A
HIV-Ig	This paper	N/A
Goat Anti-Human IgG (HRP)	SeraCare	Cat# 5220-0277
CA13 (ARP3119)	EU Programme EVA Centralized Facility for AIDS Reagents, NIBSC	N/A
Anti-HIV-1 gp120	Aalto Bio Reagents	Cat# D7324
Cell lines		
HEK293F cells	Thermo Fisher Scientific	Cat# R79007
HEK293T cells	American Type Culture Collection	Cat.# 11268
ExpiCHO-S cells	Thermo Fisher Scientific	Cat# A29127
Chemicals, peptides, and recombinant proteins		
FectoCHO CD Expression Media	VWR	Cat# 76234-742
FectoPRO DNA transfection kit	VWR	Cat# 10118-842
FreeStyle 293F media	Thermo Fisher Scientific	Cat# 12338026
Opti-MEM Reduced Serum Medium	Thermo Fisher Scientific	Cat# 31985070
PEI MAX transfection reagent	Polysciences	Cat# 24765-1
Lipofectamine 2000	Life Technologies	Cat# 11668-019
Dulbecco's Modified Eagles Medium	Life Technologies	Cat# 41966052
Acetonitrile, 80%, 20% Water with 0.1% Formic Acid, Optima LC/MS	Fisher Scientific	Cat# 15431423
Water with 0.1% Formic Acid (v/v), Optima LC/MS Grade	Fisher Scientific	Cat# LS118-212
Acetonitrile	Fisher Scientific	Cat# 10489553
Trifluoroacetic acid	Fisher Scientific	Cat# 10155347
Procainamide hydrochloride	Abcam	Cat# ab120955

(*Continued on next page*)

Continued

REAGENT or RESOURCE	SOURCE	IDENTIFIER
H_2O^{18}	Sigma-Aldrich	Cat# 329878
Dithiothreitol	Sigma-Aldrich	Cat# 43819
Iodacetamide	Sigma-Aldrich	Cat# I1149
Ammonium formate buffer	Waters	Cat# 186007081
Sodium cyanoborohydride	Sigma-Aldrich	Cat# 156159
DMSO	Sigma-Aldrich	Cat# D2438
Acetic acid	Fisher Scientific	Cat# 10384970
Peptide-N-glycosidase F	New England Biolabs	Cat# P0705S
Endoglycosidase H	New England Biolabs	Cat# P0702S
Mass spectrometry grade trypsin	Promega	Cat# V5280
Sequencing grade chymotrypsin	Promega	Cat# V1061
HBS-EP buffer	Cytiva	Cat# BR100188
Sensor Chips, CM5	Cytiva	Cat# 29149604
Aqueous buffer, 10mM Glycine-HCl pH2.0	Cytiva	Cat# BR100355
Streptavidin (SA) biosensors	Fortébio	Cat#18-5019
DpnI	New England Biolabs	Cat# R0176S
Penicillin	Sigma-Aldrich	P3032-10MU
Streptomycin	VWR	382-EU-100G
GIBCO DPBS	Life Technologies	Cat# 12559069
Glycyl Glycine 99+%	Fisher Scientific	Cat# 10540771
$MgSO_4$	VWR	Cat# 10034-99-8
TitriPlex III (EDTA)	VWR	Cat# 1.08418.1000
Triton X-100	Fisher Scientific	Cat# BP151500
Tris	Sigma-Aldrich	Cat# 10708976001
HCl	Biosolve	Cat# 084105
Glycine	VWR	Cat# 4500345965
Magnesium Chloride ($MgCl_2$)	VWR	Cat# 4500348228
Sodium Bicarbonate ($NaHCO_3$)	Life Technologies	Cat# 25080094
Sodium Chloride (NaCl)	Sigma-Aldrich	Cat# S7653-1KG
Sodium Acetate (NaAc)	VWR	Cat# 1.06268.1000
Citric Acid Monohydrate	Brunschwig	Cat# 36665.22
3,3′,5,5′-Tetramethylbenzidine (TMB)	Sigma-Aldrich	Cat# T-2885
H_2O_2	Brunschwig	Cat# CP26.1
Sulfuric Acid 95-97%	VWR	Cat# 1.00731.1010
Sodium Dodecyl Sulfate	Sigma	Cat# L5750-1kg
Glycerol	Thermo Fisher Scientific	Cat# 15514-011
Methanol	Biosolve	Cat# 13680502
Nonfat Dried Milk Powder	VWR	Cat# A0830.0500
Tween-20	Antibody chain (santacruz)	Cat# SC-29113
Precision Plus Protein Standard Dual Color	Bio-Rad	Cat# 161-0374
Western Lightning Plus-ECL	PerkinElmer	Cat# NEL103001EA
NativePAGE Running Buffer	Thermo Fisher Scientific	Cat# BN2001
NativePAGE Cathode Buffer Additive	Thermo Fisher Scientific	Cat# BN2002
MOPS	Sigma	Cat# M1254-250G
Coomassie Brilliant Blue G-250	Fluka	Cat# 27815
HMW-Native Protein Mixture	Cytiva	Cat# 17-0445-01
Bovine Serum Albumine	Sigma	Cat# A7637-10 g

(*Continued on next page*)

CellPress
OPEN ACCESS

Continued

REAGENT or RESOURCE	SOURCE	IDENTIFIER
Critical commercial assays		
In-Fusion HD cloning plus	Takara	Cat# 638910
QuickChange site-directed mutagenesis kit	Agilent	Cat# 200518
NucleoSpin PCR clean-up kit	Macherey-Nagel	Cat# 740609.50
Bright-Glo Luciferase Assay system	Promega	Cat# E2650
Deposited data		
BG505 SOSIPv5.2 in complex with PGT122 and two RM20E1 Fabs	This Study	EMD-22170
BG505 SOSIPv5.2 in complex with PGT122 and three RM20E1 Fabs	This Study	EMD-22171
C3 symmetric BG505 SOSIPv5.2 in complex with PGT122 and three RM20E1 Fabs	This Study	EMD-22172
BG505 SOSIPv5.2 in complex with PGT122 and one RM20E1 Fab	This Study	EMD-22173
BG505 SOSIPv5.2 in complex with PGT122 and no RM20E1 Fabs	This Study	EMD-22178
C3 symmetric BG505 SOSIPv5.2 in complex with PGT122 and no RM20E1 Fabs	This Study	EMD-22179
Recombinant DNA		
BG505 SOSIP.v4.1	de Taeye et al., 2015	N/A
BG505 SOSIP.v5	Torrents de la Peña et al., 2017	N/A
VRC01 light and heavy chains	Wu et al., 2010	N/A
3BNC60 light and heavy chains	Scheid et al., 2011	N/A
PG9 light and heavy chains	Walker et al., 2011	N/A
PG16 light and heavy chains	Walker et al., 2011	N/A
PGT145 light and heavy chains	Walker et al., 2011	N/A
PGDM1400 light and heavy chains	Sok et al., 2014	N/A
CH01 light and heavy chains	Bonsignori et al., 2011	N/A
PGT121 light and heavy chains	Walker et al., 2011	N/A
PGT122 light and heavy chains	Walker et al., 2011	N/A
PGT123 light and heavy chains	Walker et al., 2011	N/A
PGT125 light and heavy chains	Walker et al., 2011	N/A
PGT126 light and heavy chains	Walker et al., 2011	N/A
PGT128 light and heavy chains	Walker et al., 2011	N/A
PGT130 light and heavy chains	Walker et al., 2011	N/A
2G12 light and heavy chains	Calarese et al., 2003	N/A
PGT135 light and heavy chains	Walker et al., 2011	N/A
PGT151 light and heavy chains	Blattner et al., 2014	N/A
35O22 light and heavy chains	Huang et al., 2014	N/A
3BC315 light and heavy chains	Klein et al., 2012	N/A
Polyclonal HIV-Ig	NIH AIDS Reagent Program	N/A
Software and algorithms		
Empower 3.0	Waters	N/A
Masslynx v4.1	Waters	N/A
Driftscope version 2.8	Waters	N/A
Byos^{TM} (Version 3.9)	Protein Metrics Inc.	N/A
GraphPad Prism v8	GraphPad	N/A
XCalibur Version v4.2	Thermo Fisher	N/A
Orbitrap Fusion Tune application v3.1	Thermo Fisher	N/A

(*Continued on next page*)

Continued		
REAGENT or RESOURCE	SOURCE	IDENTIFIER
Other		
Superdex 200 Increase 10/300 SEC column	Cytiva	Cat# 28-9909-44
SDS-PAGE 8% Tris-Glycine gel	Invitrogen	Cat# XP00085BOX
SDS-PAGE 4-20% Tris-glycine gel	Invitrogen	Cat# XP04205BOX
NuPAGE 4-12% Bis-Tris gel	Invitrogen	Cat# NP0321BOX
Econo-Column® Chromatography Columns	Bio-Rad	Cat# 7371512
Glycan BEH Amide column (2.1 mm x 100 mm, 1.7 μM)	Waters	Cat# 186004741
EasySpray PepMap RSLC C18 column (75 μm x 75 cm)	Thermo Fisher Scientific	Cat# ES805
PVDF protein-binding membrane	Millipore	Cat# MAIPS4510
C18 ZipTip	Merck Milipore	Cat# ZTC18S008
Spe-ed Amide 2 cartridges	Applied Separations	Cat# 4821
Vivaspin 500, 3 kDa MWCO, Polyethersulfone	Sigma-Aldrich	Cat# GE28-9322-18
Vivaspin 20, 100.000 MWCO PES	Sartorius	Cat# VS2042
PepMap100 C18 3UM 75UMx2CM Nanoviper	Thermo Scientific	164946
Steritop-GP Filter Unit 0.22μm	Millipore	Cat# SCGPT05RE
CNBr-activated Sepharose4B	Cytiva	Cat# 17-0430-01
ELISA-plate, half-area, 96W	Greiner Bio One	Cat# 675061
Hissorb 96-well ELISA plates	QIAGEN	Cat# 35061

RESOURCE AVAILABILITY

Lead contact

Further information and requests for resources and reagents should be directed to and will be fulfilled by the Lead Contact; Rogier W. Sanders (r.w.sanders@amsterdamumc.nl).

Materials availability

This study did not generate new unique reagents. All expression plasmids and lentiviral vectors generated in this study will be made available on request.

Data and code availability

The Cryo-EM 3D reconstructions have been deposited in the Electron Microscopy Data Bank (EMDB) with accession numbers: EMD-22170, EMD-22171, EMD-22172, EMD-22173, EMD-22178, EMD-22179.

EXPERIMENTAL MODEL AND SUBJECT DETAILS

HEK293T cell culture

WT and NxT proteins, and the infectious virus stocks were prepared by transfecting 293T cells (American Type Culture Collection Cat. #11268) using the PEI MAX transfection reagent or Lipofectamine transfection reagent.

HEK293F cell culture and transfection

HEK293F cells were maintained at a density of 1-3x10^6 cells per mL at 37 degrees with 8% CO_2 and 125rpm shaking. Plasmids encoding the WT, NxT, NxT T158S and NxT T135S T158S proteins containing a C-terminal D7324-tag were transiently co-transfected with a Furin-encoding plasmid (4:1) in HEK293F cells using PEI MAX transfection reagent. The cells were transfected at a density of 1x10^6 cells per ml and incubated for 6 days at 37 degrees with 8% CO2 and 125rpm shaking.

ExpiCHO-S cell culture and transfection

ExpiCHO-S cells were maintained at a density of 1-2x10^6 cells per mL at 37 degrees with 8% CO_2 and 135rpm shaking. The WT and NxT T35S T158S encoding plasmids containing a C-terminal D7324-tag were transiently co-transfected with a Furin-encoding plasmid (4:1) using FectoPRO transfection reagent as described by the manufacturer (VWR).

METHOD DETAIL

BG505 infectious molecular clones

The infectious molecular clone of LAI was used as the backbone for creating an infectious molecular clone containing BG505.T332N gp160 (Peden et al., 1991; Wu et al., 2006). This clone contains a unique Sal1 restriction site 434 nucleotides upstream of the *env* start codon and a unique BamH1 site at the codons specifying amino acids G751 and S752 in LAI gp160 (HXB2 numbering). A DNA fragment containing the LAI sequences between the Sal1 site and the *env* start codon, followed by the BG505.T332N *env* sequences up to the BamH1 site, was synthesized (Genscript, Piscataway, NJ) and cloned into the LAI molecular clone backbone using Sal1 and BamH1. The resulting molecular clone encodes the complete BG505.T332N gp160 sequence, except for the C-terminal 106 amino acids of the cytoplasmic tail, which are derived from LAI gp160. The sequence was verified before the clone was used in virus infectivity and neutralization assays. The resulting virus was able to infect TZM-bl cells and replicate in PBMCs.

The BG505.T332N sequence was modified by mutating all PNGS to either NxT (NxT virus) or NxS (NxS virus). As described above, the resulting *env* sequences, between the Sal1 and BamH1 sites, where obtained from Genscript in a pUC57 cloning vector. The Sal1 and BamH1 BG505-NxT and BG505-NxS sequences were amplified by PCR, using In-Fusion cloning (Clontech), as described by the manufacturer. After the PCR, 0.5 μl DpnI (NEB) was added to each reaction and incubated for 1 h at 37°C. Next, the PCR products were purified using a PCR clean-up kit (Macherey-Nagel) and the DNA concentrations were measured. The LAI *env* sequences between the Sal1 and BamH1 sites in the pLAI expression plasmid (Peden et al., 1991), were replaced by the BG505-NxT or BG505-NxS PCR fragments using the In-Fusion enzyme (Clontech) as described by the manufacturer. Plasmid DNA (5 μg) of the molecular clones was transfected into HEK293T cells as described previously to generate infectious virus stocks (Sanders et al., 2013).

Env SOSIP trimers

The design of BG505 SOSIP.664 and BG505 SOSIP.v4.1 trimers, including the D7324-epitope tagged versions, has been described extensively elsewhere, as have the methods to produce and purify them (Julien et al., 2013a, 2013b; Lyumkis et al., 2013; Sanders et al., 2013; de Taeye et al., 2015). The design of AMC009 and AMC011 SOSIP.v5.2 trimers has been described elsewhere (Schorcht et al., 2020). The HxB2 reference sequence has been used for the design of the HxB2 SOSIP trimer. The SOSIP trimer contains the v5 mutations and the 519S, 568D, 570H and 585H stabilizing mutations in gp41 (Steichen et al., 2016; Torrents de la Peña et al., 2017). The HxB2 NxT protein also has an PNGS added at position N411 to close a glycan hole. Env SOSIP trimer expression constructs in which all PNGS were mutated to either NxT (NxT protein) or NxS (NxS protein) were obtained from Genscript and cloned in the pPPI4 expression vector. The Env trimers were purified via PGT145-affinity chromatography and 2G12-affinity chromatography followed by SEC (Pugach et al., 2015; Sanders et al., 2013; de Taeye et al., 2015).

Antibodies

MAbs were obtained as gifts, or purchased, or expressed from plasmids, from the following sources directly or through the AIDS Reagents Reference Program: John Mascola and Peter Kwong (VRC01); Michel Nussenzweig (3BNC60, 3BC315); Dennis Burton (PG9, PG16, PGT121-123, PGT125-128, PGT130, PGT135, PGT145, PGDM1400 and PGT151); Barton Haynes (CH01); Polymun Scientific (2G12); Mark Connors (35O22); Ms C. Arnold (CA13 (ARP3119)), EU Programme EVA Centralized Facility for AIDS Reagents, NIBSC, UK (AVIP Contract Number LSHP-CT-2004-503487).

Neutralization assays

One day prior to infection, 1.7 x 10^4 TZM-bl cells per well were seeded on a 96-well plate in Dulbecco's Modified Eagles Medium (DMEM) containing 10% FCS, penicillin and streptomycin (both at 100 U/ml), and incubated at 37°C in an atmosphere containing 5% CO_2. A fixed amount of virus (2.5 ng/ml of p24-antigen equivalent) was incubated for 30 min at room temperature with serial 3-fold dilutions of each test mAb (Bontjer et al., 2009; Eggink et al., 2009; Sanders et al., 2013). This mixture was added to the cells and 40 μg/ml DEAE, in a total volume of 200 μl. Three days later, the medium was removed. The cells were washed once with PBS (150 mM NaCl, 50 mM sodium phosphate, pH 7.0) and lysed in Lysis Buffer, pH 7.8 (25 mM Glycylglycine (Gly-Gly), 15 mM $MgSO_4$, 4 mM EGTA tetrasodium, 10% Triton-X). Luciferase activity was measured using a Bright-Glo kit (Promega, Madison, WI) and a Glomax Luminometer according to the manufacturer's instructions (Turner BioSystems, Sunnyvale, CA). All infection measurements were performed in quadruple. Uninfected cells were used to correct for background luciferase activity. The infectivity of each mutant without inhibitor was set at 100%. Nonlinear regression curves were determined and 50% inhibitory concentrations (IC_{50}) were calculated using a sigmoid function in Prism software version 8.

SDS-PAGE and Blue Native-PAGE

Env proteins were analyzed using SDS-PAGE and BN-PAGE, blotted and detected by using the CA13 (ARP3119) and 2G12 MAbs. In some cases the gels were stained using Coomassie blue (Sanders et al., 2002; Schülke et al., 2002).

Negative stain electron microscopy

The WT protein and NxT PNGS mutants were analyzed by negative stain EM as previously described (Cottrell et al., 2020). For the RM20E1 complexes, SOSIP trimers were incubated with 6-fold molar excess per protomer Fab at RT overnight. The complexes were

purified using SEC on a Superose^{TM} 6 Increase 10/300 GL (GE Healthcare) column. Samples were imaged and analyzed as previously described (Cottrell et al., 2020).

Cryo electron microscopy

The cryoEM dataset that led to EMDB-21232 was reprocessed with cryoSPARCv.1 to sort particles into classes based on RM20E1 Fab occupancy (Cottrell et al., 2020; Punjani et al., 2017). Final refinements were performed using cryoSPARCv.1 (Punjani et al., 2017).

Ni-NTA-capture ELISA

The Ni-NTA-capture ELISA has been described in detail elsewhere (Sanders et al., 2013). Purified His-tagged proteins were captured on Hissorb 96-well plates (QIAGEN, Venlo, the Netherlands) and tested for Ab binding. Abs were detected with the goat-anti-human HRP-labeled Ab (SeraCare).

D7324-capture ELISA

The D7324-capture ELISA has been described in detail elsewhere (Sanders et al., 2013). Microlon 96-well half-area plates (Greiner Bio-One, Alphen aan den Rijn, the Netherlands) were coated with D7324 antibody (10 μg/ml; Aalto Bioreagents, Dublin, Ireland). Purified proteins (2.5 μg/ml) were subsequently captured on the ELISA plate wells and tested for Ab binding. Abs were detected with the goat-anti-human HRP-labeled Ab (SeraCare).

Biolayer interferometry (BLI)

Antibody binding to the PGT145-purified WT, NxT and NxT T135S T158S PNGS mutants was studied using a ForteBio Octet K2 instrument (de Taeye et al., 2019). All assays were performed at 30°C with the agitation setting at 1000 rpm. Purified proteins and antibodies were diluted in running buffer (PBS, 0.1% BSA, 0.02% Tween 20) and analyzed in a final volume of 250 μl/well. Antibody were loaded onto protein A sensors (ForteBio) at 2.0 μg/ml in running buffer until a binding threshold of 0.5 nM was reached. Trimer proteins were diluted in running buffer at 40 nM or 600 nM for CH01, and association and dissociation were measured for 300 s. Trimer binding to a protein A sensor with no loaded antibody was measured to derive background values.

Surface Plasmon Resonance (SPR)

NAb binding to the WT and NxT mutants was analyzed by surface plasmon resonance (SPR). All experiments were performed at 25°C on a Biacore 3000 instrument (Cytiva, formerly GE Healthcare). Standard HBS-EP (0.01 M HEPES, 0.15 M NaCl, 3 mM EDTA, 0.005% v/v Surfactant P20, pH 7.4) was used as running buffer throughout the analysis.

Anti-histidine antibody coupled to CM5 (a-His-CM5) sensor-chip surfaces was used for capturing His-tagged the WT and NxT proteins. The anti-histidine antibody was covalently conjugated to CM5 sensor chips by amine coupling in accordance with the manufacturers' instructions, generating immobilization levels of $\sim 10^4$ RU (response units). The purified proteins were captured on the anti-His-CM5 sensor chips to a mean density of 530 RU (SD = 9.3 RU). Fabs of PGT145 and PGT130 were titrated against His-tagged WT and NxT proteins, downward from a concentration of 1 μM, by two-fold dilution steps in running buffer, until no detectable signal was obtained. In each kinetic cycle, Fab binding was monitored for 300 s of association, followed by 600 s of dissociation. Maximum flow rate (50 μl/min) was used to minimize mass-transfer limitation, which was absent according to k_t analyses. At the end of each cycle, the sensor surface was regenerated by a single injection of glycine (10 mM; pH 2.0) for 60 s at a flow rate of 30 μl/min. Titration data were analyzed by fitting the models of the BIA-Evaluation software as indicated in Results and Figure Legends.

N-glycan analysis using HILIC-UPLC

N-linked glycans were released from gp140 in-gel using PNGase F (New England Biolabs). The released glycans were subsequently fluorescently labeled with procainamide using 110 mg/ml procainamide and 60 mg/ml sodium cyanoborohydride. Excess label and PNGase F were removed using Spe-ed Amide-2 cartridges (Applied Separations). Glycans were analyzed on a Waters Acquity H-Class UPLC instrument with a Glycan BEH Amide column (2.1 mm x 100 mm, 1.7 μM, Waters). Fluorescence was measured, and data were processed using Empower 3 software (Waters, Manchester, UK). The relative abundance of oligomannose glycans was measured by digestion with Endoglycosidase H (Endo H; New England Biolabs). Digestion was performed overnight at 37 degrees. Digested glycans were cleaned using a PVDF protein-binding membrane (Millipore) and analyzed as described above.

Site-specific glycan analysis using mass spectrometry

Env proteins were denatured for 1h in 50 mM Tris/HCl, pH 8.0 containing 6 M of urea and 5 mM dithiothreitol (DTT). Next, the Env proteins were reduced and alkylated by adding 20 mM iodacetamide (IAA) and incubated for 1h in the dark, followed by a 1h incubation with 20 mM DTT to eliminate residual IAA. The alkylated Env proteins were buffer-exchanged into 50 mM Tris/HCl, pH 8.0 using Vivaspin columns (3 kDa) and digested separately overnight using trypsin, chymotrypsin or subtilisin (Mass Spectrometry Grade, Promega) at a ratio of 1:30 (w/w). The next day, the peptides were dried and extracted using C18 Zip-tip (MerckMilipore). The peptides were dried again, re-suspended in 0.1% formic acid and analyzed by nanoLC-ESI MS with an Easy-nLC 1200 (Thermo Fisher Scientific) system coupled to a Fusion mass spectrometer (Thermo Fisher Scientific) using higher energy collision-induced

CellPress
OPEN ACCESS

dissociation (HCD) fragmentation. Peptides were separated using an EasySpray PepMap RSLC C18 column (75 μm x 75 cm). The LC conditions were as follows: 275-minute linear gradient consisting of 0%–32% acetonitrile in 0.1% formic acid over 240 minutes followed by 35 minutes of 80% acetonitrile in 0.1% formic acid. The flow rate was set to 200 nL/min. The spray voltage was set to 2.7 kV and the temperature of the heated capillary was set to 40°C. The ion transfer tube temperature was set to 275°C. The scan range was 400-1600 m/z. The HCD collision energy was set to 50%, appropriate for fragmentation of glycopeptide ions. Precursor and fragment detection were performed using an Orbitrap at a resolution MS1 = 100,000. MS2 = 30,000. The AGC target for MS1 = $4e^5$ and MS2 = $5e^4$ and injection time: MS1 = 50ms MS2 = 54ms

Glycopeptide fragmentation data were extracted from the raw file using Byonic™ (Version 2.7) and Byologic™ software (Version 2.3; Protein Metrics Inc.). The glycopeptide fragmentation data were evaluated manually for each glycopeptide; the peptide was scored as true-positive when the correct b and y fragment ions were observed along with oxonium ions corresponding to the glycan identified. The MS data was searched using a standard library for HEK293F expressed BG505 SOSIP.664 (Seabright et al., 2019). The precursor mass tolerance was set at 4ppm for MS1 and 10ppm for MS2. A 1% false discovery rate (FDR) was applied. The relative amounts of each glycan at each site as well as the unoccupied proportion were determined by comparing the extracted ion chromatographic areas for different glycopeptides with an identical peptide sequence. Glycans were categorized according to the composition detected. HexNAc(2)Hex(9−5) was classified as M9 to M5. HexNAc(3)Hex(5−6)X was classified as Hybrid with HexNAc(3)Fuc(1)X classified as Fhybrid. Complex-type glycans were classified according to the number of processed antenna and fucosylation. If all of the following compositions have a fucose they are assigned into the FA categories. HexNAc(3)Hex(3-4)X is assigned as A1, HexNAc(4)X is A2/A1B, HexNAc(5)X is A3/A2B, and HexNAc(6)X is A4/A3B. As this fragmentation method does not provide linkage information isomers are grouped, so for example a triantennary glycan contains HexNAc (5) but so does a biantennary glycan with a bisect.

Site-specific analysis of low abundance N-glycan sites using mass spectrometry

To obtain data for sites that frequently present low intensity glycopeptide the glycans present on the glycopeptides were homogenized to boost the intensity of these peptides. This analysis loses fine processing information but enables the ratio of oligomannose: complex: unoccupied to be determined. The remaining glycopeptides were first digested with Endo H (New England Biolabs) to deplete oligomannose- and hybrid-type glycans and leave a single GlcNAc residue at the corresponding site. The reaction mixture was then dried completely and resuspended in a mixture containing 50 mM ammonium bicarbonate and PNGase F (New England Biolabs) using only H_2O^{18} (Sigma-Aldrich) throughout. This second reaction cleaves the remaining complex-type glycans but leaves the GlcNAc residues remaining after Endo H cleavage intact. The use of H_2O^{18} in this reaction enables complex glycan sites to be differentiated from unoccupied glycan sites as the hydrolysis of the glycosidic bond by PNGaseF leaves a heavy oxygen isotope on the resulting aspartic acid residue. The resultant peptides were purified as outlined above and subjected to reverse-phase (RP) nanoLC-MS. Instead of the extensive N-glycan library used above, two modifications were searched for: +203 Da corresponding to a single GlcNAc, a remnant of an oligomannose/hybrid glycan, and +3 Da corresponding to the O^{18} deamidation product of a complex glycan. A lower HCD energy of 27% was used as glycan fragmentation was not required. Data analysis was performed as above and the relative amounts of each glycoform determined, including unoccupied peptides.

QUANTIFICATION AND STATISTICAL ANALYSIS

The integration of peaks corresponding to fluorescently labeled N-glycans shown in Figures 1–3 were performed using Empower 3.0 (Waters). ELISA binding data shown in Figure 2D was plotted and the mean, SEM, AUC and Mann-Whitney 2-tailed test were calculated using GraphPad Prism v8. ELISA binding data shown in Figures 2G and S2F were plotted and a Spearman's correlation coefficient was calculated using GraphPad Prism v8. Chromatographic areas were extracted for site-specific analysis, displayed in Figures 1–3 and 5, using Byonic™ (Version 2.7) and Byologic™ software (Version 2.3) by Protein Metrics.

Cell Reports, Volume *35*

Supplemental information

Enhancing glycan occupancy of soluble HIV-1

envelope trimers to mimic the native viral spike

Ronald Derking, Joel D. Allen, Christopher A. Cottrell, Kwinten Sliepen, Gemma E. Seabright, Wen-Hsin Lee, Yoann Aldon, Kimmo Rantalainen, Aleksandar Antanasijevic, Jeffrey Copps, Anila Yasmeen, Albert Cupo, Victor M. Cruz Portillo, Meliawati Poniman, Niki Bol, Patricia van der Woude, Steven W. de Taeye, Tom L.G.M. van den Kerkhof, P.J. Klasse, Gabriel Ozorowski, Marit J. van Gils, John P. Moore, Andrew B. Ward, Max Crispin, and Rogier W. Sanders

Supplementary Figures

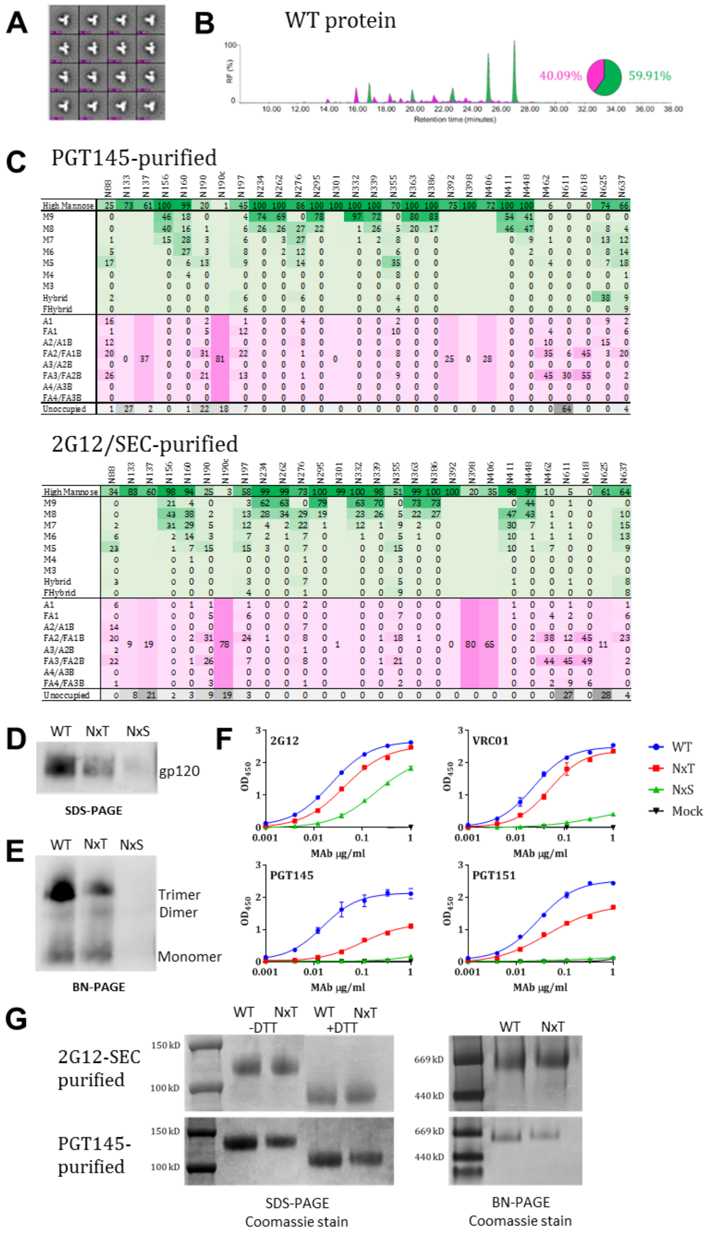

Figure S1. Several PNGS on BG505 SOSIP.v4.1 trimers are underoccupied. Related to Figures 1 and 2. (A) NS-EM analysis of 2G12/SEC-purified WT proteins, showing the 2D class-averages. **(B)** HILIC-UPLC analysis of the 2G12/SEC-purified WT protein. The color coding of the spectra and pie chart is the same as in Figure 1E **(C)** The data sets show the glycoforms found at each PNGS. Data for oligomannose/hybrid-type glycans are shaded in green, fully processed complex type glycans are shaded in magenta, while the absence of a glycan from some PNGS is shaded in grey. Oligomannose-type glycans are categorized according to the number of mannose residues present, hybrids by the presence/absence of fucose and complex-type glycans by the number of processed antenna and the presence/absence of fucose. For further information see Methods. Data that could only be obtained from low intensity peptides cannot be allocated into the above categories. They are merged to cover all oligomannose/hybrid compositions or complex-type glycans. The data presented in this panel are the mean of two independent biological replicates of WT protein. **(D)** Reducing SDS-PAGE analysis of unpurified WT, NxT and NxS proteins expressed in 293T cells, followed by western blotting with the CA13 (ARP3119) MAb. **(E)** BN-PAGE analysis of the same proteins, blotted with the 2G12 bNAb. The trimer, dimer and monomer bands are indicated. **(F)** D7324-capture ELISA quantifying the binding of the bNAbs 2G12, VRC01, PGT145 and PGT151 to the WT, NxT and NxS proteins. **(G)** Non-reducing and reducing (+ DTT) SDS-PAGE followed by Coomassie staining (left panel), and BN-PAGE analysis followed by Coomassie blue staining (right panel) of PGT145- and 2G12/SEC-purified WT and NxT proteins, as indicated.

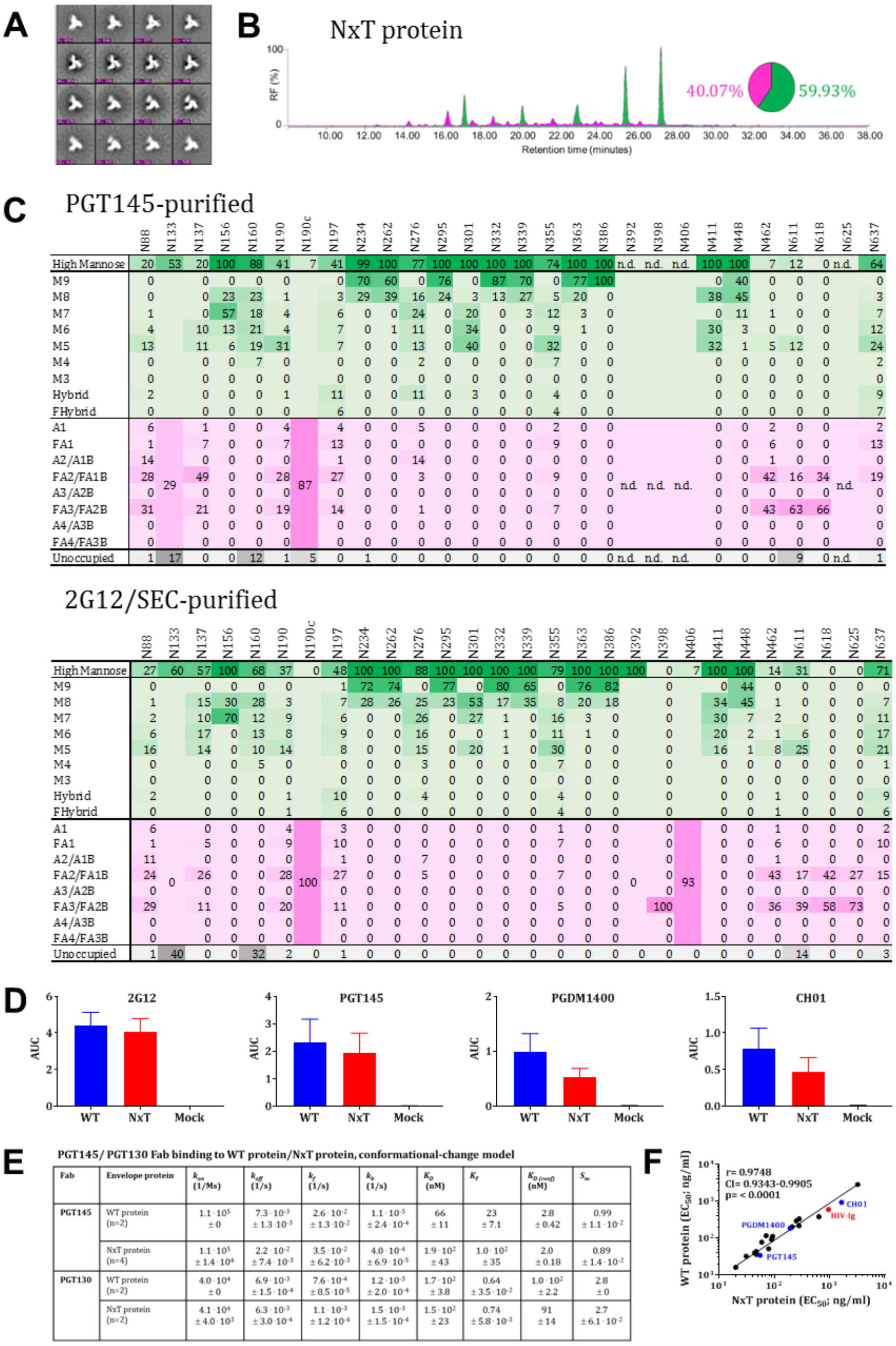

Figure S2. Glycan occupancy is enhanced by PNGS sequon engineering. Related to Figure 2. (A) NS-EM analysis of NxT proteins, showing the 2D class-averages. **(B)** HILIC-UPLC analysis of the NxT protein. The color coding of the spectra and pie chart is the same as in Figure S1B. **(C)** Quantification of site-specific occupancy and composition for 28 PNGS on NxT trimers, purified using the 2G12/SEC and PGT145 methods as indicated. The data are derived from LC-ESI MS experiments. The data set shows the glycoforms found at each PNGS. The relative under-occupancy and oligomannose and complex/hybrid content at each individual site are summarized, using the same color coding as in Figure S1C. **(D)** Binding of WT and NxT proteins to three V2-apex directed bNAbs, PGT145, PGDM1400 and CH01, and also 2G12 for comparison. AUC values derived from derived from ELISA titration curves are plotted. **(E)** Summary of the SPR binding kinetics of bNAbs PGT145 and PGT130 using data shown in Figure 2F. **(F)** The EC_{50} values derived using WT and NxT proteins were plotted and compared using Spearman's correlation coefficient. Binding data were generated for a panel of bNAbs spanning all major bNAb epitope clusters (Figure S3). All analyses were performed on NxT proteins produced in HEK293F followed by 2G12/SEC purification.

A

			Binding EC_{50} (ng/ml) PGT145 purified protein			Binding (AUC) PGT145 purified protein		
	Epitope	Antibody	WT protein	NxT protein	Fold difference	WT protein	NxT protein	Fold difference
Nabs	CD4bs	VRC01	66	100	1.5	3.2	2.9	0.9
		3BNC60	57	81	1.4	3.2	2.8	0.9
	V1/V2-glycan	PG9	388	359	0.9	3.0	2.7	0.9
		PG16	144	109	0.8	2.0	1.9	1.0
		PGT145	40	74	1.9	2.8	2.2	0.8
		PGDM1400	206	585	2.8	1.1	0.5	**0.5**
		CH01	372	906	2.4	2.2	1.4	**0.6**
	V3-glycan	PGT121	316	417	1.3	2.8	2.5	0.9
		PGT122	577	799	1.4	2.3	2.0	0.8
		PGT123	232	396	1.7	3.2	2.7	0.8
		PGT125	150	115	0.8	3.5	3.4	1.0
		PGT126	113	84	0.7	3.1	2.8	0.9
		PGT128	45	73	1.6	3.4	3.0	0.9
		PGT130	193	133	0.7	2.1	1.8	0.9
	OD-glycan	2G12	30	40	1.3	4.1	3.6	0.9
		PGT135	234	601	2.6	4.0	2.8	0.7
	gp120-gp41	PGT151	29	23	0.9	3.1	2.8	0.9
		35O22	814	2111	2.6	2.5	2.0	0.8
	gp41	3BC315	62	69	1.1	2.7	1.9	0.7
		HIV-IG	>10000	>10000	1.0	0.4	0.3	0.9

B

			Binding EC_{50} (ng/ml) 2G12/SEC purified protein			Binding (AUC) 2G12/SEC purified protein		
	Epitope	Antibody	WT protein	NxT protein	Fold difference	WT protein	NxT protein	Fold difference
Nabs	CD4bs	VRC01	36	47	1.3	3.6	3.3	0.9
		3BNC60	91	89	1.0	3.0	2.9	1.0
	V1/V2-glycan	PG9	201	211	1.1	2.9	3.1	1.1
		PG16	77	59	0.8	2.1	2.2	1.0
		PGT145	34	55	1.6	2.3	1.9	0.8
		PGDM1400	185	193	1.0	1.0	0.5	**0.5**
		CH01	923	1656	1.8	0.8	0.5	**0.6**
	V3-glycan	PGT121	291	250	0.9	2.3	2.4	1.0
		PGT122	333	282	0.8	2.0	1.7	0.9
		PGT123	216	280	1.3	2.2	1.8	0.9
		PGT125	51	79	1.5	3.5	3.3	0.9
		PGT126	32	31	1.0	4.1	4.1	1.0
		PGT128	43	47	1.1	4.1	3.8	0.9
		PGT130	111	93	0.8	2.0	1.8	0.9
	OD-glycan	2G12	16	20	1.3	4.4	4.0	0.9
		PGT135	379	644	1.7	3.7	2.7	0.8
	gp120-gp41	PGT151	40	44	1.1	1.8	1.5	0.8
		35O22	2829	3254	1.2	1.1	1.0	0.9
	gp41	3BC315	116	71	**0.6**	2.4	2.0	0.9
		HIV-IG	595	960	1.6	3.1	2.8	0.9

Figure. S3. Antigenic characterization of PGT145- and 2G12/SEC-purified WT and NxT proteins. Related to Figure 2. (A) Half-maximal binding concentrations (EC_{50}; in ng/ml) were derived from D7324-capture ELISAs using PGT145-purified WT and NxT proteins. The values represent the means of 4–10 independent single titration experiments for each bNAb. The fold-differences in EC_{50} values for NxT *vs.*WT proteins are listed. We also tabulated the average AUC values derived from the titration curves for each MAb. Bold numbers indicate values where the differences are <0.6- or >3-fold. **(B)** The same analysis and data presentation but derived using 2G12/SEC-purified WT and NxT proteins.

A

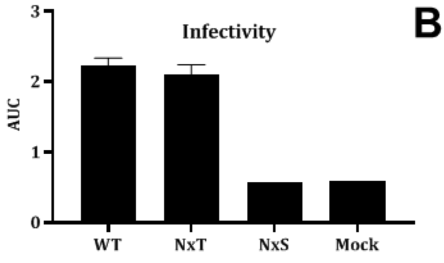

Virus	WT	NxT	NxS
CA-p24 (ng/ml)	127	1106	684

B

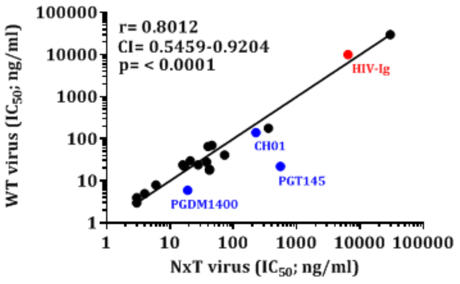

C

	Epitope	Antibody	Neutralization IC_{50} (ng/ml) WT virus	NxT virus	Fold difference	Neutralization (AUC) WT virus	NxT virus	Fold difference
Nabs	CD4bs	VRC01	66	40	0.6	238.2	200.7	0.8
		3BNC60	30	21	0.7	224.1	184.5	0.8
	V1/V2-glycan	PG9	19	42	2.2	155.0	181.2	1.2
		PG16	18	42	2.4	156.3	208.3	1.3
		PGT145	22	554	**25.7**	157.6	266.6	1.7
		PGDM1400	6	19	**3.1**	126.2	198.7	1.6
		CH01	140	227	1.6	203.8	279.1	1.4
	V3-glycan	PGT121	24	16	0.7	158.3	136.1	0.9
		PGT122	41	73	1.8	203.5	204.5	1.0
		PGT123	22	17	0.8	189.7	178.8	0.9
		PGT125	5	4	0.8	113.7	99.6	0.9
		PGT126	8	6	0.7	143.6	133.5	0.9
		PGT128	4	3	0.9	107.9	100.0	0.9
		PGT130	28	38	1.4	156.0	159.8	1.0
	OD-glycan	2G12	24	28	1.2	125.5	135.9	1.1
		PGT135	179	359	2.0	218.0	232.0	1.1
	gp120-gp41	PGT151	3	3	1.1	88.2	107.7	1.2
		35O22	70	46	0.6	144.7	117.8	0.8
	gp41	3BC315	>150000	>150000	1.0	359.3	372.1	1.0
		HIV-Ig	10086	6437	1.6	405.7	352.7	0.9

D

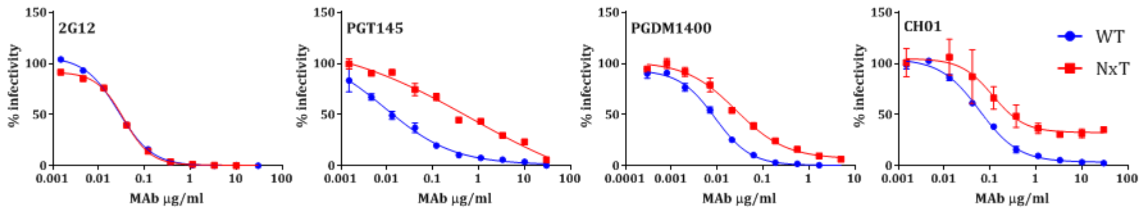

Figure S4. NxT sequon engineering is compatible with Env function and virus infectivity. Related to Figure 2. (A) Infection of TZM-bl reporter cells by WT, NxT and NxS viruses using a range of p24 concentrations (500, 250, 125, 62.5, 31.25, 16.62 and 7.81 pg). The AUC values derived from the titration curve were plotted. The box represents CA-p24 measured in each virus preparation. **(B)** Correlation between neutralization of WT and NxT viruses. The midpoint neutralization concentrations (IC_{50}) were plotted and the Spearman's correlation coefficient, r, was calculated. The outliers, PGT145, PGDM1400 and CH01, are indicated in blue. Polyclonal HIV-Ig is indicated in red. **(C)** Midpoint neutralization concentrations (IC_{50}; in ng/ml) were derived from single cycle infection experiments using TZM-bl cells and he indicated bNAbs or HIV-Ig. The values are averages based on 2-4 independent antibody-titration experiments. The average AUC values derived from the neutralization curves are also shown, in the three right-most columns. **(D)** Representative neutralization curves for the WT and NxT virus and the 2G12, PGT145, PGDM1400 and CH01 bNAbs.

A

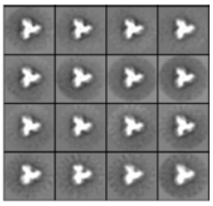

B

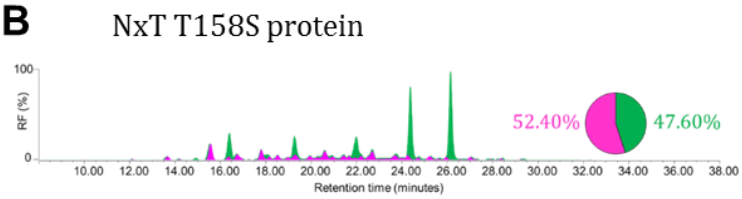

C

PGT145-purified

	N88	N133	N137	N156	N160	N190	N190c	N197	N234	N262	N276	N295	N301
High Mannose	11	65	17	100	100	25	4	38	100	100	63	100	98
M9	0	1	0	2	0	0		0	69	69	0	77	0
M8	0	22	1	25	10	1		3	25	29	5	23	28
M7	1	15	1	39	19	4		5	3	2	6	0	39
M6	2	12	1	14	25	4		3	2	0	5	0	12
M5	6	13	1	16	31	13		10	0	0	5	0	14
M4	0	1	0	1	12	0		0	0	0	2	0	0
M3	0	0	0	0	3	0		0	0	0	0	0	0
Hybrid	2	2	9	1	0	2		8	0	0	38	0	3
FHybrid	1	0	5	0	0	1		7	0	0	2	0	1
A1	3	1	0	0	0	1		2	0	0	3	0	0
FA1	1	0	2	0	0	4		9	0	0	0	0	0
A2/A1B	7	0	4	0	0	0		1	0	0	26	0	0
FA2/FA1B	22	4	36	0	0	33	96	34	0	0	4	0	1
A3/A2B	2	0	17	0	0	0		0	0	0	3	0	0
FA3/FA2B	39	4	20	0	0	32		15	0	0	0	0	1
A4/A3B	0	0	0	0	0	0		0	0	0	0	0	0
FA4/FA3B	14	0	4	0	0	4		0	0	0	0	0	0
Unoccupied	1	26	0	0	0	1	0	0	0	0	0	0	0

	N332	N339	N355	N363	N386	N392	N398	N406	N411	N448	N462	N611	N618	N625	N637
High Mannose	100	100	64	100	100	100	nd	99	100	100	7	10	0	3	51
M9	81	67	0	78	69				0	33	0	0	0	0	0
M8	15	28	3	19	26				22	35	0	0	0	0	1
M7	4	5	9	2	5				33	20	1	2	0	0	4
M6	1	1	11	1	0				24	8	1	0	0	0	7
M5	0	0	24	0	0				19	3	4	8	0	3	11
M4	0	0	9	0	0				0	1	0	0	0	0	1
M3	0	0	0	0	0				0	0	0	0	0	0	0
Hybrid	0	0	6	0	0				2	0	1	0	0	0	11
FHybrid	0	0	2	0	0				0	0	1	0	0	0	15
A1	0	0	0	0	0				0	0	1	0	0	0	1
FA1	0	0	26	0	0				0	0	3	0	0	0	8
A2/A1B	0	0	0	0	0				0	0	5	0	0	1	0
FA2/FA1B	0	0	5	0	0	0	n.d.	1	0	0	38	10	25	29	31
A3/A2B	0	0	0	0	0				0	0	1	0	0	0	0
FA3/FA2B	0	0	4	0	0				0	0	40	59	45	46	6
A4/A3B	0	0	0	0	0				0	0	0	0	0	0	0
FA4/FA3B	0	0	0	0	0				0	0	5	13	29	21	0
Unoccupied	0	0	0	0	0	0	nd	nd	0	0	0	8	0	0	2

2G12/SEC-purified

	N88	N133	N137	N156	N160	N190	N190c	N197	N234	N262	N276	N295	N301
High Mannose	17	75	n.d.	100	75	30	n.d.	40	95	100	31	100	n.d.
M9	0	0		20	2	0		1	61	65	0	63	
M8	1	24		37	45	3		5	28	28	0	29	
M7	2	19		30	15	6		5	4	3	1	3	
M6	4	11		7	4	4		4	2	2	2	1	
M5	9	17		5	3	13		10	1	1	4	2	
M4	0	1		1	0	0		0	0	0	2	1	
M3	0	0		0	0	0		0	0	0	0	0	
Hybrid	2	2		0	5	2		7	0	0	20	0	
FHybrid	1	1		0	1	2		7	0	0	3	0	
A1	2	1		0	2	1		2	0	0	1	0	
FA1	1	1		0	0	5		10	0	0	1	0	
A2/A1B	8	0		0	0	0		1	0	0	13	0	
FA2/FA1B	22	5	n.d.	0	15	30	n.d.	33	4	0	21	0	n.d.
A3/A2B	3	0		0	0	0		0	0	0	2	0	
FA3/FA2B	34	4		0	7	29		13	1	0	25	0	
A4/A3B	0	0		0	0	0		0	0	0	0	0	
FA4/FA3B	11	0		0	0	4		1	0	0	4	0	
Unoccupied	1	13	n.d.	0	0	1	n.d.	0	0	0	0	0	n.d.

	N332	N339	N355	N363	N386	N392	N398	N406	N411	N448	N462	N611	N618	N625	N637
High Mannose	99	88	73	97	45	0	n.d.	n.d.	100	94	11	24	1	69	66
M9	78	56	0	73	32	0			0	37	0	0	0	0	0
M8	18	24	8	17	12	0			35	38	1	1	0	14	6
M7	2	5	14	3	0	0			32	10	2	4	0	13	9
M6	0	1	9	1	0	0			19	3	1	3	0	9	13
M5	0	0	26	1	0	0			13	3	6	13	0	7	16
M4	0	0	1	0	0	0			0	1	0	0	0	0	1
M3	0	0	0	0	0	0			0	0	0	0	0	0	0
Hybrid	0	0	6	0	0	0			2	1	1	2	0	25	10
FHybrid	0	1	8	0	1	0			0	1	1	1	0	2	11
A1	0	0	1	0	0	0			0	0	1	1	0	2	1
FA1	0	0	7	0	0	0			0	1	3	2	0	3	8
A2/A1B	0	0	0	0	2	0			0	0	1	0	0	10	0
FA2/FA1B	0	5	11	2	23	2	n.d.	n.d.	0	4	43	13	22	7	19
A3/A2B	0	0	0	0	0	0			0	0	1	0	0	2	0
FA3/FA2B	0	6	7	1	27	65			0	1	36	44	46	6	5
A4/A3B	0	0	0	0	0	0			0	0	0	0	0	0	0
FA4/FA3B	0	1	1	0	4	33			0	0	5	12	31	0	1
Unoccupied	0	0	0	0	0	0	n.d.	n.d.	0	0	0	3	0	0	0

Figure. S5. N160 occupancy can be increased by reducing the affinity of a neighboring site for OST. Related to Figure 3. (A) NS-EM analysis of NxT 158S trimers, showing the 2D class-averages. **(B)** HILIC-UPLC analysis of the NxT T158S protein. The color coding of the spectra and pie chart is the same as in Figure S1B. **(C)** Quantification of site-specific occupancy and composition for 28 PNGS on NxT T158S proteins, purified using the 2G12/SEC and PGT145 methods as indicated. The data are derived from LC-ESI MS experiments. The data set shows the glycoforms found at each PNGS. The relative under-occupancy and oligomannose and complex/hybrid content at each individual site are summarized, using the same color coding as in Figure S1C.

A

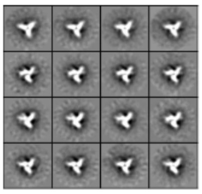

B PGT145-purified

	N88	N133	N137	N156	N160	N190	N190c	N197	N234	N262	N276	N295	N301	N332	N339	N355	N363	N386	N392	N398	N406	N411	N448	N462	N611	N618	N625	N637
High Mannose	10	90	65	100	100	0	0	30	99	100	76	100	98	100	93	61	100	100	n.d.	n.d.	36	100	100	3	6	0	n.d.	52
M9	0			37	0			0	70	63	0	88		91	61	0	77	81				0	48	0	0	0		0
M8	0			46	33			1	24	29	10	12		8	27	3	21	19				45	39	0	0	0		0
M7	1			12	17			3	3	4	16	0		1	3	8	2	0				15	8	0	1	0		6
M6	2			3	20			3	2	4	15	0		0	2	7	1	0				26	4	0	1	0		5
M5	6			2	31			10	0	0	19	0		0	0	24	0	0				14	1	2	4	0		7
M4	0			0	0			0	0	0	2	0		0	0	1	0	0				0	0	0	0	0		1
M3	0			0	0			0	0	0	0	0		0	0	0	0	0				0	0	0	0	0		0
Hybrid	1			1	0			6	0	0	14	0		0	0	6	0	0				0	0	0	0	0		6
FHybrid	1			0	0			7	0	0	1	0		0	0	11	0	0				0	0	1	0	0		27
A1	3			0	0			2	0	0	5	0		0	0	1	0	0				0	0	0	0	0		1
FA1	1			0	0			11	0	0	0	0		0	0	15	0	0				0	0	4	1	0		10
A2/A1B	10			0	0			1	0	0	14	0		0	0	0	0	0				0	0	1	0	0		0
FA2/FA1B	29	5	35	0	0	100	100	38	0	0	2	0	2	0	0	12	0	0	n.d	n.d	64	0	0	43	14	28	n.d.	27
A3/A2B	5			0	0			0	0	0	2	0		0	0	0	0	0				0	0	0	0	0		0
FA3/FA2B	35			0	0			16	0	0	0	0		0	0	9	0	0				0	0	43	55	60		9
A4/A3B	0			0	0			0	0	0	0	0		0	0	0	0	0				0	0	0	0	0		0
FA4/FA3B	8			0	0			1	0	0	0	0		0	0	1	0	0				0	0	5	20	10		0
Unoccupied	0	6	0	0	0	0	0	1	1	0	0	0	0	0	7	0	0	0	n.d.	n.d.	0	0	0	0	3	2	n.d.	2

2G12/SEC-purified

	N88	N133	N137	N156	N160	N190	N190c	N197	N234	N262	N276	N295	N301	N332	N339	N355	N363	N386	N392	N398	N406	N411	N448	N462	N611	N618	N625	N637
High Mannose	7	62	39	99	99	25	0	35	80	99	36	100	100	100	87	68	98	85	n.d.	n.d.	0	100	92	7	10	1	65	60
M9	0			24	9	0		1	52	66	0			82	64	0	77	68				0	39	0	0	0		0
M8	0			33	27	2		3	18	25	3			14	19	6	16	17				38	35	0	1	0		5
M7	0			27	21	3		6	4	4	6			2	3	13	3	0				29	11	1	2	0		7
M6	1			7	15	3		3	3	3	4			1	0	9	1	0				16	4	1	1	0		1
M5	5			6	19	17		11	2	2	4			2	0	26	1	0				17	2	4	6	0		29
M4	0			0	7	0		0	0	0	1			0	0	1	0	0				0	0	0	0	0		0
M3	0			0	1	0		0	0	0	0			0	0	0	0	0				0	0	0	0	0		0
Hybrid	0			1	0	1		6	0	0	15			0	0	6	0	0				0	1	0	0	0		7
FHybrid	1			0	0	0		5	1	0	2			0	1	6	0	0				0	1	1	1	1		10
A1	0			1	0	1		2	0	0	2			0	0	1	0	0				0	0	1	1	0		3
FA1	2			0	0	5		10	1	0	1			0	0	10	0	0				0	1	5	8	0		14
A2/A1B	8			0	0	0		1	0	0	11			0	0	0	0	0				0	0	2	0	0		0
FA2/FA1B	31	0	60	1	1	18	100	35	12	1	19	0	0	0	7	10	1	7	n.d.	n.d.	91	0	5	41	15	41	21	21
A3/A2B	3			0	0	0		0	0	0	4			0	0	0	0	0				0	0	0	0	0		0
FA3/FA2B	41			0	0	33		17	7	0	23			0	6	7	0	9				0	2	40	49	46		1
A4/A3B	0			0	0	0		0	0	0	0			0	0	0	0	0				0	0	0	0	0		0
FA4/FA3B	7			0	0	3		1	1	0	5			0	1	3	0	0				0	0	4	15	12		0
Unoccupied	0	37	1	0	0	14	0	0	0	0	0	0	0	0	0	0	0	0	n.d.	n.d.	0	0	0	0	0	0	13	1

C

CHO BG505 WT	N88	N133	N137	N156	N160	N190	N190c	N197	N234	N262	N276	N295	N301	N332	N339	N355	N363	N386	N392	N398	N406	N411	N448	N462	N611	N618	N625	N637
High Mannose	2	83	69	92	100	0	0	19	100	100	99	100	100	100	92	68	100	100	n.d.	n.d.	0	99	100	0	0	n.d.	0	38
Complex	98	0	15	0	0	8	62	48	0	0	1	0	0	0	0	32	0	0	n.d.	n.d.	100	1	0	100	37	n.d.	0	45
Unoccupied	0	17	16	8	0	92	38	34	0	0	0	0	0	0	8	0	0	0	n.d.	n.d.	0	0	0	0	63	n.d.	100	16
**CHO BG505 NxT**																												
High Mannose	0	86	66	100	100	0	0	0	100	100	92	100	100	100	100	88	100	100	n.d.	n.d.	n.d	n.d.	100	0	0	0	0	92
Complex	100	6	18	0	0	93	100	100	0	0	8	0	0	0	0	12	0	0	n.d.	n.d.	n.d	n.d.	0	100	100	100	0	0
Unoccupied	0	8	16	0	0	7	0	0	0	0	0	0	0	0	0	0	0	0	n.d.	n.d.	n.d	n.d.	0	0	0	0	100	8
Change in occupancy	0	8	1	8	0	85	38	34	0	0	0	0	0	0	8	0	0	0	n.d.	n.d.	n.d	n.d.	0	0	63	n.d.	0	8

D

WT change in CHO	N88	N133	N137	N156	N160	N190	N190c	N197	N234	N262	N276	N295	N301	N332	N339	N355	N363	N386	N392	N398	N406	N411	N448	N462	N611	N618	N625	N637
High Mannose	-23	10	8	-8	1	-19	-1	-26	0	0	13	0	0	0	-8	-2	0	0	n.d	nd	-72	-1	0	-6	0	nd.	-74	-27
Complex	24	0	-23	0	0	-50	-19	0	0	0	-13	0	0	0	0	2	0	0	n.d	nd	72	1	0	6	1	nd.	-26	15
Unoccupied	-1	-10	14	8	-1	70	20	26	0	0	0	0	0	0	8	0	0	0	n.d	nd	0	0	0	0	-1	nd.	100	12

NxT change in CHO	N88	N133	N137	N156	N160	N190	N190c	N197	N234	N262	N276	N295	N301	N332	N339	N355	N363	N386	N392	N398	N406	N411	N448	N462	N611	N618	N625	N637
High Mannose	-10	-4	2	0	0	0	0	-30	1	0	16	0	2	0	7	27	0	0	n.d	nd	nd.	nd	0	-3	-6	0	nd	41
Complex	11	1	-17	0	0	-7	0	32	0	0	-16	0	-2	0	0	-27	0	0	n.d	nd	nd.	nd	0	3	9	2	nd	-46
Unoccupied	0	3	15	0	0	7	0	-1	-1	0	0	0	0	0	-7	0	0	0	n.d	nd	nd.	nd	0	0	-3	-2	nd	6

Figure. S6. N133 occupancy is increased by reducing its affinity for OST. Related to Figure 3. (A) NS-EM analysis of NxT T135S T158S proteins, showing the 2D class-averages. **(B)** Quantification of site-specific occupancy and composition for 28 PNGS on NxT T135S T158S proteins, purified using the 2G12/SEC and PGT145 methods as indicated. The data are derived from LC-ESI MS experiments. The data set shows the glycoforms found at each PNGS. The relative under-occupancy and oligomannose and complex/hybrid content at each individual site are summarized, using the same color coding as in Figure S1C. **(C)** Site-specific analysis of the WT and NxT T135S T158S proteins produced in ExpiCHO-S cells. **(D)** Site-specific changes in glycosylation when BG505 SOSIP WT is produced in ExpiCHO-S cells compared to HEK293F cells. A percentage point (p.p.) increase represents a higher abundance of a particular glycoform on ExpiCHO-S cells.

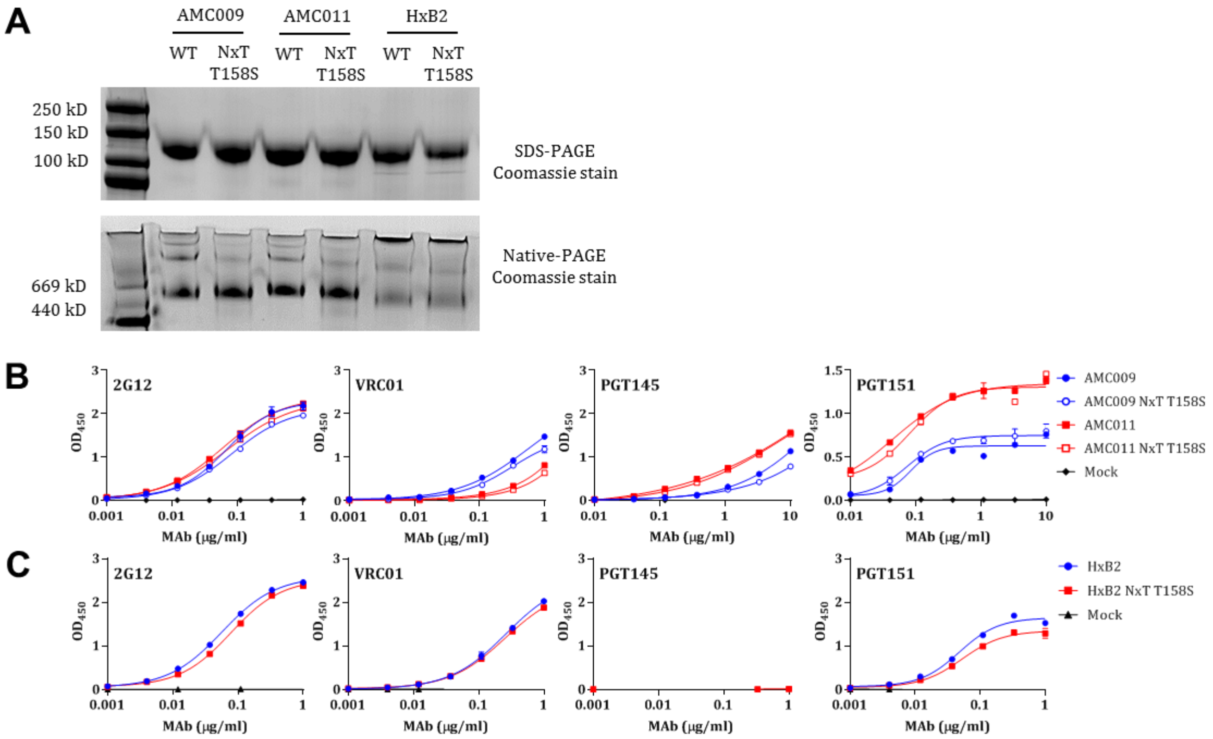

D

AMC009 WT	N88	N130	N136	N139	N141	N156	N160	N189A	N197	N234	N241	N262	N276	N289	N295	N301	N332	N339	N356	N362	N386	N392	N397	N448	N463	N611	N616	N625	N637
High mannose	37	95	54	14	5	90	87	37	42	98	98	100	99	90	100	99	99	100	15	100	100	100	9	100	4	25	26	19	23
Complex	62	5	40	4	91	0	12	62	36	0	0	0	1	0	0	0	1	0	85	0	0	0	91	0	96	16	18	80	12
Unoccupied	1	0	6	82	3	10	1	1	23	2	2	1	0	10	0	1	0	0	0	0	0	0	0	0	0	58	55	1	65
**NxT**																													
High mannose	37	100	70	6	3	88	92	20	22	98	97	100	93	100	100	100	99	100	0	100	100	100	0	100	0	1	2	18	12
Complex	55	0	26	85	94	0	3	75	73	0	2	0	7	0	0	0	0	0	100	0	0	0	100	0	100	84	94	82	18
Unoccupied	8	0	4	9	2	12	5	4	5	1	1	0	0	0	0	0	1	0	0	0	0	0	0	0	0	14	4	0	70
Change in occupancy	-7	0	2	73	1	-2	-4	-4	18	1	1	1	0	10	0	1	-1	0	0	0	0	0	0	0	0	44	51	1	-5

AMC011 WT	N88	N130	N136	N141	N141c	N156	N160	N188	N197	N234	N241	N262	N276	N289	N295	N301	N332	N339	N355	N362	N386	N392	N396	N406	N448	N461	N611	N616	N625	N637
High mannose	34	100	100	14	21	99	99	73	57	98	86	99	97	n.d.	99	99	100	100	22	97	100	n.d.	n.d.	100	98	36	3	3	19	44
Complex	65	0	0	8	79	0	1	26	41	0	0	0	3	n.d.	0	0	0	0	77	0	0	n.d.	n.d.	0	0	63	5	0	75	41
Unoccupied	1	0	0	78	0	1	0	1	2	2	14	1	0	n.d.	1	1	0	0	0	3	0	n.d.	n.d.	0	2	1	92	97	6	15
**NxT**																														
High mannose	50	96	100	18	0	88	99	89	81	99	99	100	29	99	91	95	100	87	23	100	100	100	100	99	100	25	9	14	57	35
Complex	41	1	0	82	79	0	0	8	19	0	1	0	71	0	0	5	0	0	77	0	0	0	0	1	0	75	88	6	39	27
Unoccupied	8	3	0	0	21	12	0	2	0	0	0	0	0	1	9	0	0	13	0	0	0	0	0	0	0	0	3	80	4	38
Change in occupancy	-7	-3	0	78	-21	-12	0	-2	2	2	14	1	0	n.d.	-8	1	0	-13	0	3	0	n.d.	n.d.	0	2	1	89	17	2	-23

HXB2 WT	N88	N136	N141	N156	N160	N189	N197	N230	N234	N241	N262	N276	N289	N295	N301	N322	N339	N356	N386	N392	N397	N406	N411	N448	N463	N611	N616	N624	N637
High Mannose	94	12	0	52	20	6	34	100	96	97	100	75	98	100	100	98	99	25	100	99	n.d.	7	n.d.	98	25	3	2	56	20
Complex	6	88	100	3	3	33	60	0	1	1	0	14	0	0	0	0	0	75	0	1	n.d.	93	n.d.	1	73	43	15	40	39
Unoccupied	0	0	0	45	77	61	5	0	2	2	0	11	1	0	0	1	0	0	0	0	n.d.	0	n.d.	0	2	54	84	3	41
**NxT**																													
High Mannose	86	14	3	54	66	5	30	100	97	96	100	77	100	99	28	95	97	11	99	99	100	0	100	94	11	2	14	58	20
Complex	14	86	97	13	22	42	69	0	3	3	0	20	0	1	70	5	3	89	1	1	0	100	0	6	89	89	68	42	64
Unoccupied	0	0	0	33	11	53	1	0	0	0	0	3	0	0	2	0	0	0	0	0	0	0	0	0	0	9	18	0	16
Change in occupancy	0	0	0	12	66	8	5	0	2	2	0	8	1	0	-2	1	0	0	0	0	n.d.	0	n.d.	0	2	46	65	3	25

Figure. S7. PNGS sequon engineering on diverse Env isolates. Related to Figure 5. (A) Reducing (+ DTT) SDS-PAGE followed by Coomassie staining (top panel), and BN-PAGE analysis followed by Coomassie blue staining (bottom panel) of PGT145-purified AMC009, AMC011 and HxB2 WT and NxT T158S proteins, as indicated. **(B)** D7324-capture ELISA quantifying the binding of the bNAbs 2G12, VRC01, PGT145 and PGT151 to the AMC009, AMC009 NxT T158S, AMC011 and AMC011 NxT T158S proteins. **(C)** Ni-NTA-capture ELISA quantifying the binding of the bNAbs 2G12, VRC01, PGT145 and PGT151 to the HxB2 and HxB2 NxT T158S proteins. **(D)** Glycoforms are grouped for all samples into high mannose (oligomannose- and hybrid-type), complex and unoccupied. The percentage change in occupancy between the WT protein and the NxT T158S protein is also shown.
